# Synthesis and Anticancer Activity of Dimeric Polyether Ionophores

**DOI:** 10.3390/biom10071039

**Published:** 2020-07-12

**Authors:** Michał Sulik, Ewa Maj, Joanna Wietrzyk, Adam Huczyński, Michał Antoszczak

**Affiliations:** 1 Department of Medical Chemistry, Faculty of Chemistry, Adam Mickiewicz University, Uniwersytetu Poznańskiego 8, 61–614 Poznań, Poland; michisulik@wp.pl (M.S.); adhucz@amu.edu.pl (A.H.); 2 Hirszfeld Institute of Immunology and Experimental Therapy, Polish Academy of Sciences, Rudolfa Weigla 12, 53–114 Wrocław, Poland; ewa.maj@hirszfeld.pl (E.M.); joanna.wietrzyk@hirszfeld.pl (J.W.)

**Keywords:** polyether ionophores, betulinic acid, stereoselective reactions, Huisgen 1,3-dipolar cycloaddition, ‘click’ chemistry, antiproliferative activity, in vitro assay, tumor-specificity, multi-drug resistance, SAR analysis

## Abstract

Polyether ionophores represent a group of natural lipid-soluble biomolecules with a broad spectrum of bioactivity, ranging from antibacterial to anticancer activity. Three seem to be particularly interesting in this context, namely lasalocid acid, monensin, and salinomycin, as they are able to selectively target cancer cells of various origin including cancer stem cells. Due to their potent biological activity and abundant availability, some research groups around the world have successfully followed semi-synthetic approaches to generate original derivatives of ionophores. However, a definitely less explored avenue is the synthesis and functional evaluation of their multivalent structures. Thus, in this paper, we describe the synthetic access to a series of original homo- and heterodimers of polyether ionophores, in which (i) two salinomycin molecules are joined through triazole linkers, or (ii) salinomycin is combined with lasalocid acid, monensin, or betulinic acid partners to form ‘mixed’ dimeric structures. Of note, all 11 products were tested in vitro for their antiproliferative activity against a panel of six cancer cell lines including the doxorubicin resistant colon adenocarcinoma LoVo/DX cell line; five dimers (**14**–**15**, **17**–**18** and **22**) were identified to be more potent than the reference agents (i.e., both parent compound(s) and commonly used cytostatic drugs) in selective targeting of various types of cancer. Dimers **16** and **21** were also found to effectively overcome the resistance of the LoVo/DX cancer cell line.

## 1. Introduction

**Figure 1:**
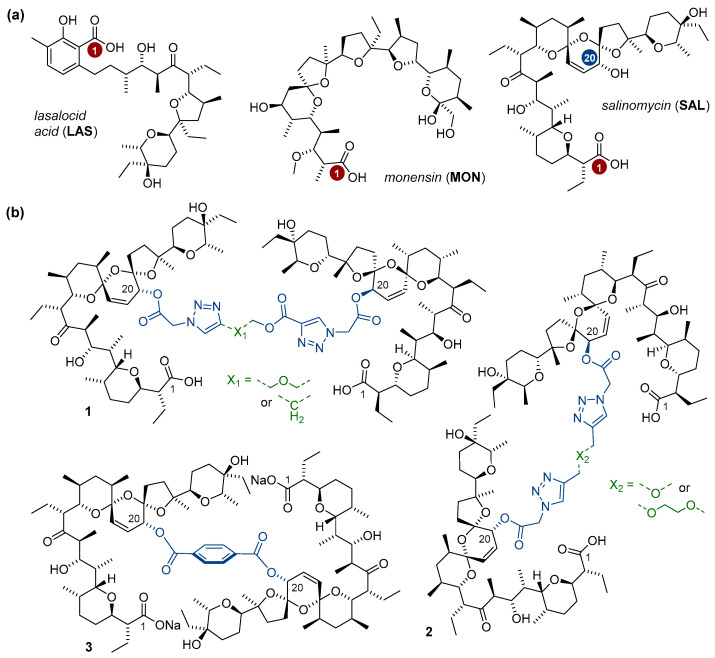
(**a**) Structures of polyether ionophores studied in this work, and (**b**) the literature-known salinomycin homodimers conjugated via the C20 hydroxyl [29,30].

With more than 120 structures reported until now, naturally-occurring polyether ionophores constitute a group of products that are characterized by a very broad spectrum of bioactivity including antibacterial, antifungal, antiparasitic, and antiviral activity [1,2,3,4]. Six of them have been approved for use in veterinary medicine, as they were found to effectively target Gram(+) bacterial strains in ruminants, and coccidial infections in poultry [2,5]. In addition to their extensively explored antibiotic activity, recent studies have also revealed that selected ionophores show significant anticancer properties. Particularly interesting in this context seem to be the three multifunctional compounds lasalocid acid (**LAS**), monensin (**MON**), and salinomycin (**SAL**) (Figure 1a).

Very briefly, the anticancer properties of **LAS** have been proven in the tests on a series of cancer cell lines including breast, colon, and lung adenocarcinoma cell lines [6]. Importantly, **LAS** was more active, and simultaneously, exhibited lower cytotoxic properties on non-tumor cells than those of the commonly used chemotherapeutic drug cisplatin [6]. Further studies confirmed the positive effects of **LAS** against human prostate cancer [7]. As far as prostate cancer is concerned, in a screening study of ~5000 well-known drugs and drug-like compounds, **MON** used at nanomolar concentrations was identified as one of only four agents that revealed the ability to selectively inhibit the cell growth of these cancer cells [8]. Some other authors have additionally shown that **MON** inhibited the proliferation of many other types of cancer cells including those of lymphoma as well as a chemoresistant variant of pancreatic cancer [9,10,11]. On the other hand, the anticancer activity of **SAL** was originally reported in 2009, when this substance was identified as the most potent agent from ~16,000 compounds screened in the selective targeting of breast cancer stem cells (CSCs) [12]. Of note, when used at relevant doses, **SAL** was well-tolerated by cancer patients [13]. Aside from its activity against breast cancer, **SAL** was also found to be effective in destroying other drug-sensitive and drug-resistant cancer types [14].

As polyether ionophores exhibit a broad spectrum of interesting pharmacological properties, at present, semi-synthetic derivatives of these natural products with improved activity profiles compared to those of the native structures are of top research interests. Although the site-specific modifications of ionophores are non-trivial with respect to their multifunctional nature and possible decomposition during synthesis, a few teams around the world have successfully followed synthetic approaches to generate **LAS**, **MON**, and **SAL** analogs; the modifications have been related mainly to the manipulations of either the C1 carboxyl of these biomolecules [6,15,16,17,18,19,20], or the C20 hydroxyl of **SAL** (Figure 1a) [21,22,23,24,25,26,27]. Nevertheless, a definitely less explored direction of research is the synthesis as well as functional evaluation of the multivalent structures of polyether ionophores. With respect to **LAS** and **MON**, only five synthetic di- and tripodants have been described in the literature [28], while for **SAL**, a relatively short series of its C1 and C20 homodimers has been known until now [19,29,30]. In this group of compounds, the most promising anticancer drug candidates seem to be those conjugated through the C20 hydroxyl of **SAL** (**1–2**, Figure 1b), as they exhibited superior activity compared not only to that of the starting material, but also to the widely used therapeutics [29]. On the other hand, the C*_2_*-symmetric dimer of **SAL** (**3**, Figure 1b) was less potent than the unmodified ionophore, except toward non-tumorigenic cells [30].

**Scheme 1:**
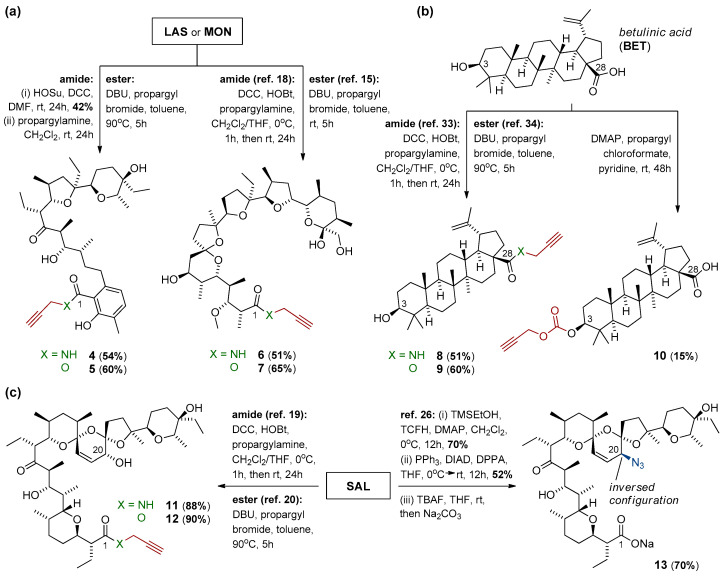
Synthesis of precursors of (**a**) lasalocid acid, monensin, (**b**) betulinic acid, and (**c**) salinomycin, as the partners for further CuAAC reactions.

Taking all of these facts into account, we decided to synthesize a completely new class of dimeric polyether ionophores, in which **SAL** would be linked at the C20 position not only with a second **SAL** molecule, but also with **LAS** or **MON** counterparts. Furthermore, to expand the structural diversity at the C20 position, we also conjugated **SAL** with a triterpenoid compound, betulinic acid (**BET**, Scheme 1b), as this biomolecule was identified as potently effective toward a wide variety of cancer cells like those derived from therapy-resistant and refractory tumors [31,32]. Simultaneously, **BET** was found to be relatively non-toxic to both non-malignant cells and normal tissues, indicating its very promising therapeutic window [31,32].

**Scheme 2:**
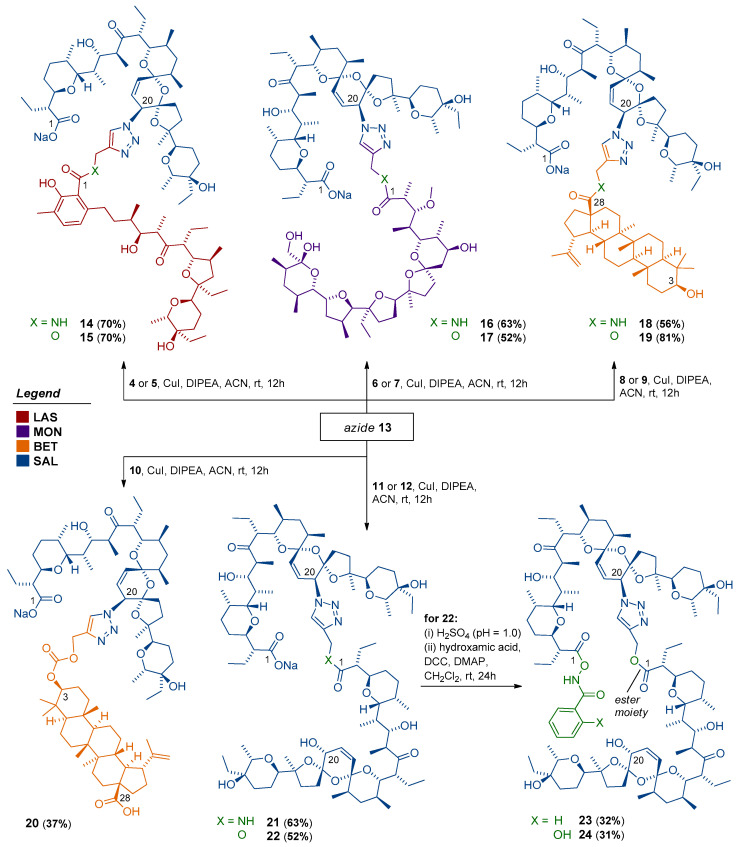
Synthetic access to novel dimeric polyether ionophores.

Synthetically, we applied the following strategies to obtain a library of completely novel homo- and heterodimers of polyether ionophores (Scheme 1 and Scheme 2): (i) design and synthesis of propargyl and azide precursors of **LAS**, **MON**, **BET**, and **SAL** (Scheme 1), and (ii) synthesis of a unique class of triazole-linked dimers using the Cu(I)-catalyzed azide-alkyne cycloaddition (CuAAC) (Scheme 2). In the next step, all 11 newly synthesized dimers were tested for their possible anticancer activity in cell-based assays. The in vitro activity evaluation toward six cancer cell lines showed that ester-triazole-linked dimers exhibited improved antiproliferative potency compared to that of the reference agents, while the corresponding dimers containing the amide-triazole linkers were active toward the cancer cell line with the multi-drug resistance (MDR) phenotype. Of note is that several of the newly synthesized compounds provided the opportunity for selective targeting of cancer cells; in many cases, their selectivity was much higher not only than that of the native structure(s), but also than those of the two commonly used anticancer drugs, cisplatin and doxorubicin. 

## 2. Materials and Methods 

### 2.1. General Procedures

Most reagents and solvents used for the synthesis of dimeric polyether ionophores and their precursors were purchased from commercial suppliers, and applied directly as received. Deuterated solvents for NMR analysis (CD_2_Cl_2_ and CDCl_3_) were stored over 3 Å molecular sieves for a few days before their first use. All synthetic work was performed under an inert atmosphere using oven-dried glassware. The progress of the reactions was monitored regularly with the use of commercially available aluminum-backed thin layer chromatography (TLC) plates. TLC plates (Merck 60 F_254_) were visualized in two ways; first, by UV light (λ = 254 nm), and second, using phosphomolybdic acid (PMA, 5% in absolute EtOH), which was followed by gentle heating. All synthesized compounds were purified on the automatic chromatographic system equipped with integrated UV and ELS detectors, with the use of HPLC grade solvents. Rotary evaporators were used to remove the organic solvents.

^1^H and ^13^C NMR spectra of the obtained products were recorded using a Varian 400 magnetic resonance spectrometer. ^1^H NMR spectra (at 400 MHz) are reported in chemical shifts in parts *per* million (ppm) downfield from Si(CH_3_)_4_, and with the use of the residual peak of the appropriate deuterated solvent as the internal standard (CD_2_Cl_2_ *δ* 5.32 ppm or CDCl_3_ *δ* 7.26 ppm). ^1^H NMR spectra of all newly synthesized compounds were thoroughly characterized by the following four parameters: chemical shift (*δ*, ppm), multiplicity (s = singlet, d = doublet, t = triplet, q = quartet, dd = doublet of doublets, dt = doublet of triplets, dq = doublet of quartets, ddd = doublet of doublet of doublets, td = triplet of doublets, m = multiplet), coupling constant(s) in Hz as well as integration. All peaks in the ^1^H NMR spectra are reported as described above, within the strongly overlapped region of ~2.00–0.50 ppm. ^13^C NMR spectra (at 101 MHz) of the obtained derivatives are reported in chemical shifts in ppm downfield from Si(CH_3_)_4_, and with the use of the residual peak of the appropriate deuterated solvent as the internal standard (CD_2_Cl_2_ *δ* 53.8 ppm or CDCl_3_ *δ* 77.2 ppm). 

On the other hand, infrared (IR) spectra of dimeric polyether ionophores and their precursors in the mid IR region were recorded in the form of KBr tablets with the use of an IFS 113v FT-IR spectrophotometer (Bruker), which was equipped with a DTGS detector. All obtained IR spectra were characterized by two parameters: wavenumbers (cm^–1^), and evaluation of the intensity of the appropriate bands (w = weak, m = medium, s = strong, br = broad). The IR spectra were taken on resolution 2 cm^–1^, NSS = 64, and the function of Happ–Genzel apodization was used.

Finally, electrospray ionization (ESI) mass spectra of all synthesized compounds were recorded with the use of a Waters/Micromass ZQ mass spectrometer (Waters Alliance), which was equipped with a Harvard syringe pump. The samples to be studied were prepared exclusively in anhydrous CH_3_CN, and were infused into the ESI source with the use of a Harvard pump at the flow rates of 20 mL min^–1^. The potentials of the ESI source were as follows: capillary 3 kV, lens 0.5 kV as well as extractor 4 V. ESI mass spectra of dimeric polyether ionophores and their precursors were recorded at the two cone voltages (CV) of 10 and 30 V. The other parameters included the source and the desolvation temperature of 120 °C and 300 °C, respectively, while nitrogen was used as the nebulizing as well as desolvation gas with the flow rates of 100 L h^–1^. Mass spectra of all synthesized compounds were acquired in the positive ion detection mode only, with unit mass resolution at a step of 1 *m/z* unit. The mass range for all performed ESI experiments was from *m/z* = 300 to *m/z* = 2000.

### 2.2. Synthesis

Lasalocid acid (**LAS**), monensin (**MON**), and salinomycin (**SAL**) were prepared conveniently by isolation of their sodium salts from commercially available veterinary premixes (Avatec^®^, Coxidin^®^, and Sacox^®^, respectively), followed by acidic extraction with H_2_SO_4_ (pH = 1.0), according to the procedures described previously [6,15,19,20]. Betulinic acid (**BET**) was purchased from Betulinines (Czech Republic). The respective *N*-propargyl amides and propargyl esters of **MON**, **BET**, and **SAL** (compounds **6**–**9** and **11**–**12**, Scheme 1) as well as an azide component (compound **13**, Scheme 1c) were synthesized using the procedures described thoroughly in the literature [15,18,19,20,26,33,34] or with some minor modifications (please see Paragraph 3.1 and/or Scheme 1 for more details). The NMR data of all of these compounds were in good agreement with those found in the reference literature.

#### 2.2.1. Synthesis of *N*-Propargyl Amide of Lasalocid Acid (Compound **4**)

To a stirred solution of **LAS** (1.02 g, 1.73 mmol, 1.0 equiv.) in anhydrous DMF (15 mL) at room temperature HOSu (199 mg, 1.73 mmol, 1.0 equiv.) was added in one portion, followed by the addition of DCC (464 mg, 2.26 mmol, 1.3 equiv.). The resulting solution was stirred at room temperature for the next 24 h. After that, the reaction mixture was concentrated under reduced pressure, and purified chromatographically using the CombiFlash system (0 → 50% EtOAc/*n*-hexane) to give 510 mg of activated ester of **LAS** (42% yield) isolated as a white amorphous solid. This ester was then dissolved in anhydrous CH_2_Cl_2_ (10 mL) at room temperature, and propargylamine (286 mg, 5.20 mmol, 3.0 equiv.) was introduced dropwise over 30 s. The resulting orange solution was stirred at room temperature for 24 h. After this time, the reaction mixture was concentrated under reduced pressure. Purification on silica gel using the CombiFlash system (0 → 50% EtOAc/*n*-hexane) gave the pure product of the reaction **4** (54% yield) as clear oil. The oil was diluted in *n*-pentane and evaporated to dryness three times to form an amorphous solid. The ^1^H and ^13^C NMR spectra of amide **4** are included in the Supplementary Materials (Figures S1 and S2).

Yield: 270 mg, 23% over two steps. Isolated as a white amorphous solid, >95% pure by NMR, and a single spot by TLC; R*_f_*: 0.70 in 50% EtOAc/*n*-hexane. UV-active and strains green with PMA; ^1^H NMR (400 MHz, CD_2_Cl_2_) *δ* 10.04 (s, 1H), 7.13–7.07 (m, 1H), 6.86 (t, *J* = 5.3 Hz, 1H), 6.68 (d, *J* = 7.7 Hz, 1H), 4.32 (s, 1H), 4.19 (dd, *J* = 5.6, 2.5 Hz, 2H), 4.09 (dd, *J* = 9.6, 1.4 Hz, 1H), 3.93 (dd, *J* = 10.2, 4.2 Hz, 1H), 3.69 (dd, *J* = 13.7, 6.8 Hz, 2H), 3.40 (dd, *J* = 11.7, 2.0 Hz, 1H), 2.97 (dq, *J* = 9.6, 7.1 Hz, 1H), 2.91–2.80 (m, 1H), 2.70–2.59 (m, 2H), 2.32 (t, *J* = 2.5 Hz, 1H), 2.27–2.07 (m, 4H), 2.03–0.60 (m, 36H) ppm; ^13^C NMR (101 MHz, CD_2_Cl_2_) *δ* 212.9, 170.6, 157.0, 138.9, 133.3, 123.7, 121.5, 117.6, 87.1, 85.2, 80.1, 77.7, 73.2, 71.30, 71.27, 70.6, 54.4, 48.2, 38.7, 37.1, 34.9, 34.1, 31.5, 31.02, 31.01, 30.1, 29.6, 21.0, 16.9, 15.8, 15.5, 14.3, 13.7, 12.9, 12.5, 8.9, 6.5 ppm; FT-IR (KBr tablet): 3438 (br, s), 3313 (s), 2965 (s), 2938 (s), 2878 (s), 2125 (w), 1713 (s), 1631 (s), 1615 (s), 1578 (m), 1519 (s), 1487 (m), 1458 (s), 1414 (s) cm^–1^; ESI MS (*m/z*) [M + Na]^+^ Calcd for C_37_H_57_NNaO_7_ 650; Found 650.

#### 2.2.2. Synthesis of Propargyl Ester of Lasalocid Acid (Compound **5**)

A mixture of **LAS** (2.00 g, 3.39 mmol, 1.0 equiv.), DBU (775 mg, 5.09 mmol, 1.5 equiv.), and propargyl bromide (~80% in toluene) (1.21 g, 10.17 mmol, 3.0 equiv.) in anhydrous toluene (30 mL) was heated at 90 °C for 5 h. After that, the reaction mixture was concentrated under reduced pressure. Purification on silica gel using the CombiFlash system (0 → 50% EtOAc/*n*-hexane) gave the pure product of the reaction **5** (60% yield) as a clear oil. The oil was diluted in *n*-pentane and evaporated to dryness three times to form an amorphous solid. The ^1^H and ^13^C NMR spectra of ester **5** are included in the Supplementary Materials (Figures S3 and S4).

Yield: 1.28 g, 60%. Isolated as a white amorphous solid, >95% pure by NMR, and a single spot by TLC; R*_f_*: 0.86 in 50% EtOAc/*n*-hexane. UV-active and strains green with PMA; ^1^H NMR (400 MHz, CDCl_3_) *δ* 11.18 (s, 1H), 7.16 (dd, *J* = 7.6, 0.6 Hz, 1H), 6.67 (d, *J* = 7.6 Hz, 1H), 4.98 (t, *J* = 2.5 Hz, 2H), 3.97 (dd, *J* = 9.4, 2.0 Hz, 1H), 3.81 (dd, *J* = 10.0, 3.9 Hz, 1H), 3.75 (q, *J* = 6.9 Hz, 1H), 3.44 (dd, *J* = 11.5, 2.1 Hz, 1H), 3.27 (s, 2H), 3.01–2.86 (m, 3H), 2.78 (dt, *J* = 10.3, 3.5 Hz, 1H), 2.58 (t, *J* = 2.5 Hz, 1H), 2.27–2.14 (m, 4H), 2.05–0.60 (m, 36H) ppm; ^13^C NMR (101 MHz, CDCl_3_) *δ* 215.2, 171.1, 160.9, 143.7, 135.5, 124.2, 121.8, 110.6, 85.8, 85.0, 77.2, 77.0, 75.7, 74.0, 71.6, 70.5, 54.9, 52.6, 49.3, 39.4, 36.7, 35.2, 34.6, 34.3, 30.6, 30.0, 29.4, 21.0, 18.2, 16.0, 15.8, 14.1, 13.5, 12.7, 12.2, 8.4, 6.4 ppm; FT-IR (KBr tablet): 3454 (br, m), 3311 (m), 2965 (s), 2938 (s), 2879 (s), 2130 (w), 1714 (s), 1660 (s), 1616 (m), 1582 (m), 1458 (s), 1411 (s) cm^–1^; ESI MS (*m/z*) [M + Na]^+^ Calcd for C_37_H_56_NaO_8_ 651; Found 651.

#### 2.2.3. Synthesis of Propargyl Carbonate of Betulinic Acid (Compound **10**) 

To a stirred solution of **BET** (600 mg, 1.32 mmol, 1.0 equiv.) in anhydrous pyridine (15 mL) at room temperature, catalytic DMAP was added in one portion, followed by the addition of propargyl chloroformate (469 mg, 3.96 mmol, 3.0 equiv.) introduced dropwise over 30 seconds. The resulting solution was stirred at room temperature for the next 48 h. The reaction mixture was then concentrated under reduced pressure. Purification on silica gel using the CombiFlash system (0 → 30% EtOAc/*n*-hexane) gave the pure product of reaction **10** (15% yield) as a clear oil. The oil was diluted in *n*-pentane and evaporated to dryness three times to form an amorphous solid. The ^1^H and ^13^C NMR spectra of carbonate **10** are included in the Supplementary Materials (Figures S5 and S6).

Yield: 106 mg, 15%. Isolated as a white amorphous solid, >95% pure by NMR, and a single spot by TLC; R*_f_*: 0.72 in 33% EtOAc/*n*-hexane. Strains green with PMA; ^1^H NMR (400 MHz, CDCl_3_) *δ* ~12.00 (very broad s, 1H), 4.74 (d, *J* = 2.0 Hz, 1H), 4.71 (dd, *J* = 2.4, 1.8 Hz, 2H), 4.61 (dd, *J* = 2.1, 1.4 Hz, 1H), 4.33 (dd, *J* = 10.9, 5.1 Hz, 1H), 3.00 (td, *J* = 10.7, 4.7 Hz, 1H), 2.51 (t, *J* = 2.4 Hz, 1H), 2.27 (dt, *J* = 12.5, 2.8 Hz, 1H), 2.23–2.13 (m, 1H), 2.04–1.93 (m, 2H), 1.80–0.60 (m, 38H) ppm; ^13^C NMR (101 MHz, CDCl_3_) *δ* 181.9, 154.5, 150.3, 109.7, 86.3, 77.2, 75.4, 56.4, 55.4, 54.9, 50.4, 49.2, 46.9, 42.4, 40.7, 38.4, 38.3, 38.0, 37.1, 37.0, 34.2, 32.1, 30.5, 29.7, 27.8, 25.4, 23.6, 20.9, 19.3, 18.1, 16.3, 16.1, 16.0, 14.7 ppm; FT-IR (KBr tablet): 3700–2300 (very br, m), 3297 (m), 3073 (m), 2948 (s), 2871 (s), 2130 (w), 1747 (s), 1687 (s), 1642 (m), 1485 (m), 1465 (s), 1452 (s), 1439 (s) cm^–1^; ESI MS (*m/z*) [M + H]^+^ Calcd for C_34_H_50_NaO_5_ 539; Found 539.

#### 2.2.4. General Procedure for Preparation of Triazole-Linked Dimers (Compounds **14–22**)

Under a nitrogen atmosphere, to a solution of 20-*epi*-azidosalinomycin **13** (1.0 equiv.) in anhydrous CH_3_CN, the respective propargyl component **4**–**12** (1.0 equiv.) and DIPEA (3.0 equiv.) were introduced, followed by the addition of catalytic CuI (0.1 equiv.) in one portion. The reaction mixture was stirred at room temperature, typically for 24 h. After the consumption of the azide and propargyl partners (TLC control), the organic solvent was removed on a rotary evaporator. The oily residue was then dissolved in a small amount of CH_2_Cl_2_ or CHCl_3_ and extracted a few times with 10% aq. EDTA solution. Organic phases were separated, and concentrated under reduced pressure. Purification on silica gel using the CombiFlash system (0 → 100% EtOAc/*n*-hexane for **14**, **17** and **20**–**22**; 0 → 50% EtOAc/*n*-hexane for **15**; 0 → 100% acetone/CHCl_3_ for **16**; and 0 → 5% MeOH/CHCl_3_ for **18**–**19**) gave the pure products of the ‘click’ reactions **14**–**22** (37–81% yield) as clear oils. The oils were diluted in *n*-pentane and evaporated to dryness three times to form the amorphous solids. The ^1^H and ^13^C NMR spectra of dimers **14**–**22** are included in the Supplementary Materials (Figures S7–S24). 

*Salinomycin-lasalocid acid dimer ***14****: Yield: 140 mg, 70%. Isolated as a white amorphous solid, >95% pure by NMR, and a single spot by TLC; R*_f_*: 0.82 in 100% EtOAc. UV-active and strains green with PMA; ^1^H NMR (400 MHz, CDCl_3_) δ 9.53 (s, 1H), 7.82 (s, 1H), 7.28–7.19 (m, 1H), 7.04 (dd, *J* = 7.8, 0.5 Hz, 1H), 6.66 (d, *J* = 7.7 Hz, 1H), 6.55 (dd, *J* = 10.8, 1.6 Hz, 1H), 6.13 (dd, *J* = 10.7, 4.8 Hz, 1H), 5.36 (dd, *J* = 4.8, 1.6 Hz, 1H), 5.18 (s, *J* = 38.4 Hz, 2H), 4.78 (dd, *J* = 15.6, 6.2 Hz, 1H), 4.56 (dd, *J* = 15.6, 5.4 Hz, 1H), 4.33 (dd, *J* = 13.4, 6.5 Hz, 1H), 4.20 (dd, *J* = 10.2, 0.9 Hz, 2H), 4.02–3.91 (m, 2H), 3.88 (dd, *J* = 10.1, 4.2 Hz, 1H), 3.78 (s, 1H), 3.74–3.61 (m, 2H), 3.46–3.33 (m, 2H), 2.92 (ddd, *J* = 14.3, 9.2, 7.0 Hz, 1H), 2.84–2.76 (m, 1H), 2.76–2.59 (m, 4H), 2.21 (s, *J* = 16.2 Hz, 3H), 2.10–0.50 (m, 93H) ppm; ^1^^3^C NMR (101 MHz, CDCl_3_) δ 217.6, 213.4, 184.5, 170.1, 154.5, 144.5, 138.4, 132.1, 126.8, 123.1, 122.7, 120.7, 120.4, 116.4, 107.3, 98.3, 89.4, 86.2, 84.5, 75.9, 75.7, 75.4, 74.4, 74.1, 71.2, 71.1, 70.4, 70.2, 67.2, 57.7, 55.3, 54.5, 51.0, 50.0, 48.5, 40.1, 38.6, 38.3, 37.0, 36.2, 35.7, 35.6, 34.8, 33.4, 32.4, 32.2, 32.1, 31.2, 30.9, 30.5, 29.6, 29.52, 29.46, 29.2, 27.8, 27.5, 26.8, 23.4, 21.0, 20.6, 19.8, 17.4, 17.2, 16.9, 15.8, 15.6, 14.5, 14.0, 13.4, 12.9, 12.57, 12.55, 11.9, 11.4, 10.5, 8.6, 6.7, 6.3, 6.1 ppm; FT-IR (KBr tablet): 3439 (br, s), 2964 (s), 2937 (s), 2877 (s), 1713 (s), 1651 (m), 1632 (m), 1614 (m), 1565 (s), 1522 (m), 1459 (s), 1406 (s) cm^–1^; ESI MS (*m/z*) [M + H]^+^ Calcd for C_79_H_126_N_4_NaO_17_ 1426; Found 1426; [M + Na]^+^ Calcd for C_79_H_125_N_4_Na_2_O_17_^+^ 1448; Found 1448; [M + H + Na]^2+^ Calcd for C_79_H_126_N_4_Na_2_O_17_ 725; Found 725.

*Salinomycin-lasalocid acid dimer ***15****: Yield: 170 mg, 70%. Isolated as a white amorphous solid, >95% pure by NMR, and a single spot by TLC; R*_f_*: 0.83 in 50% EtOAc/*n*-hexane. UV-active and strains green with PMA; ^1^H NMR (400 MHz, CDCl_3_) *δ* 11.17 (s, 1H), 7.68 (s, 1H), 7.07 (dd, *J* = 7.7, 0.5 Hz, 1H), 6.57 (d, *J* = 7.6 Hz, 1H), 6.53 (dd, *J* = 10.7, 1.3 Hz, 1H), 6.10 (dd, *J* = 10.7, 5.0 Hz, 1H), 5.41 (dd, *J* = 26.1, 12.6 Hz, 2H), 5.28 (dd, *J* = 5.0, 1.3 Hz, 1H), 5.09 (s, 2H), 4.25 (dd, *J* = 13.4, 6.6 Hz, 1H), 4.14 (dd, *J* = 10.4, 0.6 Hz, 1H), 3.90–3.80 (m, 2H), 3.77– 3.65 (m, 2H), 3.64–3.55 (m, 2H), 3.39 (dd, *J* = 11.4, 2.0 Hz, 1H), 3.35–3.26 (m, 2H), 2.93–2.67 (m, 5H), 2.67–2.54 (m, 2H), 2.12 (s, 3H), 2.05–0.50 (m, 92H) ppm; ^13^C NMR (101 MHz, CDCl_3_) *δ* 217.5, 215.3, 184.4, 171.5, 160.7, 143.7, 141.6, 135.2, 127.7, 124.0, 123.9, 122.9, 121.7, 116.3, 110.9, 107.1, 98.5, 89.9, 85.5, 84.9, 76.3, 75.7, 75.6, 74.4, 73.9, 71.7, 71.3, 70.4, 69.7, 67.3, 58.2, 57.4, 55.4, 55.0, 51.0, 50.0, 49.1, 40.3, 39.4, 38.4, 36.5, 36.4, 35.8, 35.4, 34.4, 34.1, 32.4, 32.3, 32.2, 30.5, 29.9, 29.61, 29.56, 29.2, 29.0, 27.9, 27.6, 26.9, 23.7, 21.0, 19.9, 18.3, 17.5, 17.2, 16.0, 15.8, 15.6, 14.5, 14.0, 13.5, 13.0, 12.6, 12.4, 12.3, 11.9, 10.6, 8.4, 6.7, 6.4 ppm; FT-IR (KBr tablet): 3419 (br, s), 2964 (s), 2936 (s), 2877 (s), 1714 (s), 1656 (m), 1617 (m), 1565 (s), 1459 (s), 1408 (s) cm^–1^; ESI MS (*m/z*) [M + H]^+^ Calcd for C_79_H_125_N_3_NaO_18_ 1427; Found 1427; [M + Na]^+^ Calcd for C_79_H_124_N_3_Na_2_O_18_^+^ 1449; Found 1449; [M + H + Na]^2+^ Calcd for C_79_H_125_N_3_Na_2_O_18_ 725; Found 725.

*Salinomycin-monensin dimer ***16***:* Yield: 170 mg, 63%. Isolated as a white amorphous solid, >95% pure by NMR, and a single spot by TLC; R*_f_*: 0.38 in 50% acetone/CHCl_3_. Strains green with PMA; ^1^H NMR (400 MHz, CDCl_3_) *δ* 7.46 (s, 1H), 7.15–7.06 (m, 1H), 6.49 (d, *J* = 10.7 Hz, 1H), 6.07 (dd, *J* = 10.7, 5.0 Hz, 1H), 5.14 (d, *J* = 4.9 Hz, 1H), 4.95 (s, 1H), 4.59 (d, *J* = 6.8 Hz, 1H), 4.47 (dd, *J* = 14.6, 5.2 Hz, 1H), 4.33–4.20 (m, 2H), 4.14 (dd, *J* = 10.2, 4.9 Hz, 1H), 4.03 (d, *J* = 8.8 Hz, 1H), 3.82 (ddd, *J* = 12.9, 11.6, 3.7 Hz, 2H), 3.75–3.67 (m, 1H), 3.64–3.57 (m, 1H), 3.51–3.43 (m, 1H), 3.42–3.23 (m, 4H), 2.86 (s, 1H), 2.77–2.68 (m, 1H), 2.66–2.57 (m, 1H), 2.41 (td, *J* = 10.6, 6.6 Hz, 1H), 2.30–0.50 (m, 108H) ppm; ^13^C NMR (101 MHz, CDCl_3_) *δ* 217.5, 184.3, 175.7, 144.7, 127.4, 123.0, 121.5, 116.3, 107.6, 107.2, 98.5, 97.2, 89.9, 85.9, 85.4, 83.1, 81.7, 77.2, 76.5, 75.7, 75.5, 75.2, 74.4, 71.3, 71.0, 70.9, 69.6, 67.8, 67.2, 67.1, 58.3, 57.3, 51.0, 50.0, 41.8, 40.4, 38.8, 38.4, 37.0, 36.5, 36.3, 36.2, 35.8, 34.9, 34.7, 34.2, 33.3, 32.7, 32.6, 32.40, 32.35, 32.2, 31.7, 30.9, 30.0, 29.6, 29.1, 27.9, 27.6, 26.9, 23.6, 22.6, 20.9, 19.9, 17.5, 17.4, 17.2, 16.2, 15.6, 15.4, 14.5, 13.4, 13.0, 12.5, 12.4, 11.9, 10.8, 10.6, 8.1, 6.7, 6.4 ppm; FT-IR (KBr tablet): 3369 (br, s), 2966 (s), 2933 (s), 2879 (s), 1714 (s), 1663 (s), 1565 (s), 1512 (m), 1460 (s), 1405 (s), cm^–1^; ESI MS (*m/z*) [M + H]^+^ Calcd for C_81_H_134_N_4_NaO_20_ 1506; Found 1506; [M + H + Na]^2+^ Calcd for C_81_H_134_N_4_Na_2_O_20_ 765; Found 765.

*Salinomycin-monensin dimer ***17***:* Yield: 140 mg, 52%. Isolated as a white amorphous solid, >95% pure by NMR, and a single spot by TLC; R*_f_*: 0.40 in 100% EtOAc. Strains green with PMA; ^1^H NMR (400 MHz, CDCl_3_) *δ* 7.63 (s, 1H), 6.57 (d, *J* = 10.7 Hz, 1H), 6.14 (dd, *J* = 10.7, 5.1 Hz, 1H), 5.32 (dd, *J* = 5.0, 1.3 Hz, 1H), 5.24 (d, *J* = 12.8 Hz, 1H), 5.16 (d, *J* = 12.8 Hz, 1H), 4.34–4.18 (m, 4H), 4.01 (dd, *J* = 9.3, 2.0 Hz, 1H), 3.90 (dd, *J* = 11.1, 4.6 Hz, 1H), 3.82 (t, *J* = 4.7 Hz, 2H), 3.79–3.72 (m, 2H), 3.68–3.63 (m, 2H), 3.59 (dd, *J* = 9.7, 5.7 Hz, 1H), 3.53 (t, *J* = 4.7 Hz, 1H), 3.45 (s, 2H), 3.38 (d, *J* = 11.2 Hz, 1H), 3.27– 3.16 (m, 4H), 2.78 (td, *J* = 11.0, 3.4 Hz, 1H), 2.72–2.57 (m, 4H), 2.28–2.22 (m, 1H), 2.20–0.50 (m, 99H) ppm; ^13^C NMR (101 MHz, CDCl_3_) *δ* 217.5, 184.4, 175.0, 142.4, 127.6, 123.4, 123.0, 107.5, 107.2, 98.5, 96.9, 90.0, 87.3, 86.2, 85.6, 83.5, 81.5, 76.5, 76.3, 76.2, 75.7, 74.4, 71.3, 71.2, 69.8, 67.9, 67.4, 67.3, 58.2, 57.8, 57.4, 55.4, 51.1, 50.1, 41.0, 40.4, 39.1, 38.5, 36.9, 36.7, 36.4, 36.1, 35.8, 35.1, 34.7, 34.4, 33.6, 32.8, 32.5, 32.4, 32.2, 32.1, 31.0, 29.6, 29.5, 29.0, 28.0, 27.8, 27.6, 26.9, 25.6, 23.7, 21.0, 20.0, 17.5, 17.29, 17.25, 16.1, 15.7, 15.6, 14.6, 13.1, 12.4, 12.3, 12.0, 11.7, 11.1, 10.7, 8.0, 6.7, 6.4 ppm; FT-IR (KBr tablet): 3439 (br, s), 2965 (s), 2935 (s), 2878 (s), 1736 (s), 1715 (s), 1636 (m), 1565 (s), 1460 (s), 1405 (s) cm^–1^; ESI MS (*m/z*) [M + H]^+^ Calcd for C_81_H_133_N_3_NaO_21_ 1507; Found 1507; [M + Na]^+^ Calcd for C_81_H_132_N_3_Na_2_O_21_ 1529; Found 1529; [M + H + Na]^2+^ Calcd for C_81_H_133_N_3_Na_2_O_21_ 766; Found 766.

*Salinomycin-betulinic acid dimer ***18***:* Yield: 130 mg, 56%. Isolated as a white amorphous solid, >95% pure by NMR, and a single spot by TLC; R*_f_*: 0.19 in 5% MeOH/CHCl_3_. Strains green with PMA; ^1^H NMR (400 MHz, CDCl_3_) *δ* 7.48 (s, 1H), 6.50 (dd, *J* = 10.7, 1.5 Hz, 1H), 6.23 (t, *J* = 5.5 Hz, 1H), 6.07 (dd, *J* = 10.7, 5.0 Hz, 1H), 5.29 (dd, *J* = 5.0, 1.4 Hz, 1H), 5.05 (s, 1H), 4.65 (d, *J* = 2.1 Hz, 1H), 4.51 (dd, *J* = 2.2, 1.3 Hz, 1H), 4.40 (dd, *J* = 15.2, 5.8 Hz, 1H), 4.31 (dd, *J* = 15.2, 5.2 Hz, 1H), 4.26 (dd, *J* = 13.5, 6.6 Hz, 1H), 4.15 (d, *J* = 10.3 Hz, 1H), 3.85 (dd, *J* = 11.0, 5.0 Hz, 1H), 3.64–3.55 (m, 2H), 3.33 (dd, *J* = 12.0, 1.8 Hz, 1H), 3.11 (dd, *J* = 11.0, 4.7 Hz, 1H), 3.04 (td, *J* = 11.1, 4.2 Hz, 1H), 2.73 (td, *J* = 10.8, 3.3 Hz, 1H), 2.66–2.55 (m, 2H), 2.40 (s, 1H), 2.34–2.22 (m, 2H), 2.10–0.50 (m, 95H) ppm; ^13^C NMR (101 MHz, CDCl_3_) *δ* 217.4, 184.6, 176.1, 150.9, 144.4, 127.5, 123.0, 121.5, 109.3, 107.2, 98.5, 89.9, 78.9, 76.3, 75.69, 75.66, 74.5, 71.3, 69.8, 67.3, 57.5, 55.6, 55.34, 55.27, 51.1, 50.5, 50.0, 46.7, 42.4, 40.6, 40.3, 38.8, 38.6, 38.5, 38.1, 37.7, 37.1, 36.4, 35.9, 34.7, 34.2, 33.4, 32.5, 32.4, 32.3, 30.8, 29.7, 29.4, 29.0, 27.94, 27.91, 27.7, 27.4, 26.9, 25.5, 23.7, 21.0, 20.8, 19.9, 19.4, 18.2, 17.5, 17.4, 16.13, 16.09, 15.7, 15.3, 14.59, 14.56, 13.1, 12.4, 12.0, 10.6, 6.7, 6.4 ppm; FT-IR (KBr tablet): 3392 (br, s), 3071 (m), 2962 (s), 2938 (s), 2873 (s), 1715 (s), 1660 (s), 1642 (s), 1565 (s), 1509 (m), 1459 (s), 1405 (s) cm^–1^; ESI MS (*m/z*) [M + H]^+^ Calcd for C_75_H_120_N_4_NaO_12_ 1292; Found 1292.

*Salinomycin-betulinic acid dimer ***19***:* Yield: 200 mg, 81%. Isolated as a white amorphous solid, >95% pure by NMR, and a single spot by TLC; R*_f_*: 0.31 in 5% MeOH/CHCl_3_. Strains green with PMA; ^1^H NMR (400 MHz, CDCl_3_) *δ* 7.58 (s, 1H), 6.52 (dd, *J* = 10.7, 1.4 Hz, 1H), 6.10 (dd, *J* = 10.7, 5.0 Hz, 1H), 5.29 (dd, *J* = 5.1, 1.4 Hz, 1H), 5.09 (dd, *J* = 25.8, 12.7 Hz, 2H), 4.63 (d, *J* = 2.0 Hz, 1H), 4.51 (dd, *J* = 2.1, 1.4 Hz, 1H), 4.26 (dd, *J* = 13.5, 6.7 Hz, 1H), 4.15 (d, *J* = 10.3 Hz, 1H), 3.86 (dd, *J* = 11.0, 5.0 Hz, 1H), 3.66–3.54 (m, 2H), 3.33 (dd, *J* = 12.0, 1.8 Hz, 1H), 3.11 (dd, *J* = 11.0, 4.7 Hz, 1H), 2.92–2.83 (m, 1H), 2.73 (td, *J* = 10.8, 3.2 Hz, 1H), 2.67–2.55 (m, 2H), 2.40–0.50 (m, 99H) ppm; ^13^C NMR (101 MHz, CDCl_3_) *δ* 217.4, 184.5, 175.7, 150.4, 142.5, 127.5, 123.2, 123.0, 109.6, 107.1, 98.5, 89.9, 78.8, 76.3, 75.7, 75.6, 74.4, 71.3, 69.8, 67.3, 57.4, 57.2, 56.4, 55.32, 55.25, 51.1, 50.4, 50.0, 49.3, 46.8, 42.3, 40.6, 40.3, 38.8, 38.6, 38.5, 38.2, 37.1, 36.8, 36.4, 35.8, 34.2, 32.44, 32.35, 32.2, 31.8, 30.5, 29.5, 29.0, 27.92, 27.89, 27.6, 27.3, 26.9, 25.4, 23.7, 21.0, 20.8, 19.9, 19.3, 18.2, 17.5, 17.3, 16.1, 15.9, 15.6, 15.3, 14.59, 14.56, 13.0, 12.4, 12.0, 10.6, 6.7, 6.4 ppm; FT-IR (KBr tablet): 3412 (br, s), 3073 (m), 2961 (s), 2939 (s), 2873 (s), 1717 (s), 1642 (m), 1565 (s), 1459 (s), 1405 (s) cm^–1^; ESI MS (*m/z*) [M + H]^+^ Calcd for C_75_H_119_N_3_NaO_13_ 1293; Found 1293; [M + Na]^+^ Calcd for C_75_H_118_N_3_Na_2_O_13_ 1315; Found 1315.

*Salinomycin-betulinic acid dimer ***20***:* Yield: 45 mg, 37%. Isolated as a white amorphous solid, >95% pure by NMR, and a single spot by TLC; R*_f_*: 0.70 in 100% EtOAc. Strains green with PMA; ^1^H NMR (400 MHz, CDCl_3_) *δ*~12.00 (very broad s, 1H), 7.67 (s, 1H), 6.57 (dd, *J* = 10.7, 1.0 Hz, 1H), 6.16 (dd, *J* = 10.7, 4.9 Hz, 1H), 5.35–5.31 (m, 1H), 5.23–5.15 (m, 2H), 4.71 (d, *J* = 1.6 Hz, 1H), 4.59 (dd, *J* = 1.8, 1.3 Hz, 1H), 4.33–4.25 (m, 2H), 4.22 (dd, *J* = 10.5, 0.7 Hz, 1H), 3.93 (dd, *J* = 10.9, 4.7 Hz, 1H), 3.76–3.72 (m, 2H), 3.67 (t, *J* = 9.6 Hz, 2H), 3.39 (dd, *J* = 11.9, 1.9 Hz, 1H), 2.98 (dq, *J* = 8.1, 4.6 Hz, 1H), 2.86–2.78 (m, 1H), 2.74–2.55 (m, 2H), 2.50–0.50 (m, 96H) ppm; ^13^C NMR (101 MHz, CDCl_3_) *δ* 217.4, 184.5, 179.8, 155.0, 150.6, 141.8, 127.6, 123.5, 123.1, 109.6, 107.2, 98.6, 89.9, 85.7, 76.4, 75.7, 74.5, 71.3, 69.8, 67.9, 67.6, 60.6, 57.5, 56.2, 55.43, 55.40, 50.9, 50.4, 50.0, 49.2, 46.9, 42.4, 40.7, 40.4, 38.5, 38.3, 38.2, 38.0, 37.1, 36.5, 35.8, 34.3, 32.5, 32.4, 32.2, 30.9, 30.6, 29.7, 29.6, 29.0, 28.0, 27.8, 27.7, 26.9, 25.5, 23.7, 23.6, 21.0, 20.9, 20.0, 19.3, 18.1, 17.5, 17.2, 16.3, 16.11, 16.08, 15.7, 14.63, 14.57, 13.1, 12.4, 12.0, 10.7, 6.7, 6.4 ppm; FT-IR (KBr tablet): 3412 (br, m), 3071 (m), 2961 (s), 2939 (s), 2875 (s), 1743 (s), 1716 (s), 1642 (m), 1565 (s), 1459 (s), 1403 (s) cm^–1^; ESI MS (*m/z*) [M + H]^+^ Calcd for C_76_H_119_N_3_NaO_15_ 1337; Found 1337.

*Salinomycin-salinomycin dimer ***21***:* Yield: 120 mg, 63%. Isolated as a white amorphous solid, >95% pure by NMR, and a single spot by TLC; R*_f_*: 0.62 in 100% EtOAc. Strains green with PMA; ^1^H NMR (400 MHz, CDCl_3_) *δ* 7.60 (s, 1H), 6.97 (t, *J* = 5.1 Hz, 1H), 6.53 (dd, *J* = 10.6, 1.0 Hz, 1H), 6.10 (dd, *J* = 10.2, 4.5 Hz, 1H), 6.08–6.04 (m, 1H), 5.96 (d, *J* = 11.0 Hz, 1H), 5.30 (dd, *J* = 5.0, 0.8 Hz, 1H), 4.72 (dd, *J* = 15.3, 5.5 Hz, 1H), 4.58 (dd, *J* = 15.1, 5.0 Hz, 1H), 4.29 (q, *J* = 6.6 Hz, 1H), 4.18 (dd, *J* = 14.7, 10.1 Hz, 2H), 4.03–3.85 (m, 5H), 3.80 (q, *J* = 7.0 Hz, 1H), 3.71–3.57 (m, 6H), 3.36 (dd, *J* = 11.9, 1.3 Hz, 1H), 3.10–3.00 (m, 1H), 2.78 (td, *J* = 11.0, 3.2 Hz, 1H), 2.72–2.58 (m, 4H), 2.35 (dt, *J* = 12.5, 9.7 Hz, 1H), 2.20–2.12 (m, 1H), 2.10–0.50 (m, 108H) ppm; ^13^C NMR (101 MHz, CDCl_3_) *δ* 217.4, 213.4, 184.4, 175.0, 144.9, 133.1, 127.3, 123.0, 121.9, 120.6, 107.4, 106.3, 98.8, 98.5, 89.9, 88.3, 79.9, 76.8, 76.3, 75.7, 75.6, 75.1, 74.4, 74.3, 71.34, 71.27, 70.8, 69.7, 69.3, 67.4, 67.3, 57.4, 55.42, 55.38, 51.1, 50.1, 48.2, 47.0, 40.4, 40.2, 38.7, 38.5, 36.62, 36.57, 36.3, 35.8, 35.0, 33.0, 32.5, 32.4, 32.2, 30.6, 30.3, 29.0, 29.0, 28.2, 28.0, 27.6, 26.9, 26.5, 25.9, 23.6, 22.4, 21.9, 21.0, 20.3, 20.0, 18.8, 17.50, 17.46, 17.3, 15.7, 15.6, 14.58, 14.55, 14.5, 13.5, 13.1, 12.4, 12.0, 11.7, 11.3, 10.7, 8.0, 6.7, 6.4, 6.3 ppm; FT-IR (KBr tablet): 3416 (br, s), 2964 (s), 2936 (s), 2876 (s), 1714 (s), 1656 (s), 1565 (s), 1529 (m), 1460 (s), 1404 (s) cm^–1^; ESI MS (*m/z*) [M + H]^+^ Calcd for C_87_H_142_N_4_NaO_20_ 1586; Found 1586; [M + H + Na]^2+^ Calcd for C_87_H_142_N_4_Na_2_O_20_ 805; Found 805.

*Salinomycin-salinomycin dimer ***22***:* Yield: 100 mg, 52%. Isolated as a white amorphous solid, >95% pure by NMR, and a single spot by TLC; R*_f_*: 0.75 in 100% EtOAc. Strains green with PMA; ^1^H NMR (400 MHz, CDCl_3_) *δ* 7.66 (s, 1H), 6.56 (dd, *J* = 10.8, 1.0 Hz, 1H), 6.15 (dd, *J* = 10.7, 5.0 Hz, 1H), 6.06 (dd, *J* = 10.8, 2.1 Hz, 1H), 5.96 (dd, *J* = 10.8, 1.0 Hz, 1H), 5.50 (d, *J* = 12.8 Hz, 1H), 5.33 (dd, *J* = 5.1, 1.0 Hz, 1H), 5.25 (d, *J* = 12.8 Hz, 1H), 4.33–4.27 (m, 1H), 4.20 (d, *J* = 10.3 Hz, 1H), 4.06 (dd, *J* = 10.1, 3.7 Hz, 1H), 4.00 (dd, *J* = 10.2, 6.0 Hz, 3H), 3.90 (dd, *J* = 10.3, 4.9 Hz, 3H), 3.81 (q, *J* = 6.8 Hz, 1H), 3.71–3.58 (m, 6H), 3.55 (dd, *J* = 10.5, 2.7 Hz, 1H), 3.37 (dd, *J* = 12.1, 1.3 Hz, 1H), 3.20–3.11 (m, 1H), 3.08 (d, *J* = 5.0 Hz, 1H), 2.96 (td, *J* = 10.8, 4.0 Hz, 1H), 2.78 (td, *J* = 10.9, 3.2 Hz, 1H), 2.73–2.60 (m, 4H), 2.37 (dt, *J* = 12.5, 9.5 Hz, 1H), 2.23–2.15 (m, 1H), 2.10–0.50 (m, 104H) ppm; ^13^C NMR (101 MHz, CDCl_3_) *δ* 217.5, 213.9, 184.4, 175.2, 142.3, 132.8, 127.5, 123.8, 123.0, 120.9, 107.3, 106.3, 99.0, 98.5, 90.0, 87.7, 80.0, 76.9, 76.4, 75.7, 75.6, 74.7, 74.4, 74.1, 71.7, 71.3, 70.9, 69.7, 69.2, 67.6, 67.4, 58.1, 57.4, 57.2, 55.4, 51.1, 50.1, 48.4, 47.8, 40.4, 40.3, 39.0, 38.5, 36.5, 36.41, 36.37, 35.8, 33.4, 32.5, 32.4, 32.2, 30.6, 30.3, 29.6, 29.04, 29.00, 28.02, 27.96, 27.6, 26.9, 26.2, 25.7, 23.7, 22.5, 22.1, 21.0, 20.4, 20.0, 19.6, 17.5, 17.3, 15.7, 15.5, 14.6, 14.5, 14.0, 13.1, 12.7, 12.4, 12.0, 11.6, 10.9, 10.7, 7.3, 6.7, 6.4, 6.3 ppm; FT-IR (KBr tablet): 3519 (br, s), 3435 (br, s), 2965 (s), 2936 (s), 2876 (s), 1736 (s), 1715 (s), 1635 (m), 1565 (s), 1460 (s), 1404 (s) cm^–1^; ESI MS (*m/z*) [M + H]^+^ Calcd for C_87_H_141_N_3_NaO_21_ 1587; Found 1587; [M + H + Na]^2+^ Calcd for C_87_H_141_N_3_Na_2_O_21_ 805; Found 805.

#### 2.2.5. General Procedure for Preparation of Dimeric Conjugates with Hydroxamic Acids (Compounds **23–24**)

Initially, dimer **22** in the sodium salt form needed to be quantitatively transformed to its acid form; for this purpose, compound **22** was dissolved in CH_2_Cl_2_ and extracted twice with an aqueous solution of H_2_SO_4_ (pH = 1.0), followed by extraction with water. The organic layers were collected, and evaporated to dryness under reduced pressure. Then, to a stirred solution of dimer **22** (1.0 equiv.) in anhydrous CH_2_Cl_2_ at room temperature, DCC (2.0 equiv.) and DMAP (excess) were introduced, followed by the addition of respective hydroxamic acid (5.0 equiv.) in one portion. The resulting solution was stirred overnight, then concentrated under reduced pressure. Purification on silica gel using the CombiFlash system (0 → 50% EtOAc/*n*-hexane) gave the pure products of reaction **23** and **24** (31–32% yield) as clear oils. The oils were diluted in *n*-pentane and evaporated to dryness three times to form the amorphous solids. The ^1^H and ^13^C NMR spectra of dimers **23** and **24** are included in the Supplementary Materials (Figures S25–S28).

*Salinomycin-salinomycin dimer ***23***:* Yield: 28 mg, 32%. Isolated as a white amorphous solid, >95% pure by NMR, and a single spot by TLC; R*_f_*: 0.27 in 50% EtOAc/*n*-hexane. Strains green with PMA; ^1^H NMR (400 MHz, CDCl_3_) *δ* 11.10 (s, 1H), 8.17–8.08 (m, 2H), 7.55 (s, 1H), 7.46–7.41 (m, 1H), 7.38–7.30 (m, 2H), 6.55 (dd, *J* = 10.6, 1.5 Hz, 1H), 6.05–5.95 (m, 2H), 5.91 (dd, *J* = 10.6, 0.8 Hz, 1H), 5.43 (d, *J* = 12.8 Hz, 1H), 5.24–5.17 (m, 2H), 4.07 (dd, *J* = 11.1, 6.4 Hz, 1H), 4.01 (d, *J* = 9.4 Hz, 2H), 3.94 (dd, *J* = 10.4, 5.6 Hz, 2H), 3.86–3.70 (m, 4H), 3.64–3.55 (m, 2H), 3.49 (dd, *J* = 10.4, 2.5 Hz, 1H), 3.43 (dd, *J* = 11.6, 1.9 Hz, 1H), 3.18–3.08 (m, 2H), 2.96–2.90 (m, 1H), 2.90–2.84 (m, 1H), 2.84–2.78 (m, 1H), 2.67–2.61 (m, 1H), 2.54–2.45 (m, 2H), 2.37–2.26 (m, 2H), 2.25–0.50 (m, 109H) ppm; ^13^C NMR (101 MHz, CDCl_3_) *δ* 215.5, 214.0, 175.3, 173.5, 165.1, 142.9, 132.8, 131.9, 131.0, 129.5, 128.8, 128.2, 128.0, 123.4, 120.8, 107.2, 106.3, 99.0, 97.9, 90.7, 87.7, 80.0, 79.7, 77.2, 76.9, 76.2, 74.7, 74.1, 73.6, 72.2, 71.8, 71.0, 70.9, 69.1, 68.3, 67.9, 67.5, 58.4, 57.2, 48.5, 47.9, 46.0, 45.6, 41.3, 40.3, 39.7, 39.1, 38.1, 37.1, 36.5, 36.4, 36.3, 33.4, 32.4, 31.6, 30.8, 30.6, 30.3, 29.7, 29.0, 28.0, 26.9, 26.5, 26.1, 25.7, 25.2, 22.62, 22.58, 22.2, 22.0, 20.4, 19.6, 18.7, 17.5, 16.94, 16.85, 15.6, 15.4, 14.7, 14.5, 14.3, 14.1, 14.0, 12.8, 11.8, 11.6, 11.4, 10.9, 7.8, 7.3, 6.7, 6.3 ppm; FT-IR (KBr tablet): 3514 (br, s), 3444 (br, s), 3333 (br, m), 2964 (s), 2933 (s), 2876 (s), 1787 (m), 1733 (s), 1716 (s), 1696 (s), 1631 (w), 1603 (w), 1583 (w), 1502 (w), 1461 (s) cm^–1^; ESI MS (*m/z*) [M + H]^+^ Calcd for C_94_H_147_N_4_O_22_ 1685; Found 1685; [M + Na]^+^ Calcd for C_94_H_146_N_4_NaO_22_ 1707; Found 1707; [M + 2Na]^2+^ Calcd for C_94_H_146_N_4_Na_2_O_22_ 865; Found 865.

*Salinomycin-salinomycin dimer ***24***:* Yield: 27 mg, 31%. Isolated as a white amorphous solid, >95% pure by NMR, and a single spot by TLC; R*_f_*: 0.38 in 50% EtOAc/*n*-hexane. Strains green with PMA; ^1^H NMR (400 MHz, CDCl_3_) *δ* 11.80 (s, 1H), 11.31 (s, 1H), 8.30 (dd, *J* = 8.0, 1.4 Hz, 1H), 7.62 (s, 1H), 7.43–7.36 (m, 1H), 6.97 (dd, *J* = 8.4, 1.0 Hz, 1H), 6.81 (ddd, *J* = 8.2, 7.3, 1.1 Hz, 1H), 6.64 (dd, *J* = 10.7, 1.5 Hz, 1H), 6.07 (ddd, *J* = 10.7, 5.1, 3.4 Hz, 2H), 5.98 (dd, *J* = 10.8, 0.9 Hz, 1H), 5.50 (d, *J* = 12.9 Hz, 1H), 5.31–5.23 (m, 2H), 4.14 (dd, *J* = 11.3, 6.3 Hz, 1H), 4.08 (d, *J* = 9.6 Hz, 1H), 4.01 (dd, *J* = 10.7, 4.8 Hz, 2H), 3.96–3.77 (m, 4H), 3.71–3.62 (m, 2H), 3.56 (dd, *J* = 10.5, 2.3 Hz, 1H), 3.49 (dd, *J* = 12.2, 1.9 Hz, 1H), 3.27–3.14 (m, 2H), 3.04–2.96 (m, 1H), 2.92 (ddd, *J* = 16.1, 8.6, 6.7 Hz, 1H), 2.74–2.68 (m, 1H), 2.64–2.60 (m, 1H), 2.51 (s, 1H), 2.47–2.31 (m, 2H), 2.30–0.50 (m, 111H) ppm; ^13^C NMR (101 MHz, CDCl_3_) *δ* 216.0, 214.0, 175.3, 173.4, 168.2, 161.6, 142.9, 134.6, 132.8, 129.5, 128.8, 127.4, 126.2, 123.4, 120.8, 118.3, 118.0, 111.7, 107.2, 106.3, 99.0, 97.9, 90.9, 87.7, 80.0, 79.7, 77.2, 76.9, 76.2, 74.7, 74.1, 73.6, 72.1, 71.8, 70.93, 70.88, 69.1, 68.2, 67.9, 67.5, 58.3, 57.2, 48.5, 47.9, 46.0, 45.4, 41.3, 40.3, 39.7, 39.1, 38.0, 37.2, 36.5, 36.3, 34.6, 33.4, 32.4, 31.9, 31.6, 30.7, 30.6, 30.3, 29.7, 29.0, 28.0, 26.9, 25.8, 25.2, 23.2, 22.63, 22.59, 22.2, 20.4, 19.4, 17.6, 17.0, 16.9, 15.6, 14.8, 14.59, 14.55, 14.5, 14.3, 14.1, 14.0, 12.8, 11.7, 11.6, 11.4, 10.9, 7.8, 7.3, 6.7, 6.3 ppm; FT-IR (KBr tablet): 3516 (br, s), 3448 (br, s), 3317 (br, m), 2964 (s), 2933 (s), 2876 (s), 1790 (m), 1732 (s), 1716 (s), 1701 (s), 1655 (s), 1610 (m), 1588 (w), 1508 (m), 1460 (s) cm^–1^; ESI MS (*m/z*) [M + Na]^+^ Calcd for C_94_H_146_N_4_NaO_23_ 1723; Found 1723; [M + 2Na]^2+^ Calcd for C_94_H_146_N_4_Na_2_O_23_ 873; Found 873.

### 2.3. In Vitro Biological Studies

#### 2.3.1. Cell Lines and Culture Conditions

The in vitro antiproliferative activity of the tested compounds was evaluated on the biphenotypic B myelomonocytic leukemia cell line MV-4-11, breast cancer cell lines JIMT-1, MCF-7 and SKBR-3, colon cancer cell line LoVo and its doxorubicin-resistant sub-line LoVo/DX, and two non-cancerous cell lines: murine fibroblasts BALB/3T3 and human mammary epithelial cells MCF10A. JIMT-1 was purchased at DSMZ-German Collection of Microorganisms and Cell Cultures GmbH (Braunschweig, Germany), MCF-7 from European Collection of Authenticated Cell Culture (Salisbury, UK), MV-4-11, SKBR-3, LoVo, BALB/3T3, and MCF10A from the American Type Culture Collection (Manassas VA, USA), while LoVo/DX was a kind gift from Professor E. Borowski (Medical University of Gdańsk, Poland). 

JIMT-1 and BALB/3T3 were cultured in DMEM (Gibco, Paisley, UK) supplemented with 10% FBS (GE Healthcare, Logan UT, USA), 2 mM L-glutamine (Sigma-Aldrich, Steinheim, Germany). MCF-7 and SKBR-3 were cultured in EMEM (PChO IIET, Wrocław, Poland) supplemented with 10% FBS, 2 mM L-glutamine, 8 µg mL^–1^ insulin, 1% amino acid solution (Sigma-Aldrich, Steinheim, Germany). MCF10A were cultured in Ham’s F12 medium (Gibco, Scotland, UK), 5% horse serum (Gibco, New Zealand), 10 µg mL^–1^ insulin, 20 ng mL^–1^ rhEGF, 0.5 µg mL^–1^ hydrocortisone, as well as 0.5 µg mL^–1^ cholera toxin (Sigma-Aldrich, Steinheim, Germany). LoVo was cultured in F-12K medium with L-glutamine (Corning, Manassas VA, USA) supplemented with 10% FBS (GE Healthcare, Logan UT, USA) and medium for LoVo/DX additionally contained 10 µg *per* 100 mL doxorubicin (Accord, North Harrow, UK). MV-4-11 cells were cultured in RPMI with GlutaMAX (Gibco, Grand Island NY, USA) supplemented with 10% FBS and 1 mM sodium pyruvate (Sigma-Aldrich, Steinheim, Germany). All culture media contained antibiotics: 100 U mL^–1^ penicillin and 100 µg mL^–1^ streptomycin (Polfa Tarchomin, Warsaw, Poland, and Sigma-Aldrich, Steinheim, Germany, respectively).

#### 2.3.2. Antiproliferative Activity Assay

The cells were seeded onto 96-well plates in respective culture media, and after 24 h, the tested compounds were dissolved in DMSO (Avantor, Gliwice, Poland) to the respective concentration of 10 mg mL^–1^. Serial dilutions of each compound were next prepared in culture media, and introduced to the tested cells for 72 h of incubation. The evaluation of cytotoxicity was performed by means of the sulforhodamine B (SRB) assay. Briefly, 50% cold TCA (Avantor, Gliwice, Poland) was added to each well of 96-well plates with untreated cells or the cells exposed to the tested compounds for 1 h. Next, the plates were washed with tap water and 0.1% SRB (Sigma-Aldrich, Steinheim, Germany) in 1% acetic acid (Avantor, Gliwice, Poland) was added for 30 min. Unbound SRB was next washed out with 1% acetic acid and 10 mM TRIS (Sigma-Aldrich, Steinheim, Germany) was added to each well in order to extract SRB from the cells. The absorbance of solution of each well was read at a 540 nm wavelength using the plate reader Synergy H4 (BioTek Instruments, Winooski VT, USA). 

The IC_50_ values (the concentration resulting in 50% of proliferation inhibition) for each compound of each cell line were calculated using the Cheburator 0.4, Dmitry Nevozhay software [35]. The cells were also exposed to reference drugs cisplatin and doxorubicin (Accord, North Harrow, UK). Each experiment was repeated at least three times separately.

## 3. Results

### 3.1. Precursor Design and Synthesis

The synthesis of desired dimeric polyether ionophores began from the preparation of azide and the respective propargyl components (Scheme 1) as compatible partners for the CuAAC ‘click’ reactions (Scheme 2). The terminal alkynes were synthesized from all polyether ionophores (i.e., lasalocid acid (**LAS**), monensin (**MON**), and salinomycin (**SAL**), and pentacyclic triterpenoid betulinic acid (**BET**)), and they included both *N*-propargyl amides and propargyl esters obtained through the chemical modification of carboxyl group of these biomolecules. Briefly, most *N*-propargyl amides (compound **6**, **8**, and **11**) were obtained in the reaction between **MON**, **BET**, or **SAL** and propargylamine in the presence of DCC and HOBt, which were used as a coupling agent and an activator, respectively [18,19,33]. However, the synthesis of *N*-propargyl amide of **LAS** (compound **4**) was more challenging as the DCC/HOBt-activated reaction did not lead to the expected product. Gratifyingly, the use of HOSu instead of HOBt resulted in the formation of amide **4** with a satisfactory 54% yield. 

With respect to propargyl esters, all (compound **5**, **7**, **9** and **12**) were synthesized by the direct alkylation of the carboxylate ions (i.e., in the S_N_2 reaction between respective biomolecule and propargyl bromide in the presence of DBU as an effective nucleophilic catalyst [20,34]). To expand the structural diversity and for better structure-activity relationship (SAR) studies, the propargyl carbonate of **BET** (compound **10**) was also obtained via the *O*-acylation of its C3 hydroxyl, and was included in the series. On the other hand, the azide partner for the ‘click’ reactions (i.e., C20-*epi*-azidosalinomycin (compound **13**)) was conveniently prepared from **SAL** by the stereoselective Mitsunobu reaction using DPPA as a nucleophilic agent [26]. As expected, this reaction took place exclusively at the C20 position with the inversion of the absolute configuration. 

The structure and purity of the obtained compounds were determined on the basis of NMR, FT-IR, and ESI MS analysis, and whenever possible, compared with the data found in the reference literature [18,19,20,33,34]. Moreover, the ^1^H and ^13^C NMR spectra of all precursors synthesized for the first time (i.e., compounds **4**–**5** and **10**) are included in the Supplementary Materials (Figures S1–S6). Briefly, major evidence of the formation of targeted propargyl moieties is the presence of two characteristic bands in the FT-IR spectra with the maximum at ~3310 cm^–1^ and ~2130 cm^–1^; these bands can be assigned to the *ν*(≡C–H) and *ν*(C≡C) stretching vibrations, respectively (Supplementary Materials, Table S1). In the FT-IR spectrum of azide **13**, the band of the highest analytical significance was that assigned to the *ν*(N_3_) stretching vibrations with the maximum at ~2098 cm^–1^ (Supplementary Materials, Table S1). On the other hand, in the ^1^H NMR spectra of the synthesized series of propargyl precursors, the characteristic signal of terminal acetylenic proton was observed as a very narrow triplet (*J* = 2.4 Hz or 2.5 Hz) in the range of *δ* 2.05–2.58 ppm (Supplementary Materials, Table S1).

### 3.2. Dimerization of Polyether Ionophores 

Having access to a library of propargyl **4**–**12** and azide **13** components (Scheme 1), a series of CuAAC reactions was successfully completed, according to the standard Meldal protocol (Scheme 2). Importantly, despite the multifunctional nature of polyether ionophores and **BET**, all these ‘click’ reactions proceeded cleanly, and resulted in nine triazole-linked dimers (compounds **14**–**22**) obtained in 37–81% of yield after isolation. As **SAL** esters with hydroxamic acids have been identified recently as more effective anticancer and antimicrobial drug candidates than the native structure [16,36,37], we also decided to conjugate one of the selected dimers (compound **22**) with benzhydroxamic and salicylhydroxamic acid, which finally led to the formation of two novel dimeric structures **23** and **24** with ~30% yield. 

Spectroscopically, none of the characteristic bands like *ν*(≡C–H), *ν*(C≡C) and *ν*(N_3_) assigned to propargyl and azide precursors, respectively, was observed in the FT-IR spectra of the dimeric products **14**–**24**, which clearly demonstrated that both azide and alkyne substrates were completely consumed during the CuAAC reactions. The formation of 1,2,3-triazole linkage in dimers **14**–**24** was also directly supported by the ^1^H and ^13^C NMR analysis. First, in the ^1^H NMR spectra of the target compounds, the acetylene proton peaks disappeared, while a singlet characteristic of triazole proton was found in the *δ* 7.48–7.82 ppm range (Supplementary Materials, Table S2). Second, in the ^13^C NMR spectra of these compounds, the C4 signal of the 1,2,3-triazole system appeared at *δ* ~143.0–144.0 ppm (Supplementary Materials, Table S2). The ESI mass spectra also confirmed the formation of the desired products, with [M + H + Na]^2+^ or [M + 2Na]^2+^ as the main peaks (intensity 100%) in most cases (Supplementary Materials, Figures S29–S31).

### 3.3. Antiproliferative Activity 

**Figure 2:**
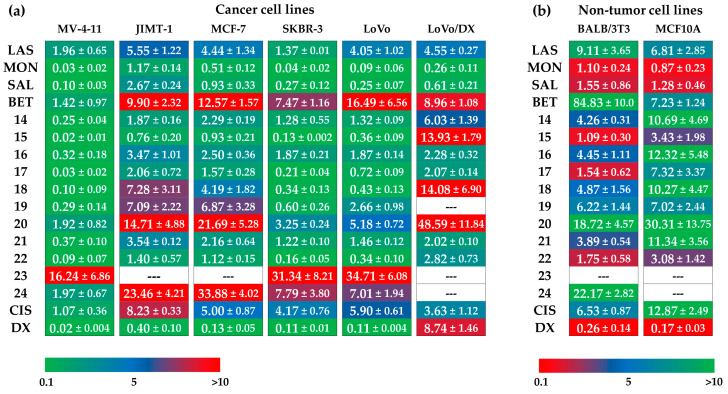
Antiproliferative activity of tested compounds against (**a**) cancer cell lines, and (**b**) non-tumor cell lines, where **LAS**, **MON**, and **SAL** = sodium salt of lasalocid acid, monensin, and salinomycin, respectively, **BET** = betulinic acid, **CIS** = cisplatin, and **DX** = doxorubicin. Data are given as IC_50_ in µM (mean ± SD).

Polyether ionophores (**LAS**, **MON**, **SAL**) in their sodium salts form, **BET**, all newly synthesized dimers **14**–**24**, and two commonly used anticancer drugs, cisplatin and doxorubicin, were evaluated for in vitro antiproliferative activity against a panel of six cancer cell lines: human acute myeloid leukemia cell line (MV-4-11), three human breast cancer cell lines with different characteristics (JIMT-1, MCF-7, SKBR-3), and human colon adenocarcinoma cell lines including the drug-sensitive variant (LoVo), but also its drug-resistant sub-line with the ABCB1 overexpression (LoVo/DX). The cytotoxic effects were studied on normal embryonic fibroblast (BALB/3T3) and normal mammary epithelial cell line (MCF10A); the data are summarized in Figure 2. 

**Figure 3:**
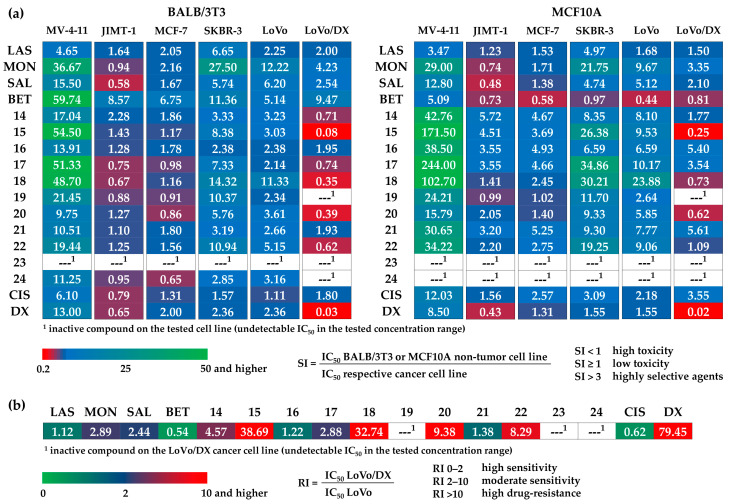
The calculated values of the (**a**) selectivity indexes (SI) for BALB/3T3 (left side) and MCF10A (right side) non-tumor cell lines, and (**b**) resistance indexes (RI) of the tested compounds.

Unmodified polyether ionophores and **BET** were found to be active at micromolar concentrations against all cancer cell lines studied, while the most potent agents among them were **MON** and **SAL**. Taking these two biomolecules into account, **MON** was found to be on average ~2–3 times more active than **SAL**, which is in good agreement with our previously published data [38]. Moreover, all parent compounds exhibited low toxicity toward both non-tumor cell lines with the calculated values of selectivity indexes (SI) >3.0 in most cases (Figure 3a), proving their therapeutic potential in selective targeting of various cancer types. Of note, the activity of **LAS**, **MON**, and **SAL** was superior compared to that exhibited by a standard chemotherapeutic drug, cisplatin. Therefore, it was extremely interesting to establish if the dimeric ionophores could also be considered as promising agents in killing cancer cells.

Importantly, similar to the native structures, the majority of newly synthesized dimers showed the anticancer activity at micromolar concentrations, and in some cases, these effects were even a few times more potent than those of the starting materials and widely used cytostatic drugs. For example, with respect to the JIMT-1 breast cancer cell line, the IC_50_ value of compound **15** (**SAL**-**LAS** dimer) was 0.76 µM, compared to 5.55 µM, 2.67 µM, and 8.23 µM for **LAS**, **SAL**, and cisplatin, respectively. As far as breast cancer is concerned, the effects of derivative **22** (**SAL**-**SAL** dimer) were also noticeable; with IC_50_ of 0.16 µM in SKBR-3 cell line, the activity of this compound was superior to that of the parent polyether ionophore (**SAL**, IC_50_ = 0.27 µM) and cisplatin (IC_50_ = 4.17 µM). Aside from **15** and **22**, the group of dimers that might be recognized as more anticancer active structures than the reference agents, should comprise compounds **14**, **17**, and **18**. On the other hand, hydroxamic acid conjugates **23** and **24** together with dimer **20** seemed to show the weakest antiproliferative activity on the cancer cell lines studied.

According to the data in Figure 2, practically all novel dimers showed low toxicity to the MCF10A normal mammary epithelial cell line. Thus, these compounds showed generally higher SI values than those of the unmodified ionophores as well as those of cisplatin and doxorubicin, which proves their promising therapeutic window. The differences in the IC_50_ values of compounds **14**–**24** in drug-sensitive and drug-resistant variants of human colon adenocarcinoma cell line (IC_50_ for LoVo/DX > IC_50_ for LoVo) might suggest the much lower activity of dimers in the treatment of cancer cells with the MDR phenotype. However, looking closer at the values of resistance indexes (RI) (Figure 3b), two compounds (**SAL**-**MON** dimer **16**, and **SAL**-**SAL** dimer **21**) with RI values of 1.22 and 1.38, respectively, were found to effectively overcome the resistance of the LoVo/DX cell line, being more potent than the respective starting materials (**SAL** RI = 2.44, and **MON** RI = 2.89).

## 4. Discussion

Despite the complex structure and multifunctional nature of polyether ionophores studied in this work, lasalocid acid (**LAS**), monensin (**MON**), and salinomycin (**SAL**) were found to be excellent partners for the CuAAC reactions; their ‘click’ conjugation might be completed with satisfactory yields using the very simple Meldal procedure (Scheme 2). As far as propargyl precursors are concerned (Scheme 1), some of them could be obtained on a gram scale on the basis of the procedures described by us and other authors previously [15,18,19,20,33,34], while the other three (compounds **4**, **5**, and **10**) were synthesized for the first time. 

In 2016, Jiang and co-workers applied successfully the Mitsunobu reaction to the site-specific stereoselective modification of the **SAL** biomolecule, leading to the introduction of the azide group with inversed absolute configuration at the C20 position [26]. On the basis of the results of the semi-empirical methods (data not shown) and the analysis of the single crystal X-ray diffraction (scXRD) structures of the literature-known derivatives of C20-*epi*-salinomycin [22,26], we have been convinced that the inversion of the C20 configuration should significantly reduce the steric hindrance of the newly introduced moieties at this position, and thus provide a very valuable site for the chemical modification of **SAL**, especially by sterically hindered counterparts. Taking all these facts into account, C20-*epi*-azidosalinomycin **13** seemed to be the ideal candidate for dimerization with other biomolecules to form a completely new class of multivalent structures.

Of the large group of polyether ionophores that could be used for the CuAAC reactions, we decided to use three. Aside from **SAL**, we also selected **LAS** and **MON** for our project because of the well-known potential of these agents in selective targeting of cancer cells of various origin including those with stem-like as well as multi-drug resistance (MDR) phenotype [6,7,8,9,10,11,12,13,14]; **BET**, with its proven activity against therapy-resistant and refractory tumors [31,32], was also included in our studies. Some earlier studies have clearly indicated that the formation of dimers of **SAL** might be beneficial to improve potency, and in this context, particularly interesting seems to be the chemical modification of the C20 position of this ionophore [29,30]. In 2017, Liu and co-workers showed that the **SAL** triazole dimers (structures **1**–**2**, Figure 1b) exhibited preferable growth inhibition in the MCF-7 breast cancer cell line, with even ~5-fold higher anticancer activity than that of unmodified **SAL** [29]. In the same year, we also synthesized a series of **SAL** homodimers, modified both at the C1 and C20 position, among which the one modified at C20 hydroxyl (compound **3**, Figure 1b) displayed the most promising bioactivity [30]. On the other hand, the cytotoxic analysis of a series of 1,2,3-triazole-tethered **BET** conjugated with AZT has revealed their potential activity toward KB and Hep-G2 human tumor cell lines [33,34]. Taking all of these facts into account, we predicted that the new type of designed dimeric ionophores including their ‘mixed’ structures should also exhibit very interesting properties in the selective killing of cancer cells.

Our study clearly confirmed these findings. The SAR analysis revealed some interesting and noticeable trends. Of note is, for example, that ester-triazole-linked dimers **15**, **17**, and **22** were found to be more anticancer active than the amide-triazole-linked dimers **14**, **16**, and **21**; the only exception of this rule was dimer **19**, which was less active toward cancer cell lines used than the corresponding dimer **18** (Figure 2). The increased antiproliferative activity of structures **15**, **17**, and **22** may be a consequence of the cleavage of ester bonds by cellular esterases, which finally leads to the release of the two highly toxic parent compounds [39]; a similar trend has been observed previously for **SAL** conjugates with floxuridine [40]. 

The selectivity index (SI), an important pharmaceutical parameter, could help to measure the therapeutic window between cytotoxicity and anticancer activity of the tested compounds by dividing the given IC_50_ values for non-tumor cell lines (BALB/3T3 or MCF10A) into those calculated for respective cancer cell lines (MV-4-11, JIMT-1, MCF-7, SKBR-3, LoVo or LoVo/DX) (Figure 3a). The higher the SI of a given agent, the more effective and safe it should be in future in vivo tests. The ideal anticancer drug candidates should be cytotoxic only at very high concentrations in normal cells, and simultaneously show anticancer activity at very low concentrations, thus yielding high SI values that would facilitate the estimation of their possible clinical development. The SI is a widely accepted parameter used to express the in vitro efficacy of tested compounds in the inhibition of cancer cell proliferation; the SI >1.0 identifies agents with activity toward cancer cells greater than toxicity against non-tumor cells, while the compounds displaying SI values greater than 3.0 are considered as highly selective agents [41]. On the basis of the values of the IC_50_ and SI parameters, the cancer cell lines most sensitive to the action of dimeric polyether ionophores seemed to be MV-4-11, SKBR-3, and LoVo cell lines. Moreover, it should be stressed here that the synthesized dimers were generally much less toxic to non-tumor cancer cell lines compared to the starting material(s) and two commonly used cytostatic drugs, cisplatin and doxorubicin. On the other hand, the MDR variant of LoVo cell line (LoVo/DX) was the most resistant (high IC_50_ values *plus* SI <1.0 in many cases) to the therapeutic effects of all dimers, and thus it may suggest the potential use of these new agents, particularly in primary cancer treatment (first-line therapy).

Aside from SI, the resistance index (RI) is another important parameter that indicates how many times the MDR sub-line (LoVo/DX) is resistant relative to its parent cell line (LoVo); when RI = 0–2, the cells are sensitive to the action of the tested compounds, RI = 2–10 means that the cancer cells show moderate sensitivity to the agents used, while RI >10 indicates strong drug-resistance (Figure 3b) [41]. In this context, although the amide-triazole-linked dimers showed less promising IC_50_ values, and thus they might seem to be less effective in killing cancer cells, these agents, especially compounds **16** and **21**, were found to be more efficient in overcoming the resistance of the LoVo/DX sub-line, characterized by the ABCB1 overexpression, than the corresponding ester-triazole-linked dimers, but also the respective unmodified ionophore (i.e., **SAL** and **MON**).

Finally, compared with the previously obtained multivalent structures of **SAL** [19,29,30] and its other derivatives [6,15,16,17,18,19,20,21,22,23,24,25,26,27], the newly synthesized ester-triazole-linked dimers, particularly ‘mixed’ **SAL**-**LAS** dimer **15** and **SAL**-**SAL** homodimer **22**, should be classified with no doubt not only as the most promising dimeric structures synthesized so far, but also as the most active **SAL** derivatives in general. Therefore, our results seem to be a good starting point for further studies on the dimerization of polyether ionophores, which is a very efficient synthetic strategy to generate highly bioactive drug candidates. It also opens a new way of chemical modification not only of **SAL**, but also other biomolecules belonging to this interesting class of biologically active compounds.

## 5. Conclusions

To sum up, we reported here the design, synthesis, full characterization, and in vitro anticancer activity of a new class of 11 dimeric products that were generated with satisfactory yields using the CuAAC reactions. This new class includes salinomycin (**SAL**) homodimers, but also a series of ‘mixed’ structures resulting from combinations of the **SAL** biomolecule with other biologically active agents including lasalocid acid (**LAS**), monensin (**MON**), and betulinic acid (**BET**). The ester-triazole-linked dimers showed favorable inhibition of human breast cancer, colon adenocarcinoma as well as leukemia cell proliferation when used at low micromolar concentrations, indicating the C20 position of **SAL** with an inversed absolute configuration as a very valuable position for dimerization, especially by sterically hindered partners. Of note is that selected dimeric structures were even a few-times more anticancer active than both the parent compound(s) and the commonly used chemotherapeutics. The inspection of selective targeting of cancer cells by the dimers, expressed in terms of their selectivity index (SI) values, revealed that the newly synthesized products were also less toxic toward non-tumor cancer cell lines compared to the starting material(s), cisplatin, and doxorubicin. Furthermore, two amide-triazole-linked dimers were active against the cancer cell line LoVo/DX that expressed the multi-drug resistance (MDR) phenotype.

Our findings support the dimerization of polyether antibiotics as a fruitful strategy to generate very promising anticancer drug candidates; such a concept is also a good starting point for further structural modifications as well as drug discovery optimization. The gram-scale synthesis of azide and propargyl precursors and the operational simplicity of the described synthetic manipulations promise access to sufficient amounts of dimeric structures for further comprehensive bioassays and mechanistic studies. Such investigations are under way, and will be reported in due course.

## Supplementary Materials

The following are available online at https://www.mdpi.com/2218-273X/10/7/1039/s1, Figure S1: The ^1^H NMR spectrum of **4** in dichloromethane-d_2_, Figure S2: The ^13^C NMR spectrum of **4** in dichloromethane-d_2_, Figure S3: The ^1^H NMR spectrum of **5** in chloroform-d, Figure S4: The ^13^C NMR spectrum of **5** in chloroform-d, Figure S5: The ^1^H NMR spectrum of **10** in chloroform-d, Figure S6: The ^13^C NMR spectrum of **10** in chloroform-d, Figure S7: The ^1^H NMR spectrum of **14** in chloroform-d, Figure S8: The ^13^C NMR spectrum of **14** in chloroform-d, Figure S9: The ^1^H NMR spectrum of **15** in chloroform-d, Figure S10: The ^13^C NMR spectrum of **15** in chloroform-d, Figure S11: The ^1^H NMR spectrum of **16** in chloroform-d, Figure S12: The ^13^C NMR spectrum of **16** in chloroform-d, Figure S13: The ^1^H NMR spectrum of **17** in chloroform-d, Figure S14: The ^13^C NMR spectrum of **17** in chloroform-d, Figure S15: The ^1^H NMR spectrum of **18** in chloroform-d, Figure S16: The ^13^C NMR spectrum of **18** in chloroform-d, Figure S17: The ^1^H NMR spectrum of **19** in chloroform-d, Figure S18: The ^13^C NMR spectrum of **19** in chloroform-d, Figure S19: The ^1^H NMR spectrum of **20** in chloroform-d, Figure S20: The ^13^C NMR spectrum of **20** in chloroform-d, Figure S21: The ^1^H NMR spectrum of **21** in chloroform-d, Figure S22: The ^13^C NMR spectrum of **21** in chloroform-d, Figure S23: The ^1^H NMR spectrum of **22** in chloroform-d, Figure S24: The ^13^C NMR spectrum of **22** in chloroform-d, Figure S25: The ^1^H NMR spectrum of **23** in chloroform-d, Figure S26: The ^13^C NMR spectrum of **23** in chloroform-d, Figure S27: The ^1^H NMR spectrum of **24** in chloroform-d, Figure S28: The ^13^C NMR spectrum of **24** in chloroform-d, Figure S29: The ESI mass spectra of a mixture of **14**, **15**, **16** and **17** with NaClO_4_ at cv = 30 V, Figure S30: The ESI mass spectra of a mixture of **18**, **19**, **20** and **21** with NaClO_4_ at cv = 30 V, Figure S31: The ESI mass spectra of a mixture of **22**, **23**, and **24** with NaClO_4_ at cv = 30 V, Table S1: The yields of the synthesis, and the analytical bands in the FT-IR spectra (wavenumbers in cm^–1^) and signals in the ^1^H and ^13^C NMR spectra (*δ* in ppm) of propargyl and azide partners for the CuAAC reaction, Table S2: The yields of the synthesis, and the analytical signals in the ^1^H and ^13^C NMR spectra of novel dimeric polyether ionophores.

## Dedication

We would like to dedicate this article to Professor Bogumił Brzezinski in honor of his 77^th^ birthday.

## References

[1] Antoszczak M., Huczyński A. (2019). Salinomycin and its derivatives – A new class of multiple-targeted “magic bullets”. Eur. J. Med. Chem..

[2] Antoszczak M., Steverding D., Huczyński A. (2019). Anti-parasitic activity of polyether ionophores. Eur. J. Med. Chem..

[3] Rutkowski J., Brzezinski B. (2013). Structures and properties of naturally occurring polyether antibiotics. Biomed. Res. Int..

[4] Kevin II D.A., Meujo D.A., Hamann M.T. (2009). Polyether ionophores: Broad-spectrum and promising biologically active molecules for the control of drug-resistant bacteria and parasites. Expert Opin. Drug Discov..

[5] Antoszczak M. (2019). A comprehensive review of salinomycin derivatives as potent anticancer and anti-CSCs agents. Eur. J. Med. Chem..

[6] Huczyński A., Rutkowski J., Borowicz I., Wietrzyk J., Maj E., Brzezinski B. (2013). One-pot synthesis and cytotoxicity studies of new Mannich base derivatives of polyether antibiotic – lasalocid acid. Bioorg. Med. Chem. Lett..

[7] Kim K.Y., Kim S.H., Yu S.N., Park S.G., Kim Y.W., Nam H.W., An H.H., Yu H.S., Kim Y.W., Ji J.H. (2017). Lasalocid induces cytotoxic apoptosis and cytoprotective autophagy through reactive oxygen species in human prostate cancer PC-3 cells. Biomed. Pharmacother..

[8] Ketola K., Vainio P., Fey V., Kallioniemi O., Iljin K. (2010). Monensin is a potent inducer of oxidative stress and inhibitor of androgen signaling leading to apoptosis in prostate cancer cells. Mol. Cancer Ther..

[9] Vanneste M., Huang Q., Li M., Moose D., Zhao L., Stamnes M.A., Schultz M., Wu M., Henry M.D. (2019). High content screening identifies monensin as an EMT-selective cytotoxic compound. Sci. Rep..

[10] Wang X., Wu X., Zhang Z., Ma C., Wu T., Tang S., Zeng Z., Huang S., Gong C., Yuan C. (2018). Monensin inhibits cell proliferation and tumor growth of chemo-resistant pancreatic cancer cells by targeting the EGFR signaling pathway. Sci. Rep..

[11] Park W.H., Seol J.G., Kim E.S., Kang W.K., Im Y.H., Jung C.W., Kim B.K., Lee Y.Y. (2002). Monensin-mediated growth inhibition in human lymphoma cells through cell cycle arrest and apoptosis. Br. J. Haematol..

[12] Gupta P.B., Onder T.T., Jiang G., Tao K., Kuperwasser C., Weinberg R.A., Lander E.S. (2009). Identification of selective inhibitors of cancer stem cells by high-throughput screening. Cell.

[13] Naujokat C., Steinhart R. (2012). Salinomycin as a drug for targeting human cancer stem cells. J. Biomed. Biotechnol..

[14] Antoszczak M. (2019). A medicinal chemistry perspective on salinomycin as a potent anticancer and anti-CSCs agent. Eur. J. Med. Chem..

[15] Klejborowska G., Jędrzejczyk M., Stępczyńska N., Maj E., Wietrzyk J., Huczyński A. (2019). Antiproliferative activity of ester derivatives of monensin A at the C-1 and C-26 positions. Chem. Biol. Drug Des..

[16] Li B., Wu J., Zhang W., Li Z., Chen G., Zhou Q., Wu S. (2017). Synthesis and biological activity of salinomycin-hydroxamic acid conjugates. Bioorg. Med. Chem. Lett..

[17] Huczyński A., Klejborowska G., Antoszczak M., Maj E., Wietrzyk J. (2015). Anti-proliferative activity of monensin and its tertiary amide derivatives. Bioorg. Med. Chem. Lett..

[18] Skiera I., Antoszczak M., Trynda J., Wietrzyk J., Boratyński P., Kacprzak K., Huczyński A. (2015). Antiproliferative activity of polyether antibiotic-*Cinchona* alkaloid conjugates obtained via click chemistry. Chem. Biol. Drug Des..

[19] Antoszczak M., Maj E., Stefańska J., Wietrzyk J., Janczak J., Brzezinski B., Huczyński A. (2014). Synthesis, antiproliferative and antibacterial activity of new amides of salinomycin. Bioorg. Med. Chem. Lett..

[20] Antoszczak M., Popiel K., Stefańska J., Wietrzyk J., Maj E., Janczak J., Michalska G., Brzezinski B., Huczyński A. (2014). Synthesis, cytotoxicity and antibacterial activity of new esters of polyether antibiotic – salinomycin. Eur. J. Med. Chem..

[21] Versini A., Colombeau L., Hienzsch A., Gaillet C., Retailleau P., Debieu S., Müller S., Cañeque T., Rodriguez R. (2020). Salinomycin derivatives kill breast cancer stem cells by lysosomal iron targeting. Chem. Eur. J..

[22] Li Y., Shi Q., Shao J., Yuan Y., Yang Z., Chen S., Zhou X., Wen S., Jiang Z.X. (2018). Synthesis and biological evaluation of 20-*epi*-amino-20-deoxysalinomycin derivatives. Eur. J. Med. Chem..

[23] Mai T.T., Hamaï A., Hienzsch A., Cañeque T., Müller S., Wicinski J., Cabaud O., Leroy C., David A., Acevedo V. (2017). Salinomycin kills cancer stem cells by sequestering iron in lysosomes. Nat. Chem..

[24] Borgström B., Huang X., Hegardt C., Oredsson S., Strand D. (2017). Structure-activity relationships in salinomycin: Cytotoxicity and phenotype selectivity of semi-synthetic derivatives. Chem. Eur. J..

[25] Zhang W., Wu J., Li B., Xia J., Wu H., Wang L., Hao J., Zhou Q., Wu S. (2016). Synthesis and biological activity evaluation of 20-*epi*-salinomycin and its 20-*O*-acyl derivatives. RSC Adv..

[26] Shi Q., Li Y., Bo S., Li X., Zhao P., Liu Q., Yang Z., Cong H., Deng H., Chen M. (2016). Discovery of a ^19^F MRI sensitive salinomycin derivative with high cytotoxicity towards cancer cells. Chem. Commun..

[27] Borgström B., Huang X., Pošta M., Hegardt C., Oredsson S., Strand D. (2013). Synthetic modification of salinomycin: Selective *O*-acylation and biological evaluation. Chem. Commun..

[28] Huczyński A., Domańska A., Paluch I., Stefańska J., Brzezinski B., Bartl F. (2008). Synthesis of new semi-synthetic dipodands and tripodands from naturally occurring polyether ionophores. Tetrahedron Lett..

[29] Huang M., Deng Z., Tian J., Liu T. (2017). Synthesis and biological evaluation of salinomycin triazole analogues as anticancer agents. Eur. J. Med. Chem..

[30] Antoszczak M., Maj E., Borgström B., Oredsson S., Huczyński A., Wietrzyk J., Strand D. (2017). Bivalent polyether ionophores: Synthesis and biological evaluation of C*_2_*-symmetric salinomycin dimers. Tetrahedron Lett..

[31] Mullauer F.B., Kessler J.H., Medema J.P. (2010). Betulinic acid, a natural compounds with potent anticancer effects. Anticancer Drugs.

[32] Fulda S. (2009). Betulinic acid: A natural product with anticancer activity. Mol. Nutr. Food Res..

[33] Dang Thi T.A., Kim Tuyet N.T., Pham The C., Thanh Nguyen H., Ba Thi C., Thi Phuong H., Van Boi L., Van Nguyen T., D’hooghe M. (2015). Synthesis and cytotoxic evaluation of novel amide-triazole-linked triterpenoid-AZT conjugates. Tetrahedron Lett..

[34] Dang Thi T.A., Kim Tuyet N.T., Pham The C., Thanh Nguyen H., Ba Thi C., Doan Duy T., D’hooghe M., Van Nguyen T. (2014). Synthesis and cytotoxic evaluation of novel ester-triazole-linked triterpenoid-AZT conjugates. Bioorg. Med. Chem. Lett..

[35] Nevozhay D. (2014). Cheburator software for automatically calculating drug inhibitory concentrations from in vitro screening assays. PLoS One.

[36] Sulik M., Stępień K., Stefańska J., Huczyński A., Antoszczak M. (2020). Antibacterial activity of singly and doubly modified salinomycin derivatives. Bioorg. Med. Chem. Lett..

[37] Antoszczak M., Steverding D., Sulik M., Janczak J., Huczyński A. (2019). Anti-trypanosomal activity of doubly modified salinomycin derivatives. Eur. J. Med. Chem..

[38] Antoszczak M., Klejborowska G., Kruszyk M., Maj E., Wietrzyk J., Huczyński A. (2015). Synthesis and antiproliferative activity of silybin conjugates with salinomycin and monensin. Chem. Biol. Drug Des..

[39] Rooseboom M., Commandeur J.N., Vermeulen N.P. (2004). Enzyme catalyzed activation of anticancer prodrugs. Pharmacol. Rev..

[40] Huczyński A., Antoszczak M., Kleczewska N., Lewandowska M., Maj E., Stefańska J., Wietrzyk J., Janczak J., Celewicz L. (2015). Synthesis and biological activity of salinomycin conjugates with floxuridine. Eur. J. Med. Chem..

[41] Antoszczak M., Urbaniak A., Delgado M., Maj E., Borgström B., Wietrzyk J., Huczyński A., Yuan Y., Chambers T.C., Strand D. (2018). Biological activity of doubly modified salinomycin analogs – Evaluation in vitro and ex vivo. Eur. J. Med. Chem..

