# Supplementary material for: Synthesis and Anticancer Activity of Dimeric Polyether Ionophores
Source: Biomolecules. 2020 Jul 12;10(7):1039. doi: 10.3390/biom10071039 (PMC7408349; doi:10.3390/biom10071039)
Supplement: Supplementary file 1 [file biomolecules-10-01039-s001.pdf]

Supporting Information

Synthesis and anticancer activity of dimeric polyether ionophores

Michał Sulik [a], Ewa Maj [b], Joanna Wietrzyk [b], Adam Huczyński [a], and Michał Antoszczak [a,*]

[a] *Department of Medical Chemistry, Faculty of Chemistry, Adam Mickiewicz University, Uniwersytetu Poznańkisego 8, 61–614 Poznań, Poland*

[b] *Hirszfeld Institute of Immunology and Experimental Therapy, Polish Academy of Sciences, Rudolfa Weigla 12, 53–114 Wrocław, Poland*

Index

Spectroscopic and spectrometric analysis of synthesized compounds .... – S3 –

FT-IR and NMR analysis of propargyl and azide components .... – S3 –

^{1}H and ^{13}C NMR analysis of newly synthesized compounds... – S3 –

ESI MS analysis of dimeric polyether ionophores... – S33 –

Spectroscopic and spectrometric analysis of synthesized compounds

FT-IR and NMR analysis of propargyl and azide components

Table S1. The yields of the synthesis, and the analytical bands in the FT-IR spectra (wavenumbers in cm^{-1}) and signals in the ^{1}H and ^{13}C NMR spectra (δ in ppm) of propargyl and azide partners for the CuAAC reaction.

	**No.**	**4**	**5**	**6**	**7**	**8**	**9**	**10**	**11**	**12**	**13**
	**Yield (%)**	54	60	51	65	51	60	15	88	90	70
**FT-IR** KBr tablet	ν**(≡C–H)**	3313	3311	3315	3314	3311	3310	3297	3319	3314	-----
	ν**(C≡C)**	2125	2130	2120	2128	2120	2129	2130	2128	2127	-----
	ν**(N_3)**	-----	-----	-----	-----	-----	-----	-----	-----	-----	2098
^{1}**H NMR** in CD_2Cl_2	δ**(≡C–H)**	2.32 (t, J = 2.5 Hz, 1H)	2.58[(a)] (t, J = 2.5 Hz, 1H)	signal overlapped	2.56 (t, J = 2.5 Hz, 1H)	2.22 (t, J = 2.5 Hz, 1H)	2.48 (t, J = 2.4 Hz, 1H)	2.51[(a)] (t, J = 2.4 Hz, 1H)	2.16 (t, J = 2.5 Hz, 1H)	2.05[(b)] (t, J = 2.5 Hz, 1H)	-----
^{13}**C NMR** in CD_2Cl_2	δ**(C=O) amide/ester**	170.6	171.1[(a)]	175.7[(a)]	175.1	176.0	175.5	154.5[(a,c)]	175.2	173.9[(b)]	-----

[(a)] in $CDCl_3$; [(b)] in C_6D_6; [(c)] δ(C=O)carbonate in **10**

^{1}*H and* ^{13}*C NMR analysis of newly synthesized compounds*

Table S2. The yields of the synthesis, and the analytical signals in the ^{1}H and ^{13}C NMR spectra of novel dimeric polyether ionophores.

**No.**	**Yield (%)**	**Analytical NMR signals (ppm) in $CDCl_3$**					
		δ**(C=O) carboxylate**	δ**(C=O) amide/ester**	δ**(H–N) amide**	δ**(CH_2–O) ester**	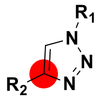	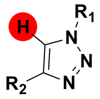
**14**	70	184.5	170.1	7.28–7.19 (m, 1H)	-----	144.5[(a)]	7.82 (s, 1H)
**15**	70	184.4	171.5	-----	5.41 (dd, J = 26.1, 12.6 Hz, 2H)	141.6[(a)]	7.68 (s, 1H)
**16**	63	184.3	175.7	7.15–7.06 (m, 1H)	-----	144.7	7.46 (s, 1H)
**17**	52	184.4	175.0	-----	5.24 (d, J = 12.8 Hz, 1H) 5.16 (d, J = 12.8 Hz, 1H)	142.4	7.63 (s, 1H)
**18**	56	184.6	176.1	6.23 (t, J = 5.5 Hz, 1H)	-----	144.4	7.48 (s, 1H)
**19**	81	184.5	175.7	-----	5.09 (dd, J = 25.8, 12.7 Hz, 2H)	142.5	7.58 (s, 1H)
**20**	37	184.5	155.0[(b)]	-----	5.23–5.15 (m, 2H)[(c)]	141.8	7.67 (s, 1H)
**21**	63	184.4	175.0	6.97 (t, J = 5.1 Hz, 1H)	-----	144.9	7.60 (s, 1H)
**22**	52	184.4	175.2	-----	5.50 (d, J = 12.8 Hz, 1H) 5.25 (d, J = 12.8 Hz, 1H)	142.3	7.66 (s, 1H)
**23**	32	173.5[(d)]	175.3	-----	5.43 (d, J = 12.8 Hz, 1H) second signal overlapped	142.9	7.55 (s, 1H)
**24**	31	173.4[(d)]	175.3	-----	5.50 (d, J = 12.9 Hz, 1H) second signal overlapped	142.9	7.62 (s, 1H)

[(a)] based on the ^{1}H-^{13}C HMBC spectrum; [(b)] δ(C=O)carbonate in **20**; [(c)] δ(CH_2–O)carbonate in **20**; [(d)] δ(C=O)ester after conjugation with hydroxamic acid in **23** and **24**

1) The NMR spectra of newly synthesized propargyl precursors

List of spectra

Figure S1. The ^{1}H NMR spectrum of **4** in dichloromethane-d_2........– S5 –

Figure S2. The ^{13}C NMR spectrum of **4** in dichloromethane-d_2........– S6 –

Figure S3. The ^{1}H NMR spectrum of **5** in chloroform-d........– S7 –

Figure S4. The ^{13}C NMR spectrum of **5** in chloroform-d........– S8 –

Figure S5. The ^{1}H NMR spectrum of **10** in chloroform-d........– S9 –

Figure S6. The ^{13}C NMR spectrum of **10** in chloroform-d........– S10 –

2) The NMR spectra of dimeric polyether ionophores

List of spectra

Figure S7. The ^{1}H NMR spectrum of **14** in chloroform-d........– S11 –

Figure S8. The ^{13}C NMR spectrum of **14** in chloroform-d........– S12 –

Figure S9. The ^{1}H NMR spectrum of **15** in chloroform-d........– S13 –

Figure S10. The ^{13}C NMR spectrum of **15** in chloroform-d........– S14 –

Figure S11. The ^{1}H NMR spectrum of **16** in chloroform-d........– S15 –

Figure S12. The ^{13}C NMR spectrum of **16** in chloroform-d........– S16 –

Figure S13. The ^{1}H NMR spectrum of **17** in chloroform-d........– S17 –

Figure S14. The ^{13}C NMR spectrum of **17** in chloroform-d........– S18 –

Figure S15. The ^{1}H NMR spectrum of **18** in chloroform-d........– S19 –

Figure S16. The ^{13}C NMR spectrum of **18** in chloroform-d........– S20 –

Figure S17. The ^{1}H NMR spectrum of **19** in chloroform-d........– S21 –

Figure S18. The ^{13}C NMR spectrum of **19** in chloroform-d........– S22 –

Figure S19. The ^{1}H NMR spectrum of **20** in chloroform-d........– S23 –

Figure S20. The ^{13}C NMR spectrum of **20** in chloroform-d........– S24 –

Figure S21. The ^{1}H NMR spectrum of **21** in chloroform-d........– S25 –

Figure S22. The ^{13}C NMR spectrum of **21** in chloroform-d........– S26 –

Figure S23. The ^{1}H NMR spectrum of **22** in chloroform-d........– S27 –

Figure S24. The ^{13}C NMR spectrum of **22** in chloroform-d........– S28 –

Figure S25. The ^{1}H NMR spectrum of **23** in chloroform-d........– S29 –

Figure S26. The ^{13}C NMR spectrum of **23** in chloroform-d........– S30 –

Figure S27. The ^{1}H NMR spectrum of **24** in chloroform-d........– S31 –

Figure S28. The ^{13}C NMR spectrum of **24** in chloroform-d........– S32 –

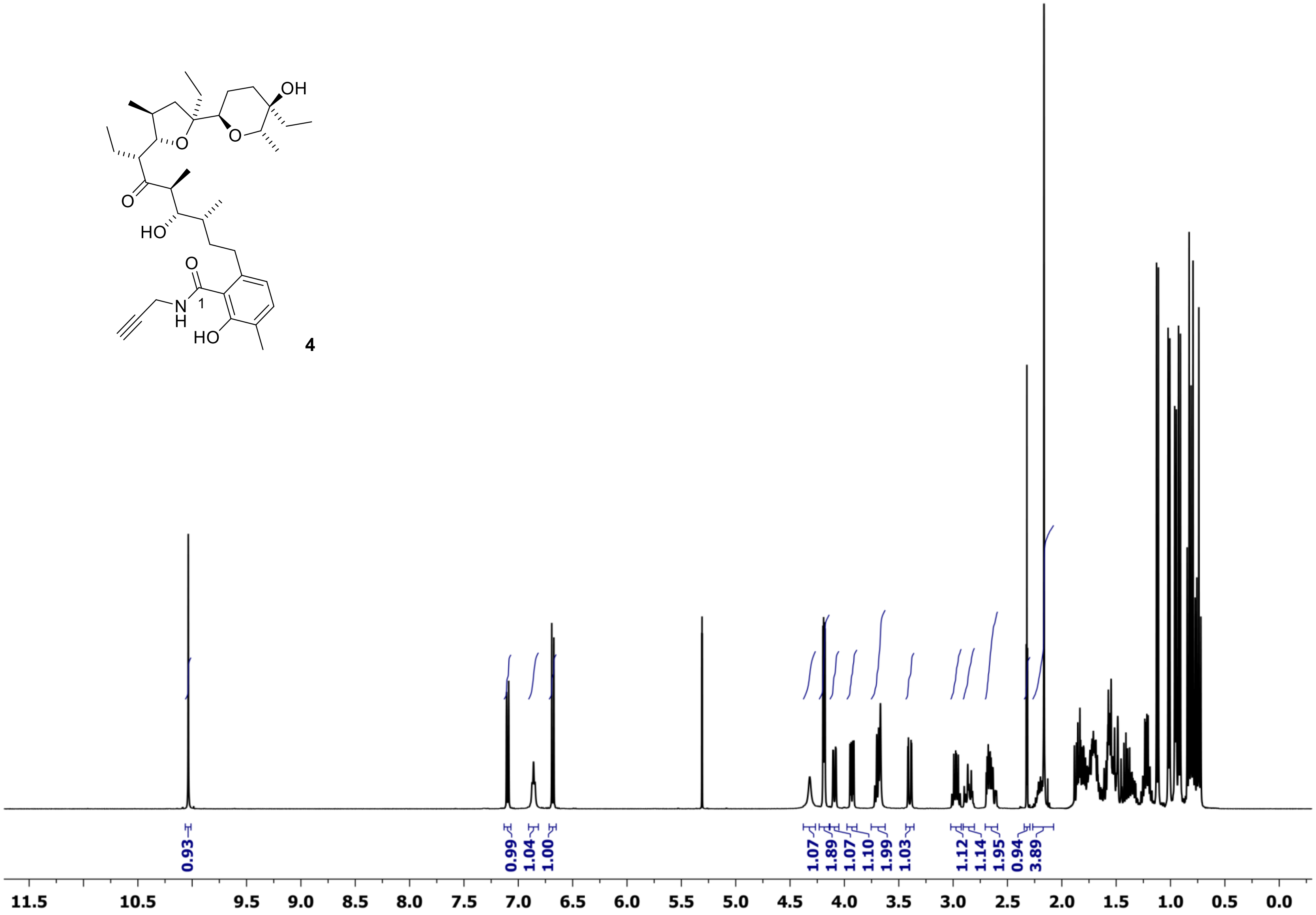

Figure S1. The ^{1}H NMR spectrum of **4** in dichloromethane-d_2.

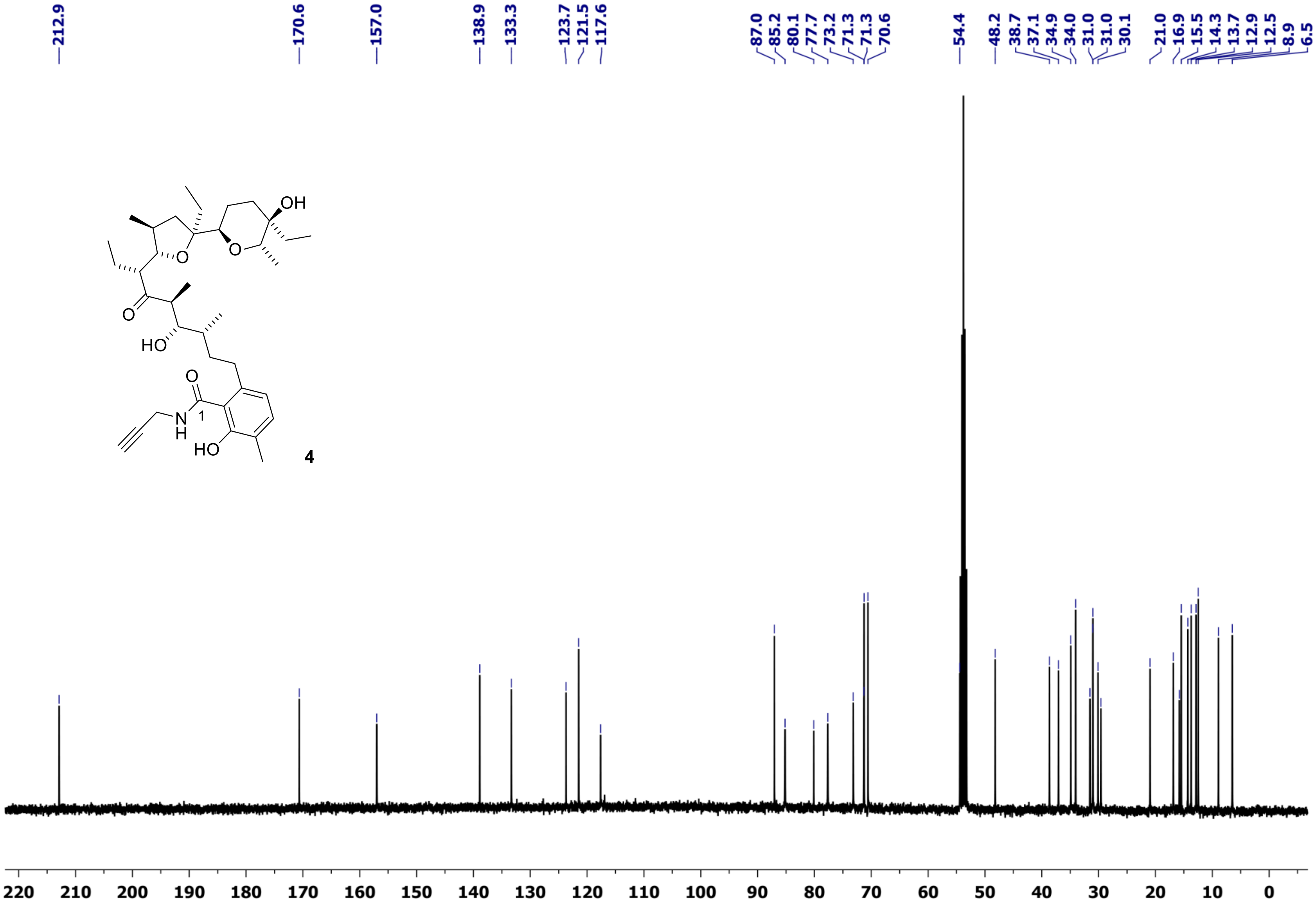

Figure S2. The ^{13}C NMR spectrum of **4** in dichloromethane-d_2.

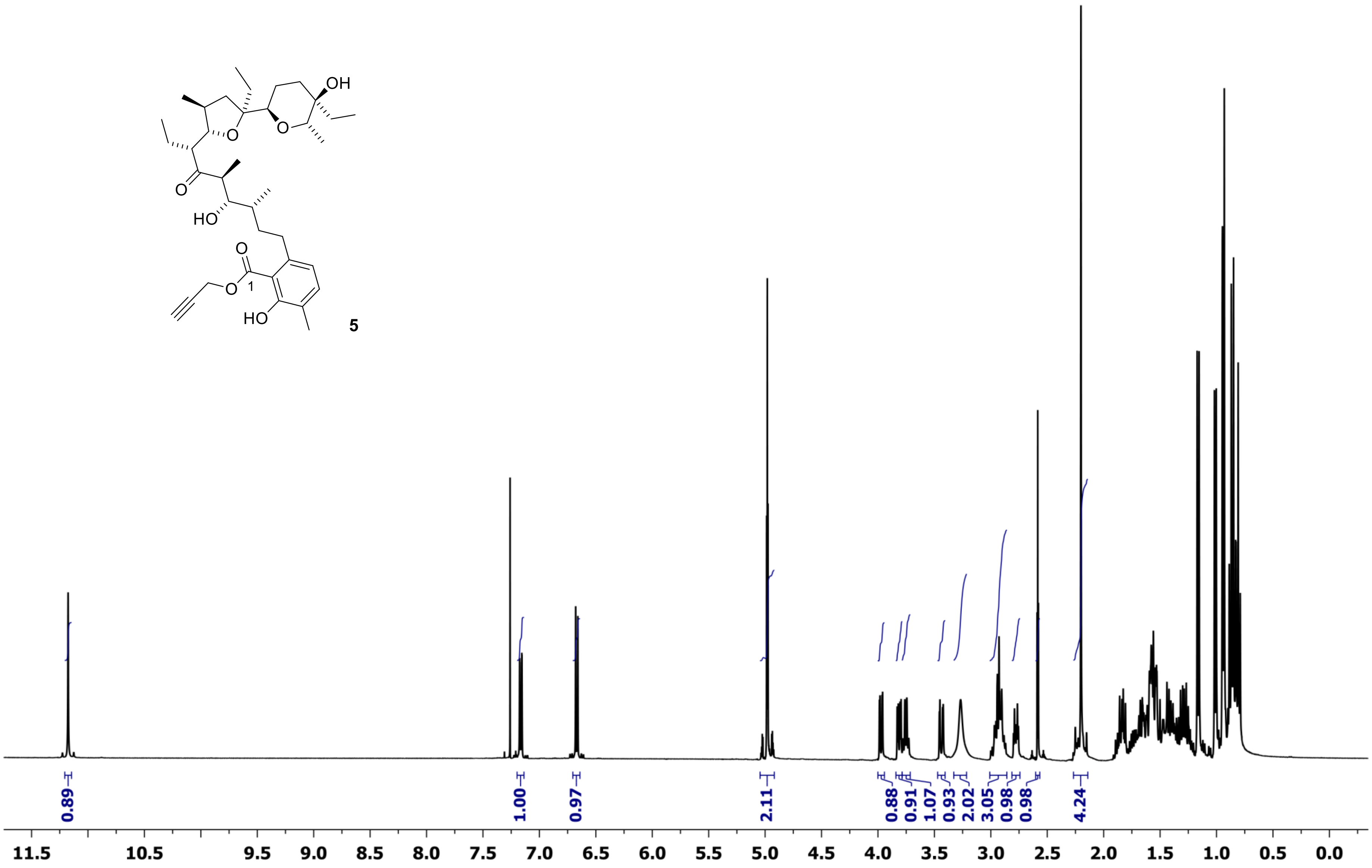

Figure S3. The ^{1}H NMR spectrum of **5** in chloroform-d.

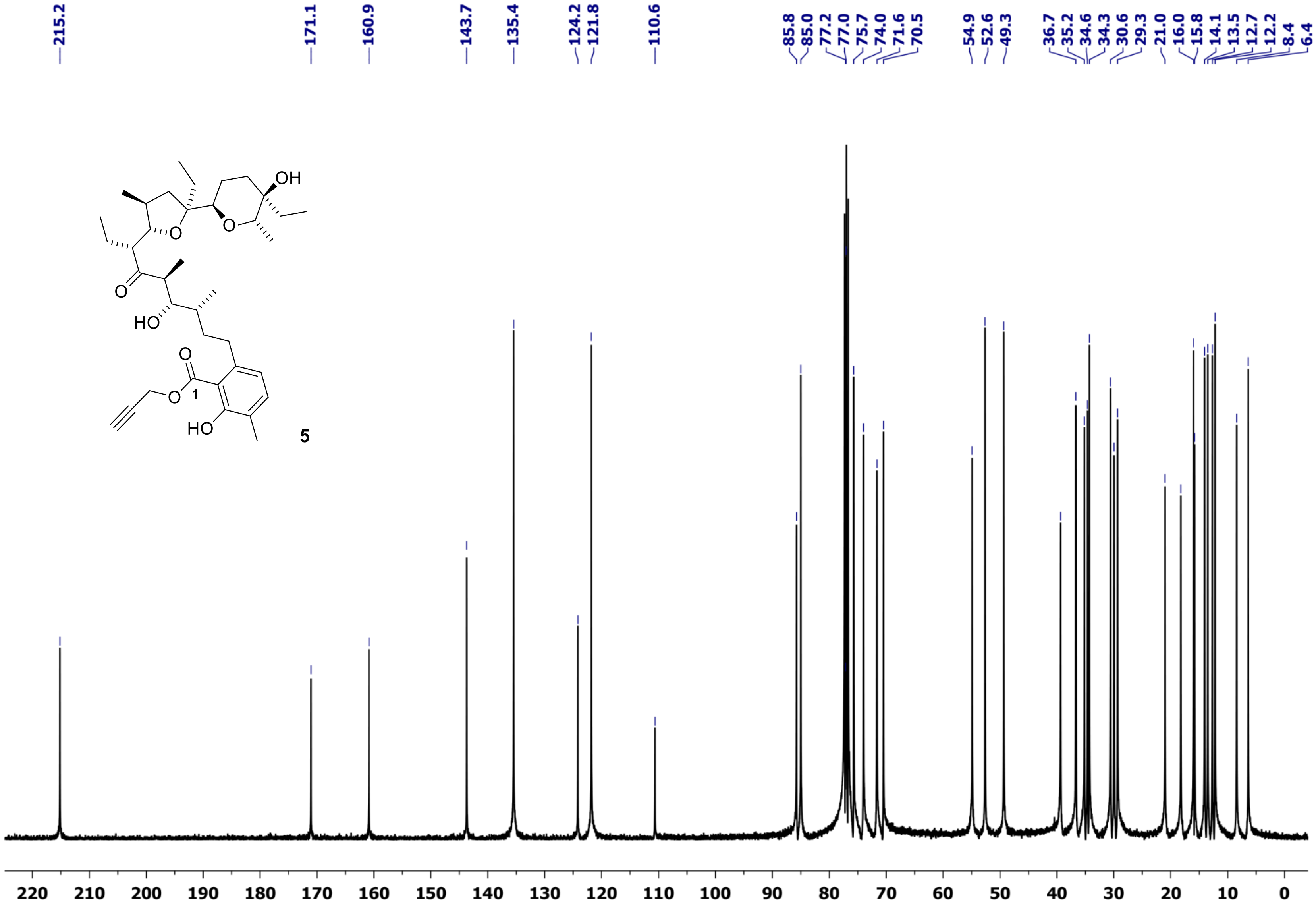

Figure S4. The ^{13}C NMR spectrum of **5** in chloroform-d.

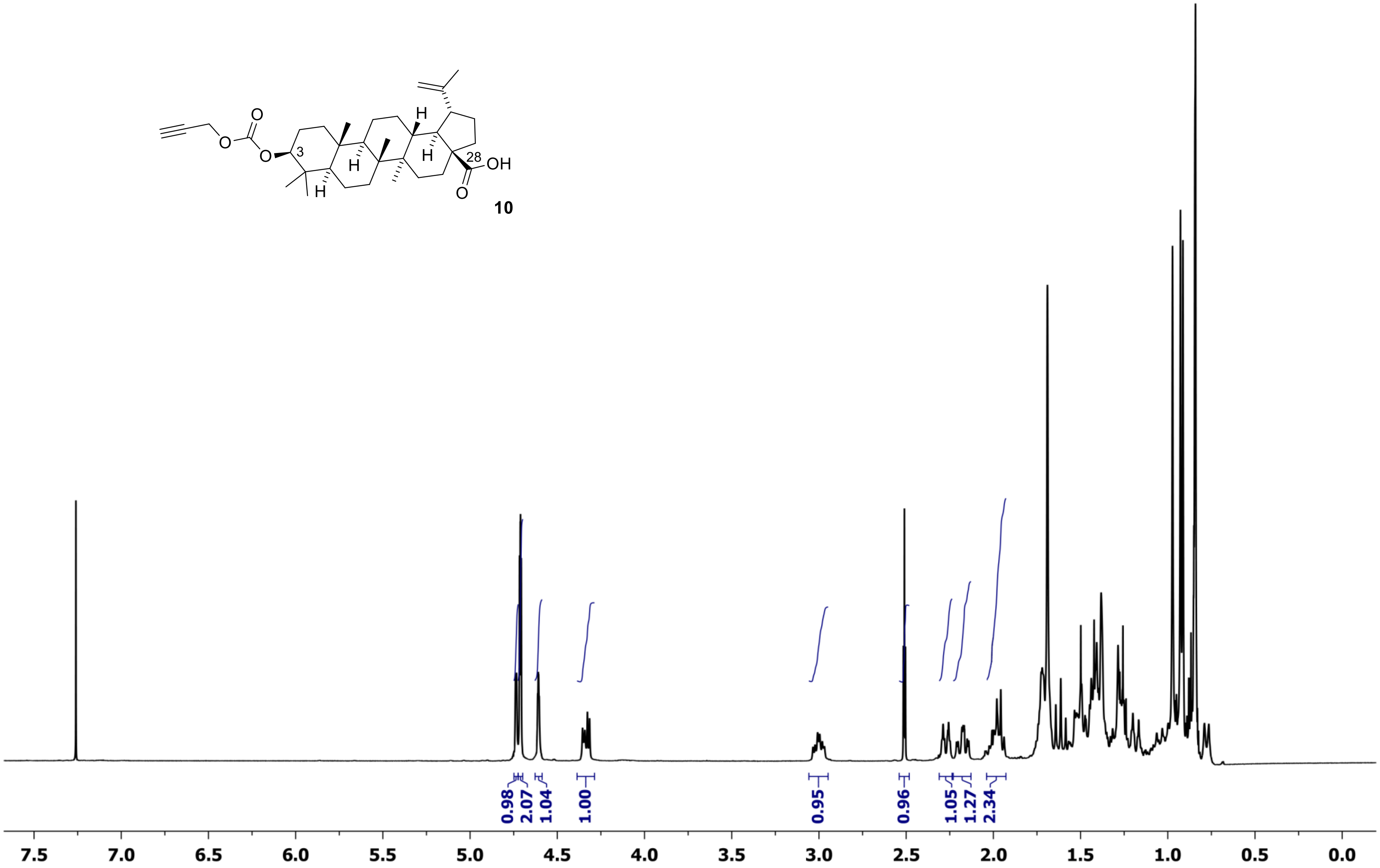

Figure S5. The ^{1}H NMR spectrum of **10** in chloroform-d.

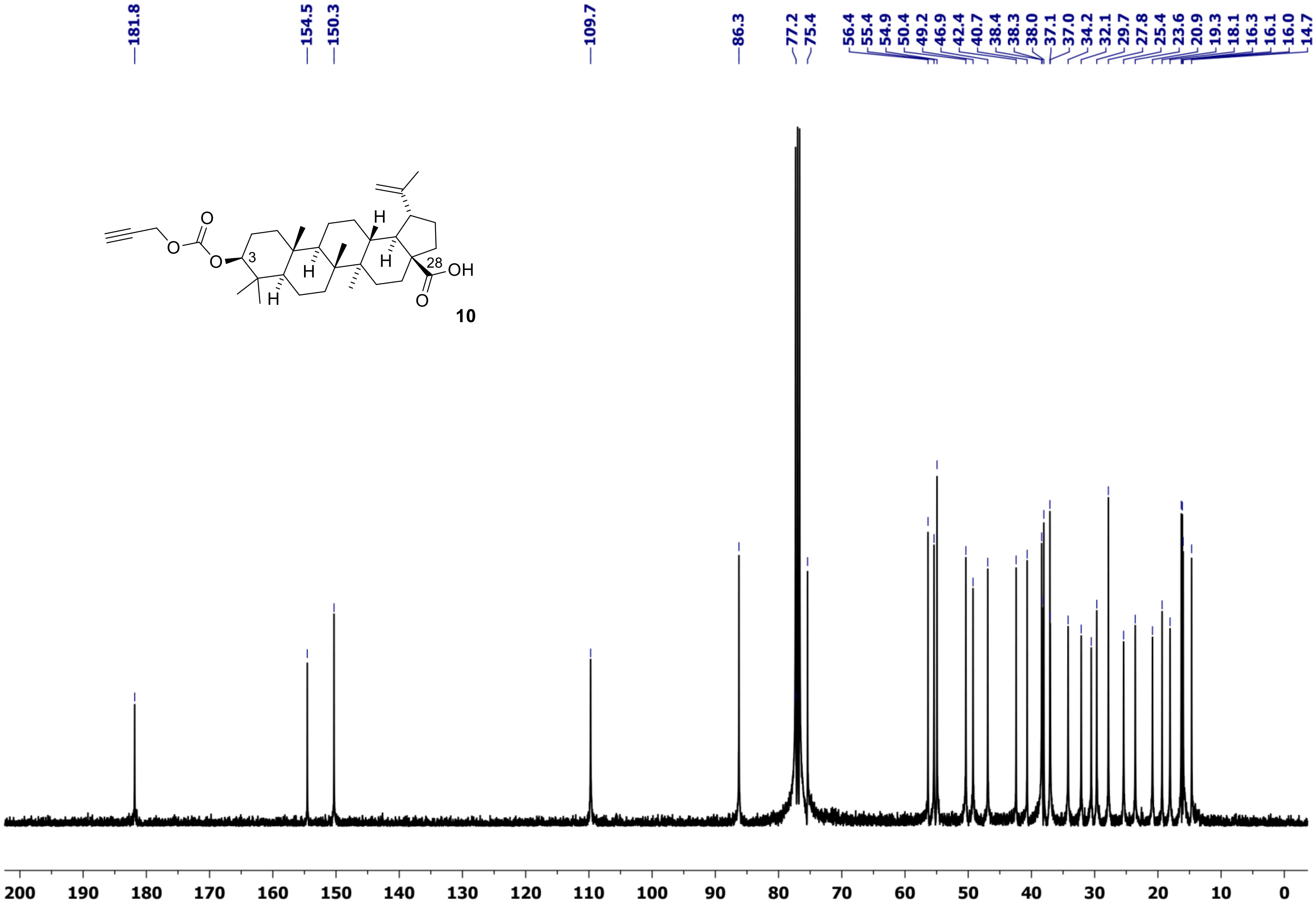

Figure S6. The ^{13}C NMR spectrum of **10** in chloroform-d.

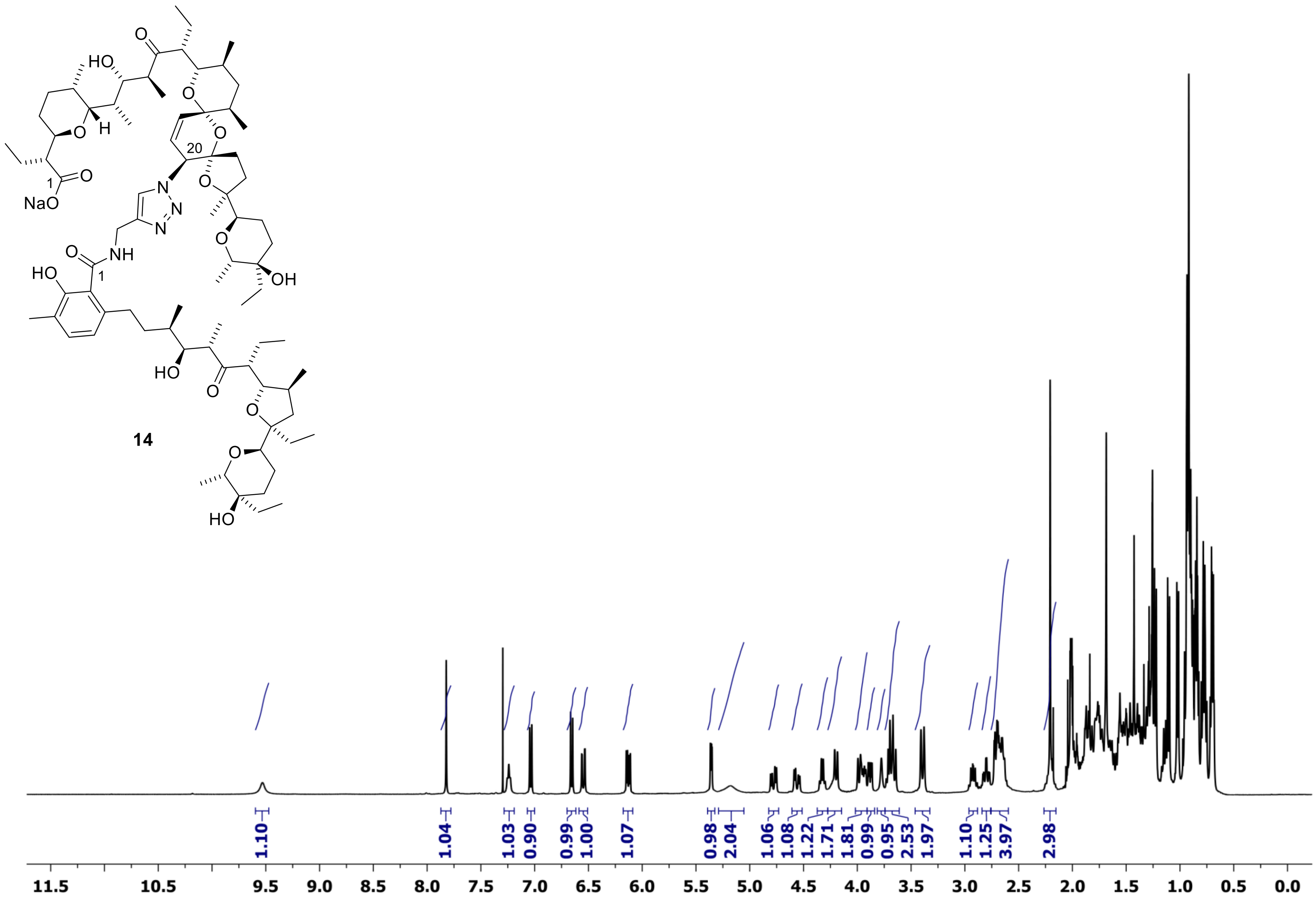

Figure S7. The ^{1}H NMR spectrum of **14** in chloroform-d.

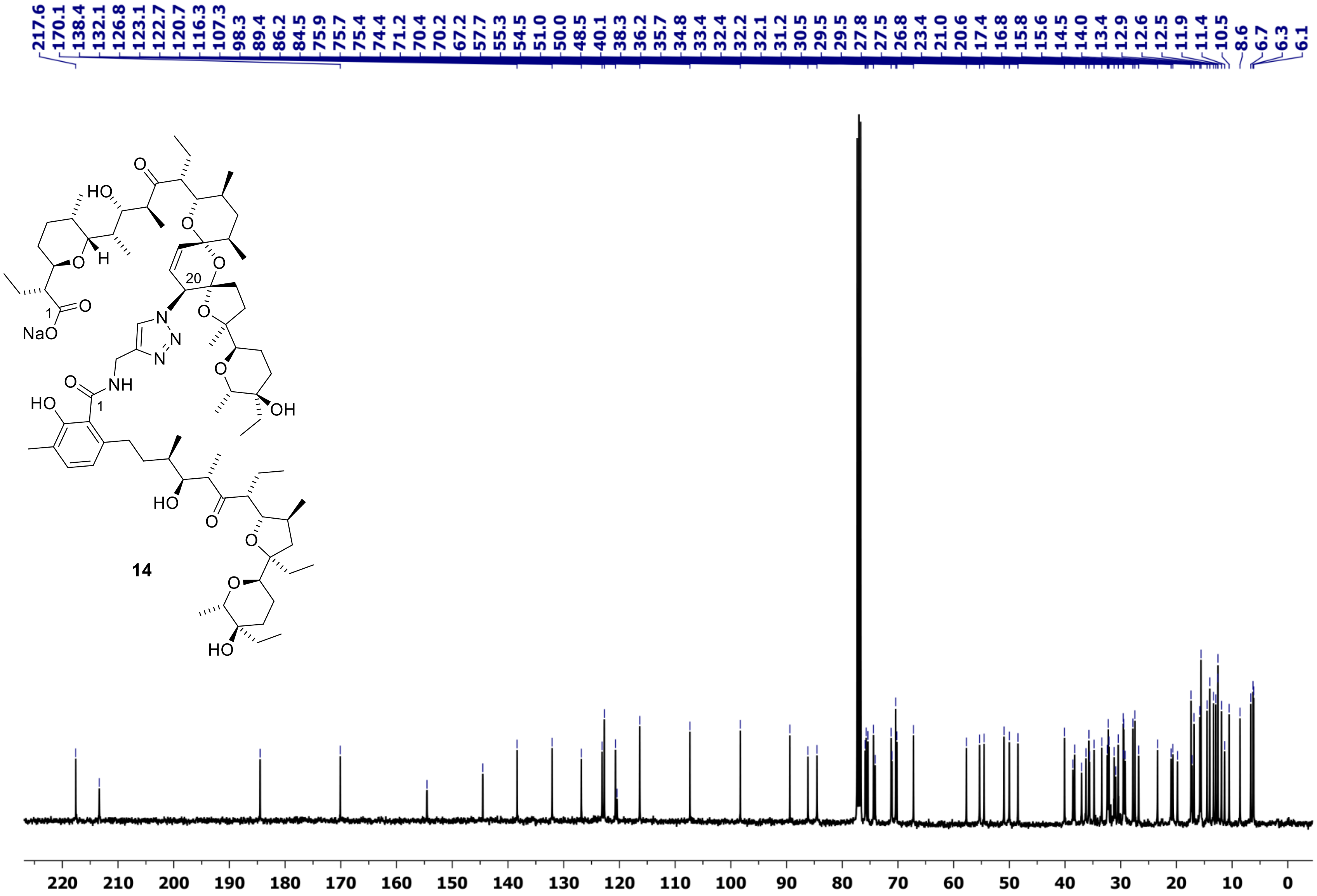

14

Figure S8. The ^{13}C NMR spectrum of **14** in chloroform-d.

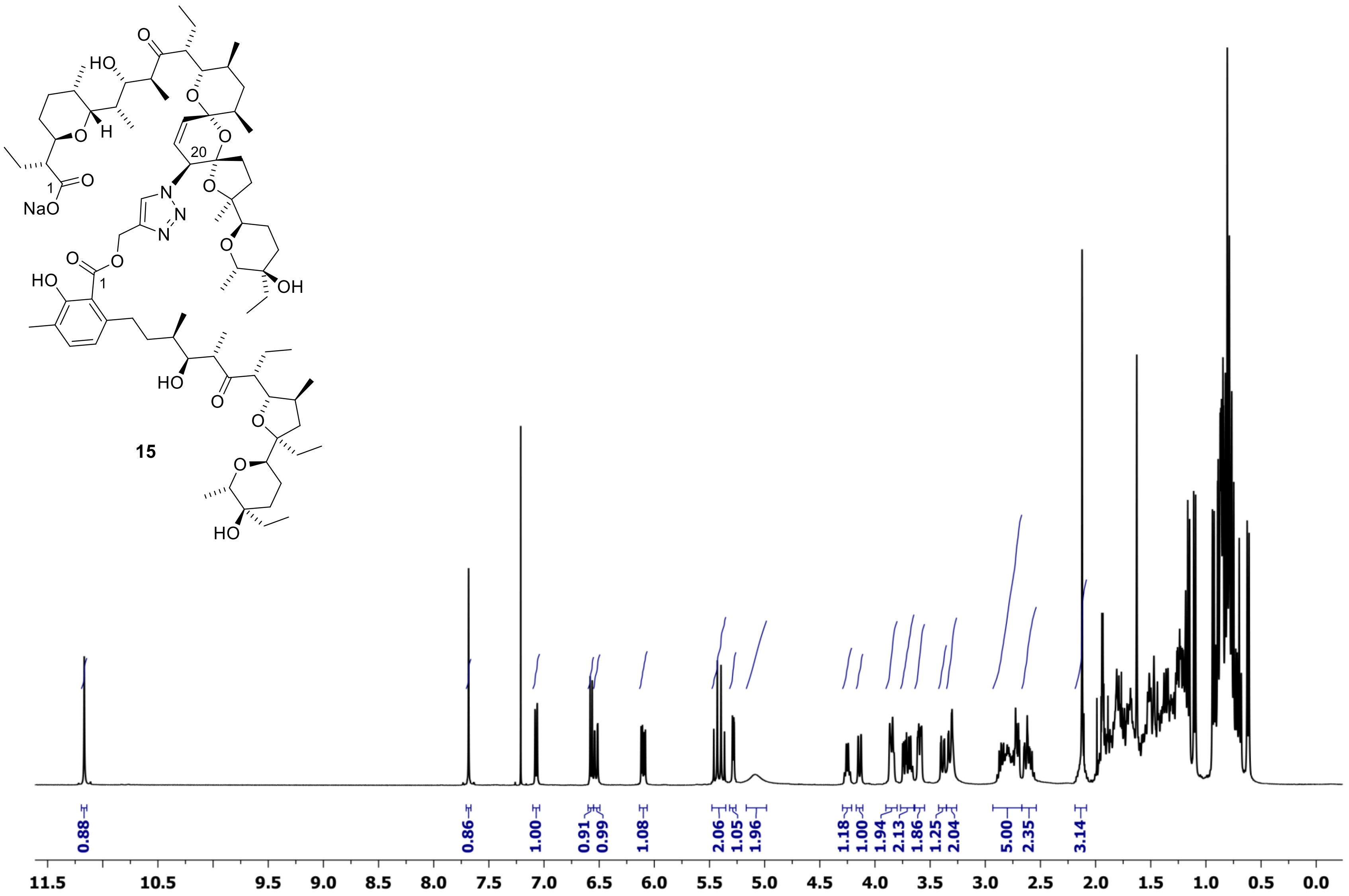

Figure S9. The ^{1}H NMR spectrum of **15** in chloroform-d.

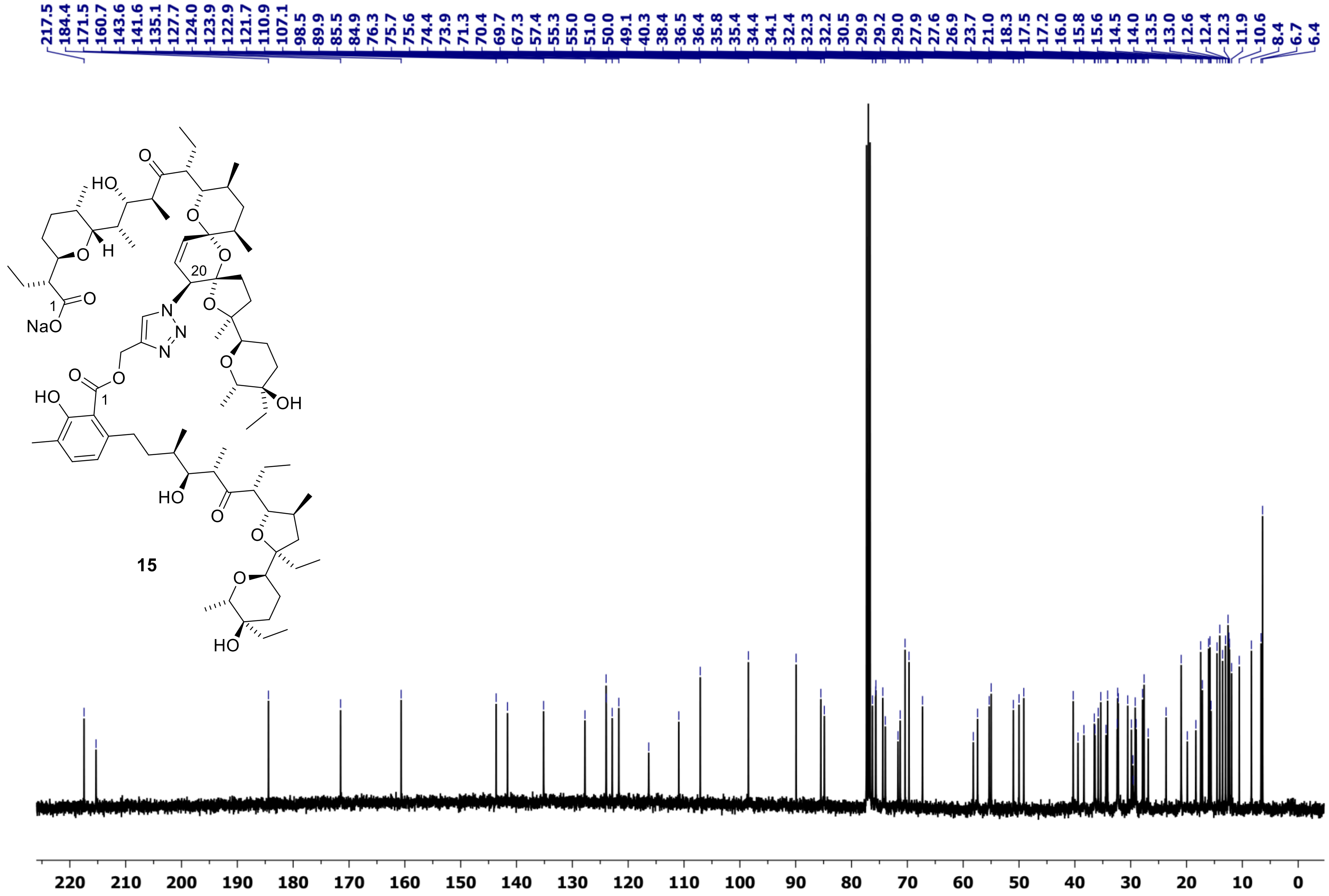

Figure S10. The ^{13}C NMR spectrum of **15** in chloroform-d.

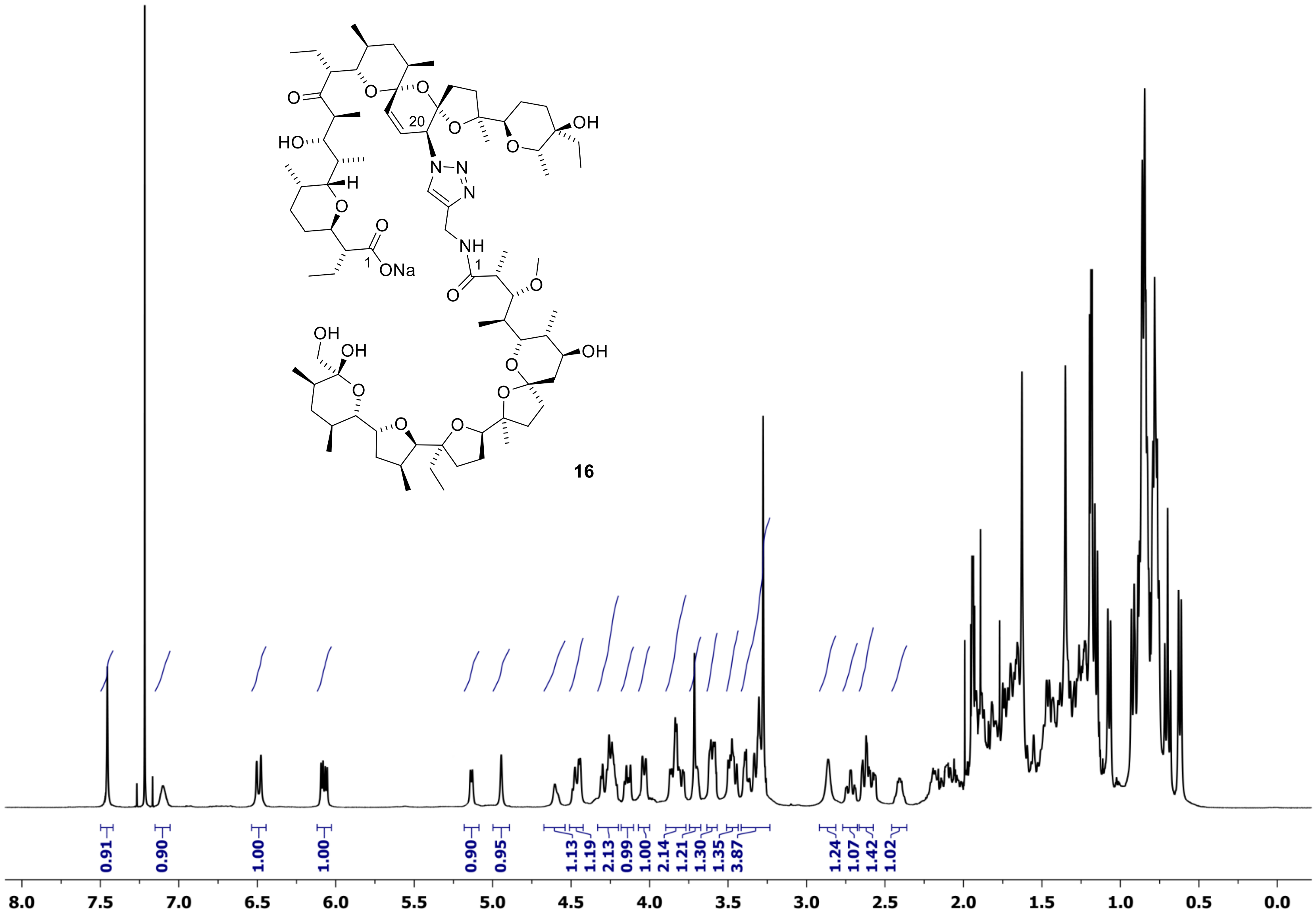

Figure S11. The ^{1}H NMR spectrum of **16** in chloroform-d.

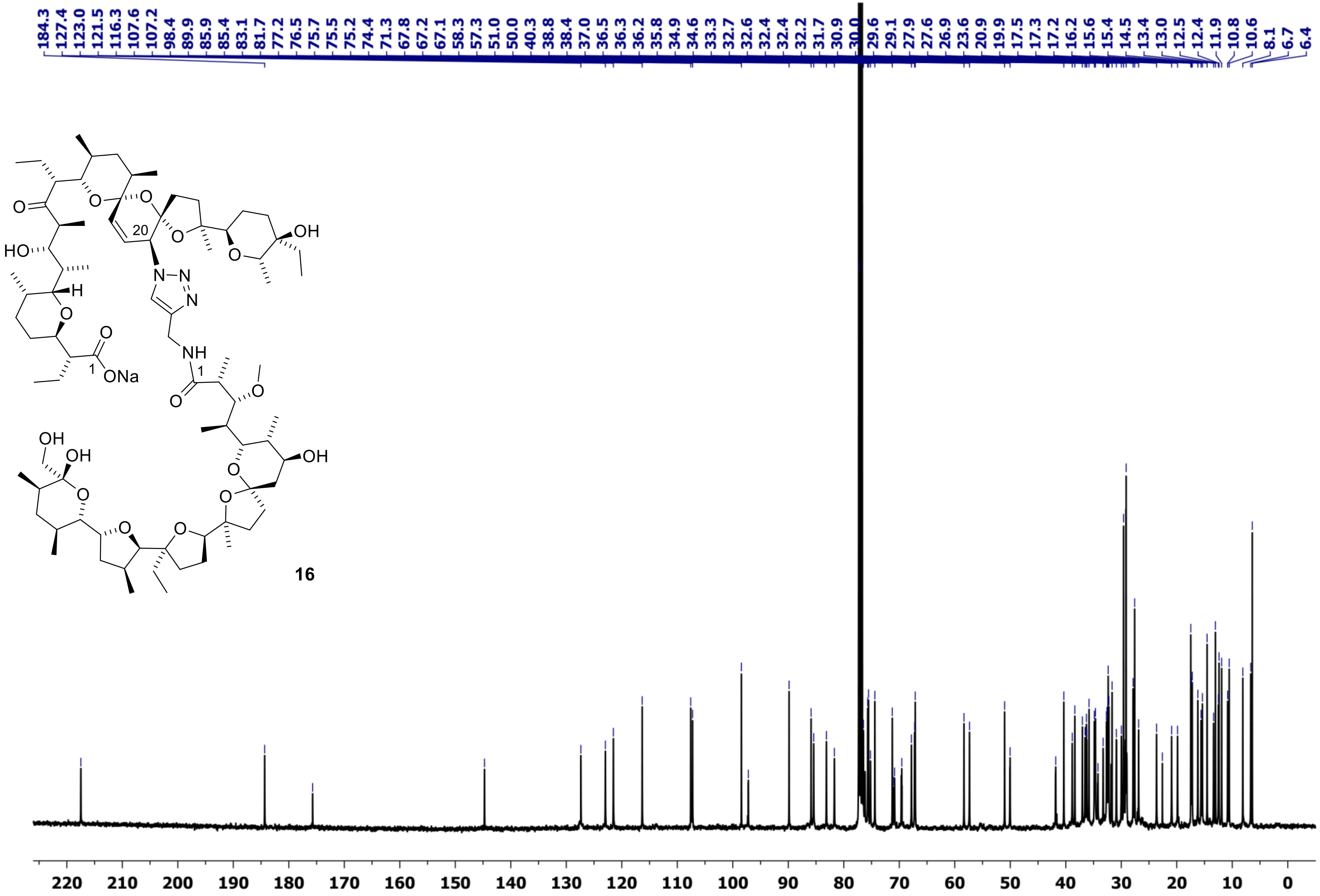

Figure S12. The ^{13}C NMR spectrum of **16** in chloroform-d.

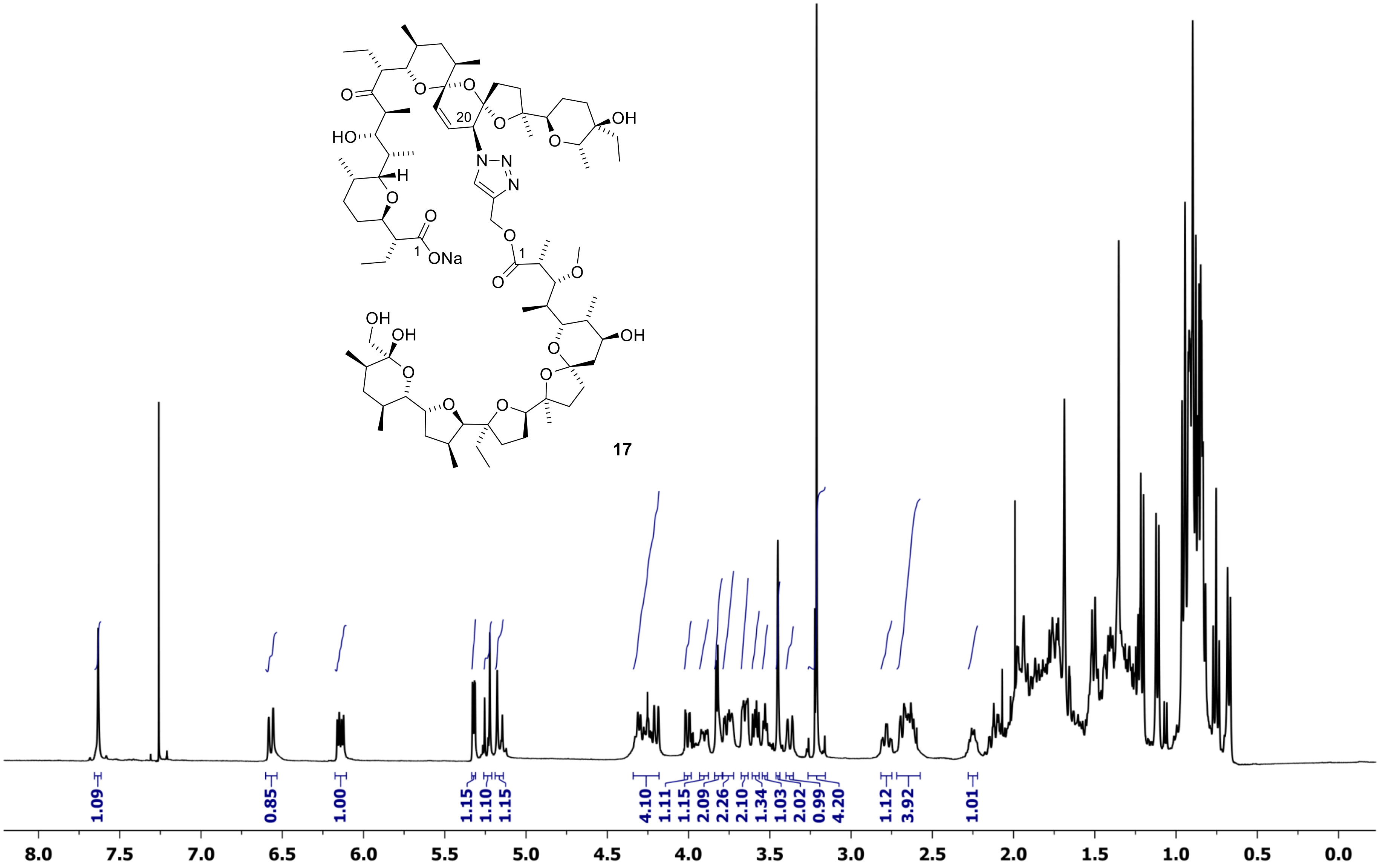

Figure S13. The ^{1}H NMR spectrum of **17** in chloroform-d.

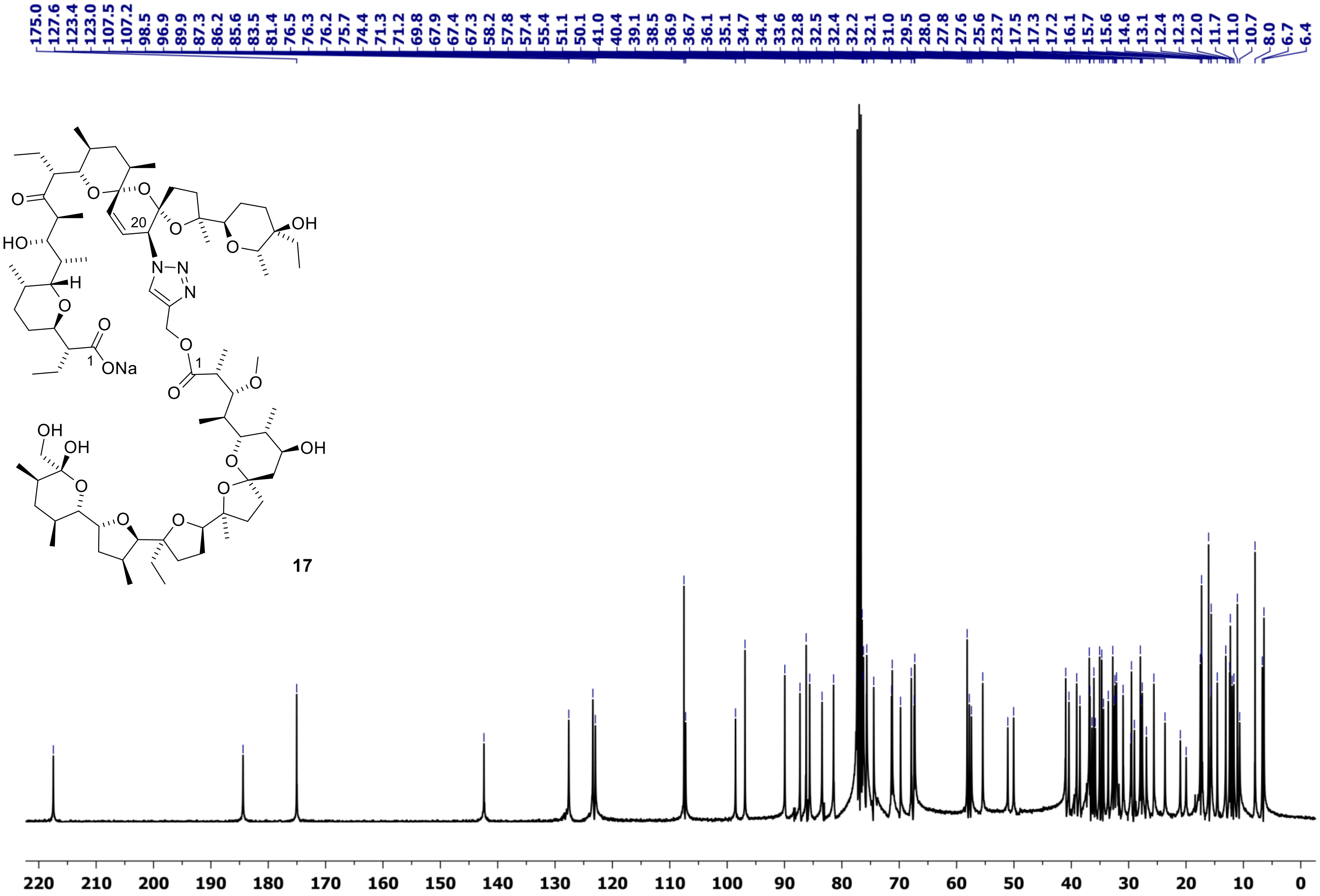

Figure S14. The ^{13}C NMR spectrum of **17** in chloroform-d.

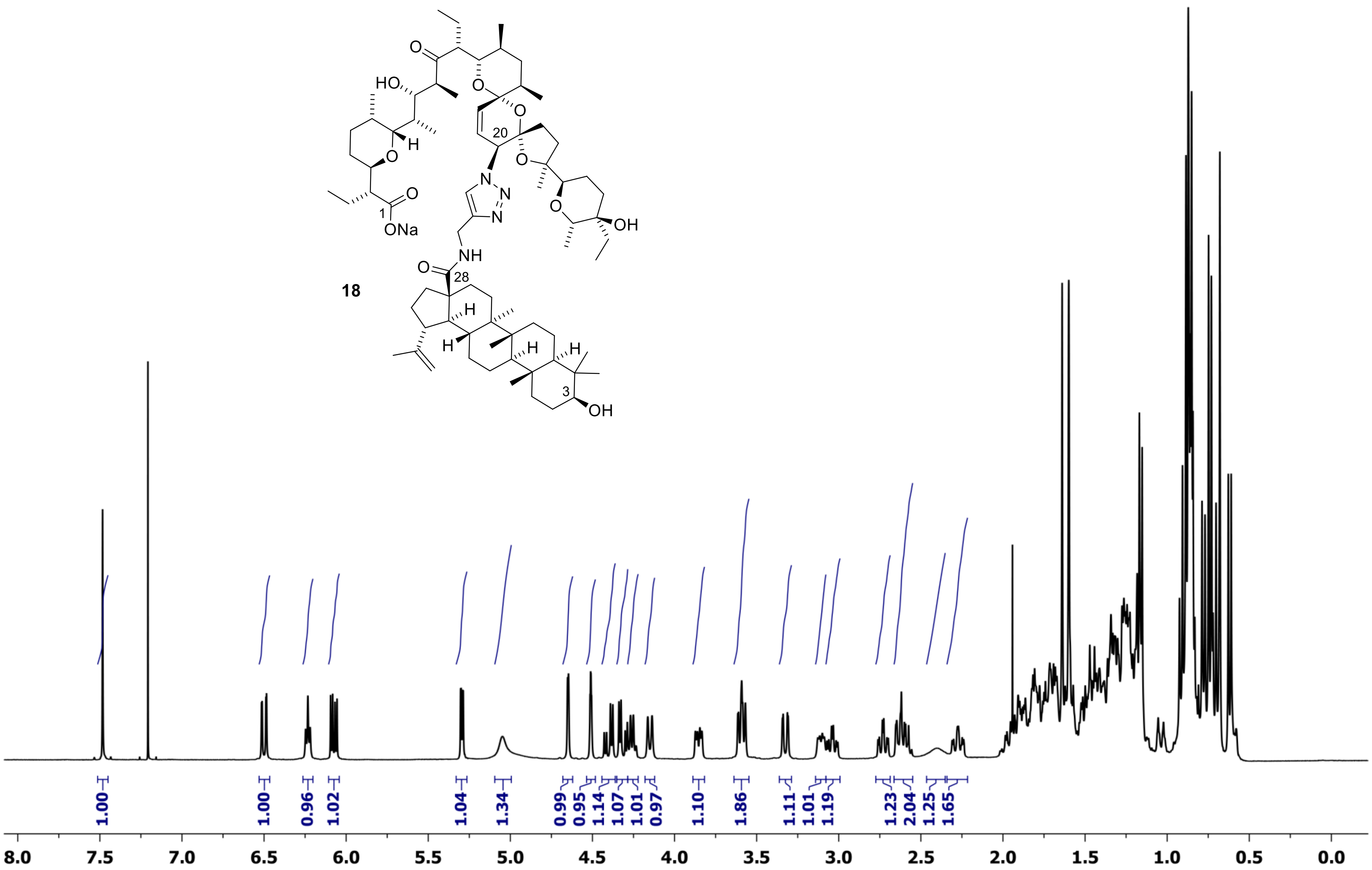

Figure S15. The ^{1}H NMR spectrum of **18** in chloroform-d.

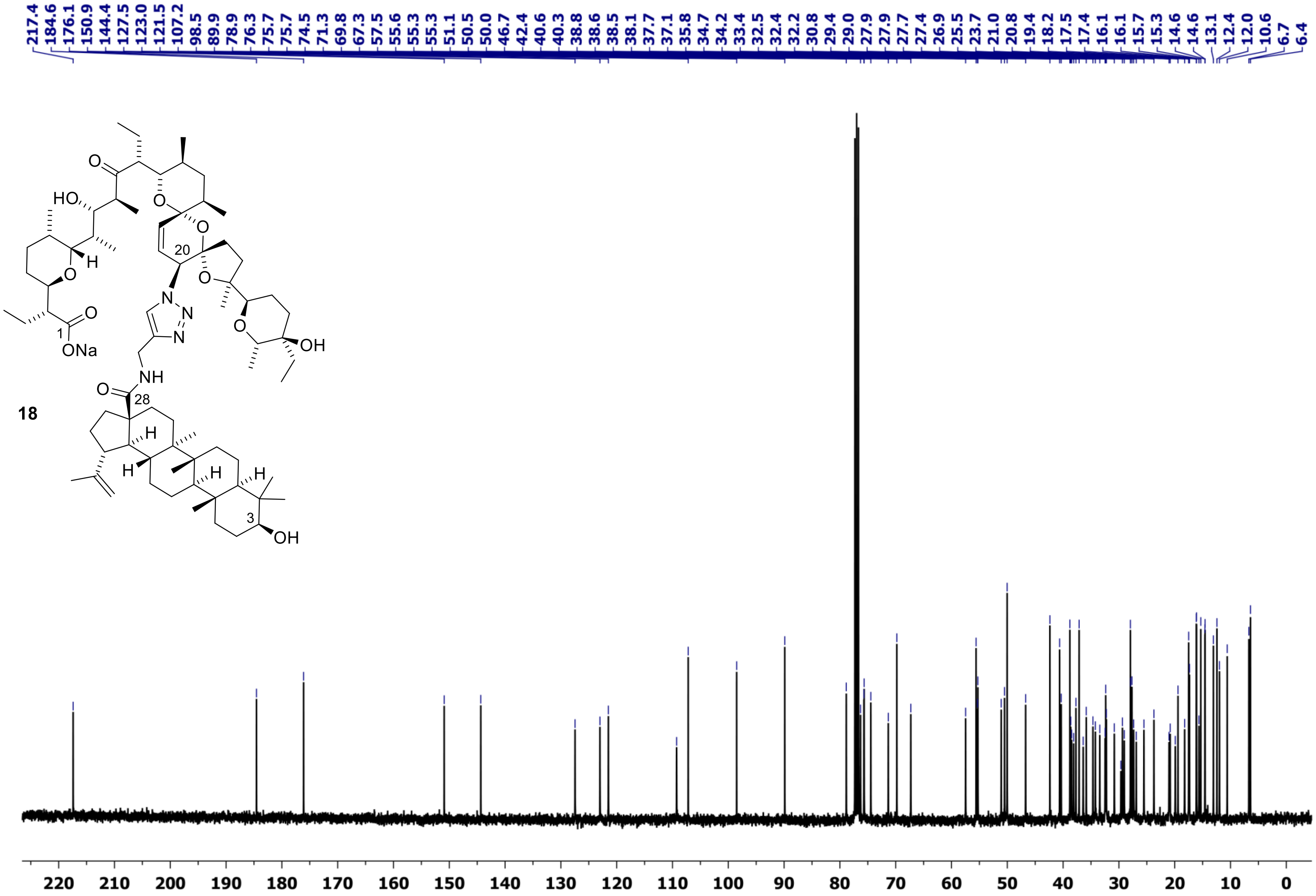

Figure S16. The ^{13}C NMR spectrum of **18** in chloroform-d.

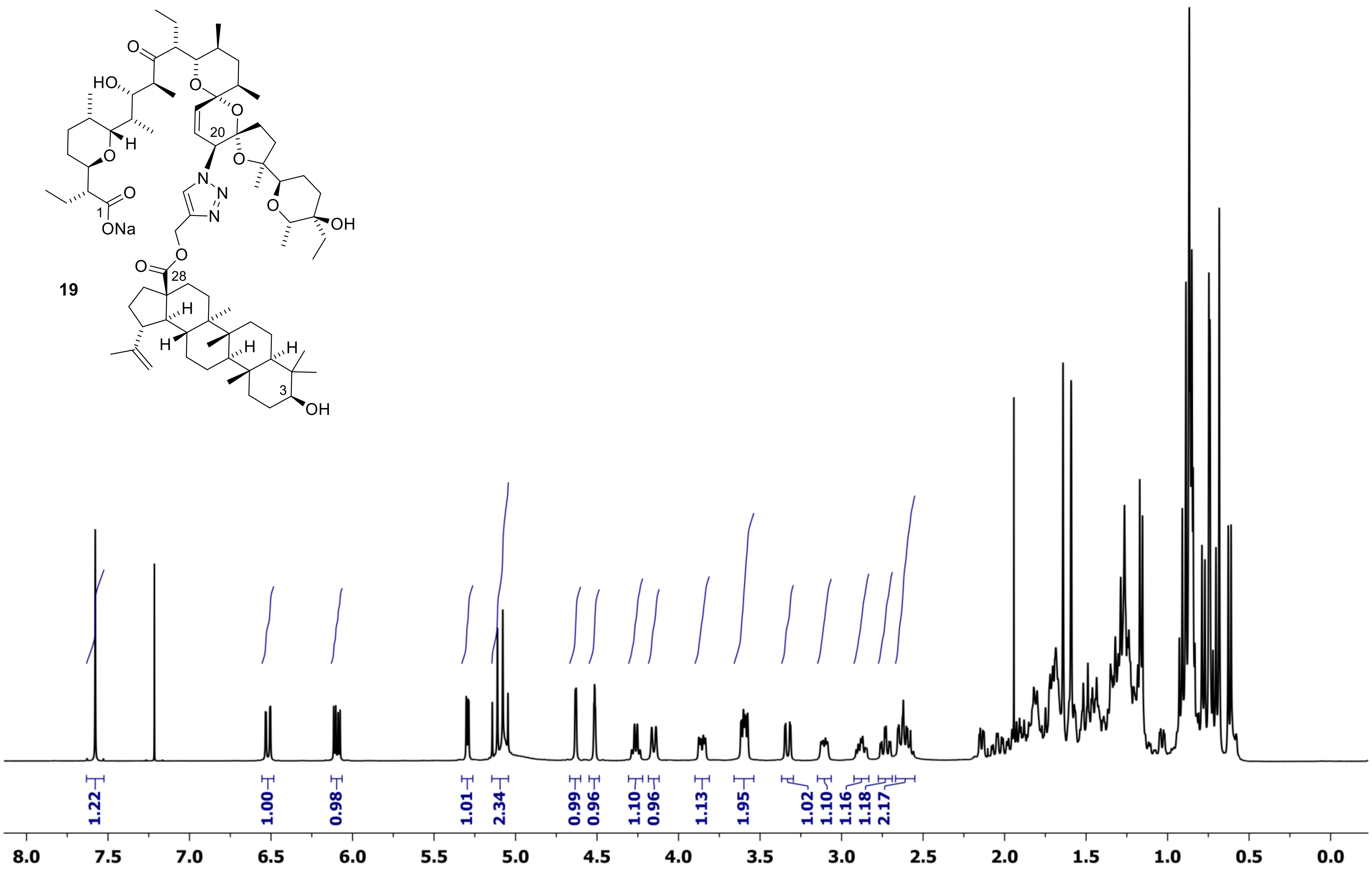

Figure S17. The ^{1}H NMR spectrum of **19** in chloroform-d.

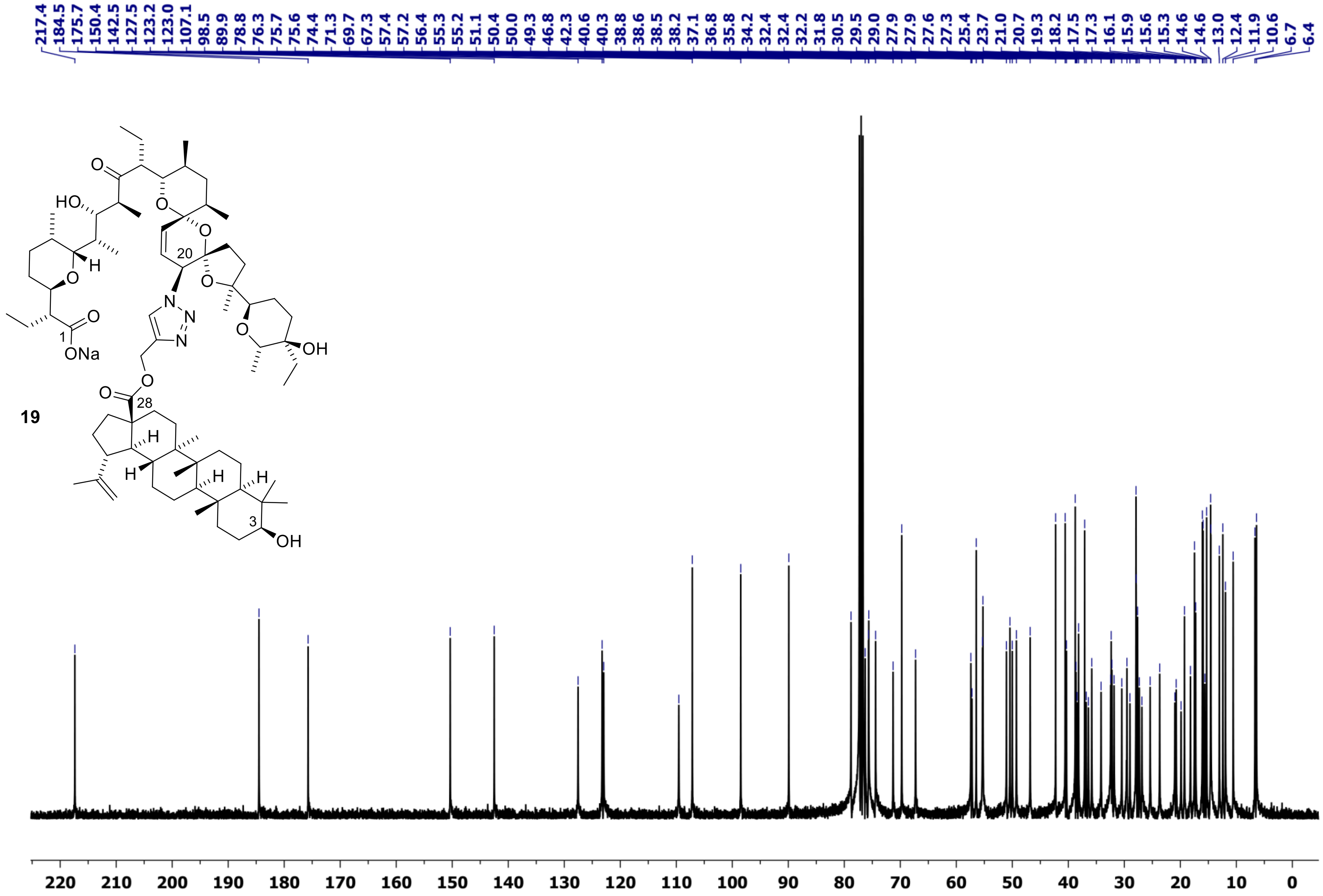

Figure S18. The ^{13}C NMR spectrum of **19** in chloroform-d.

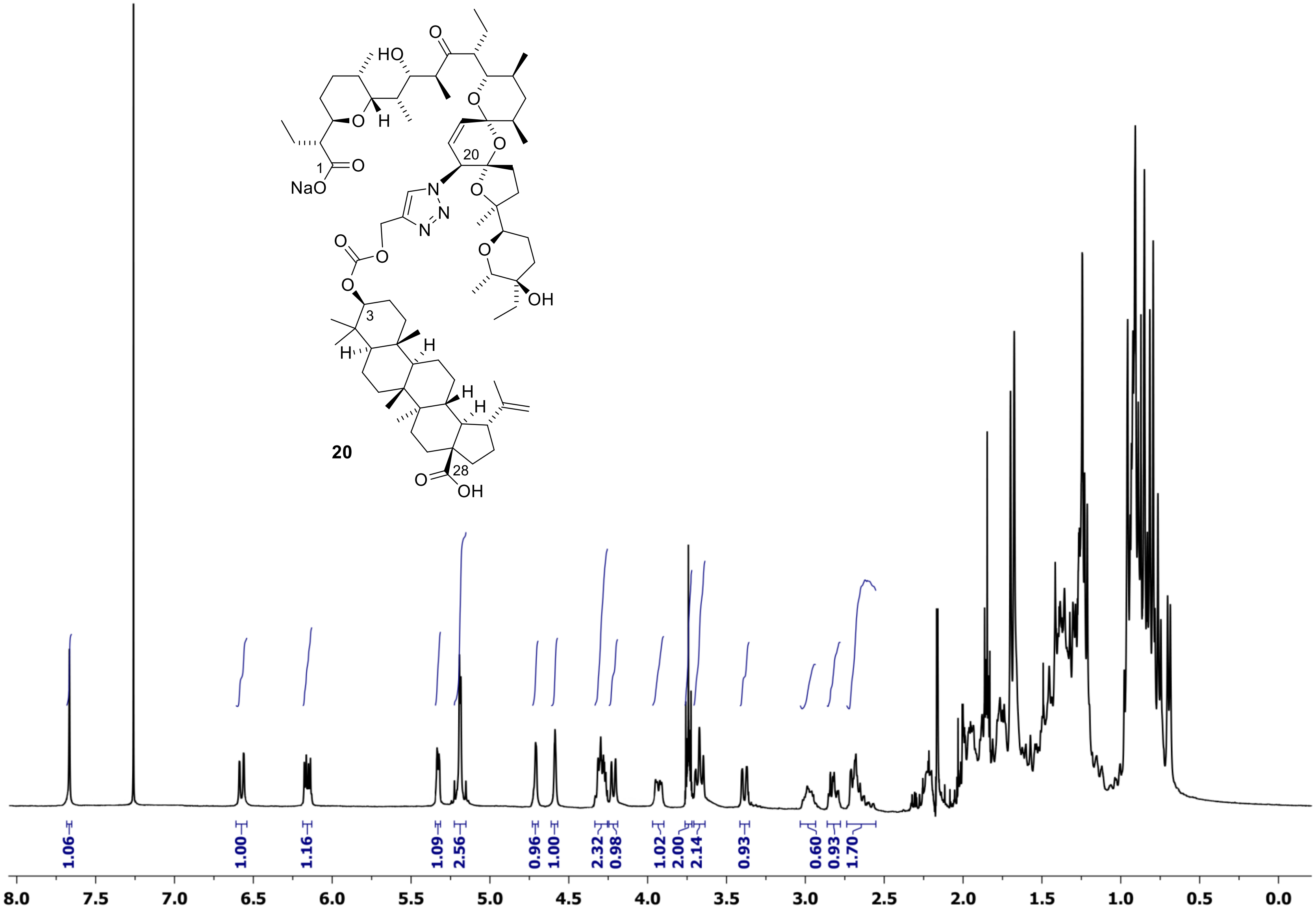

Figure S19. The ^{1}H NMR spectrum of **20** in chloroform-d.

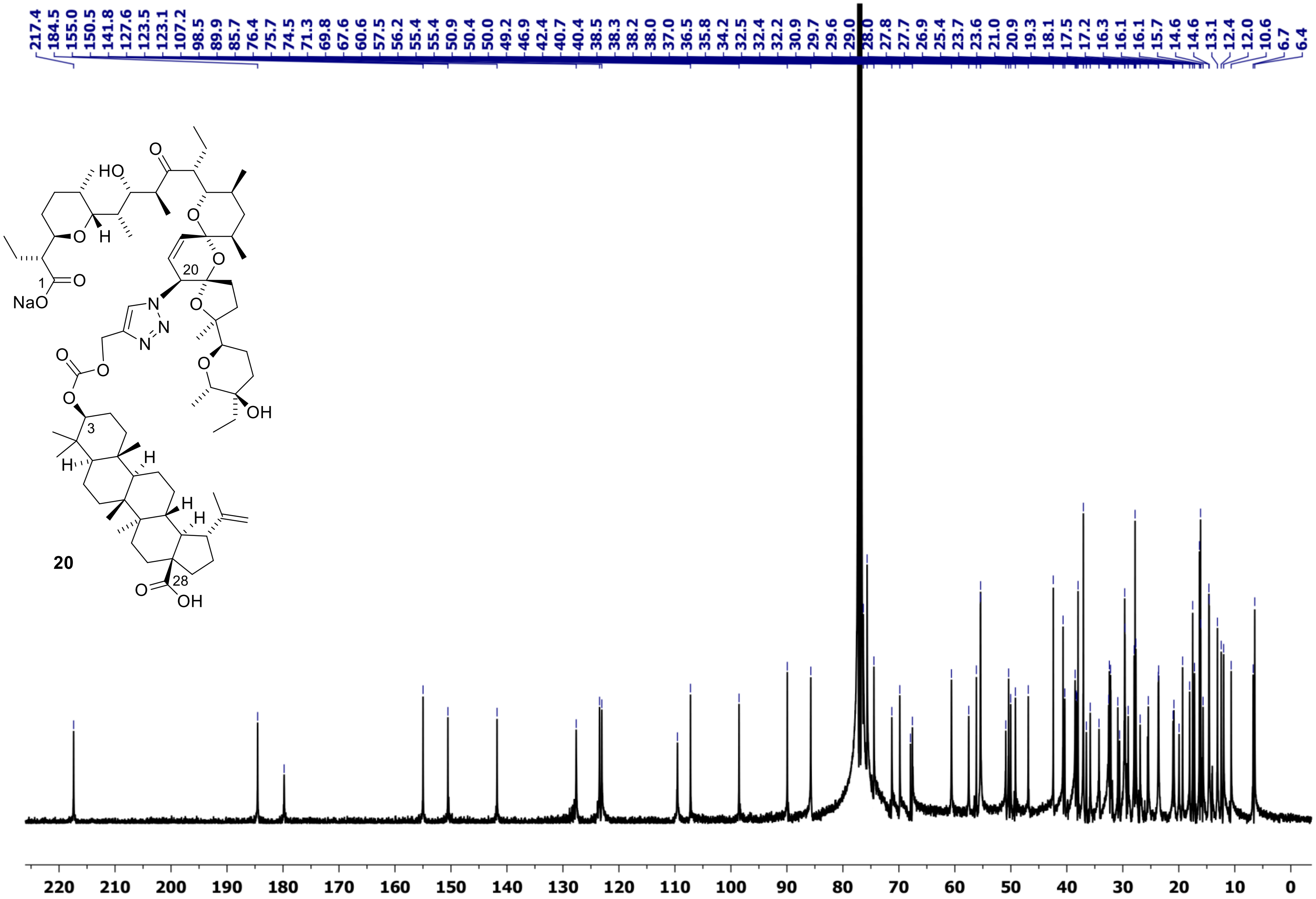

Figure S20. The ^{13}C NMR spectrum of **20** in chloroform-d.

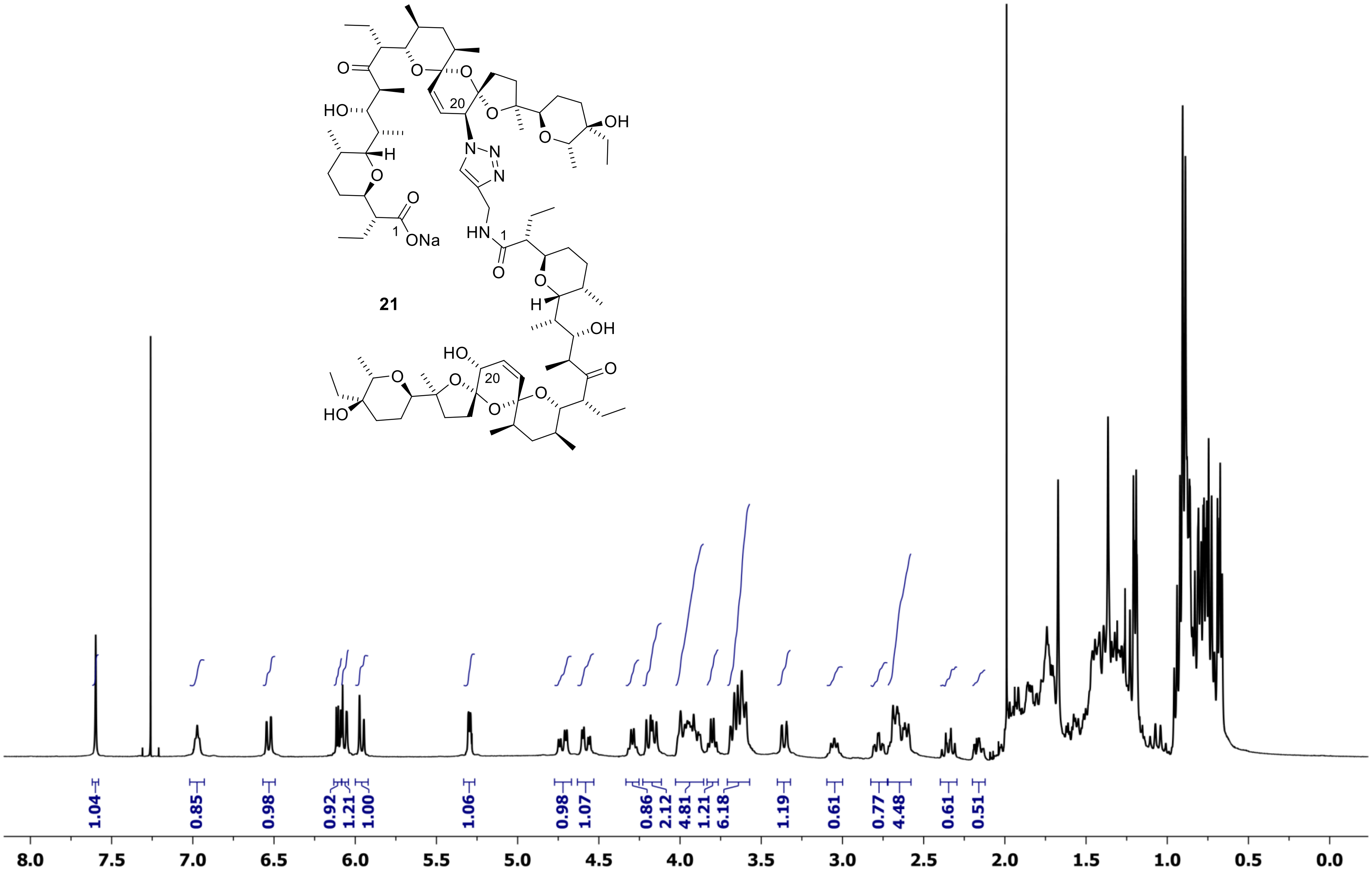

Figure S21. The ^{1}H NMR spectrum of **21** in chloroform-d.

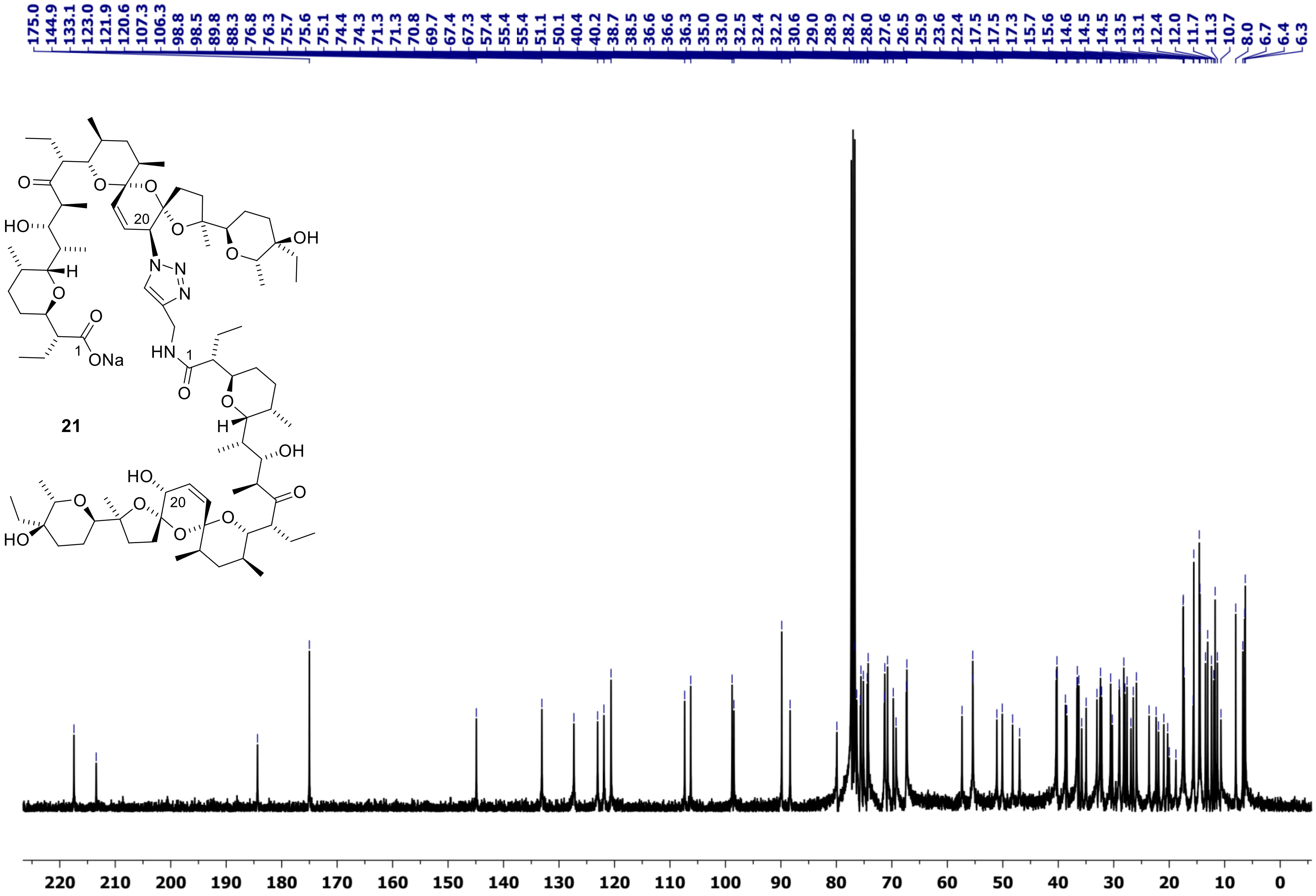

Figure S22. The ^{13}C NMR spectrum of **21** in chloroform-d.

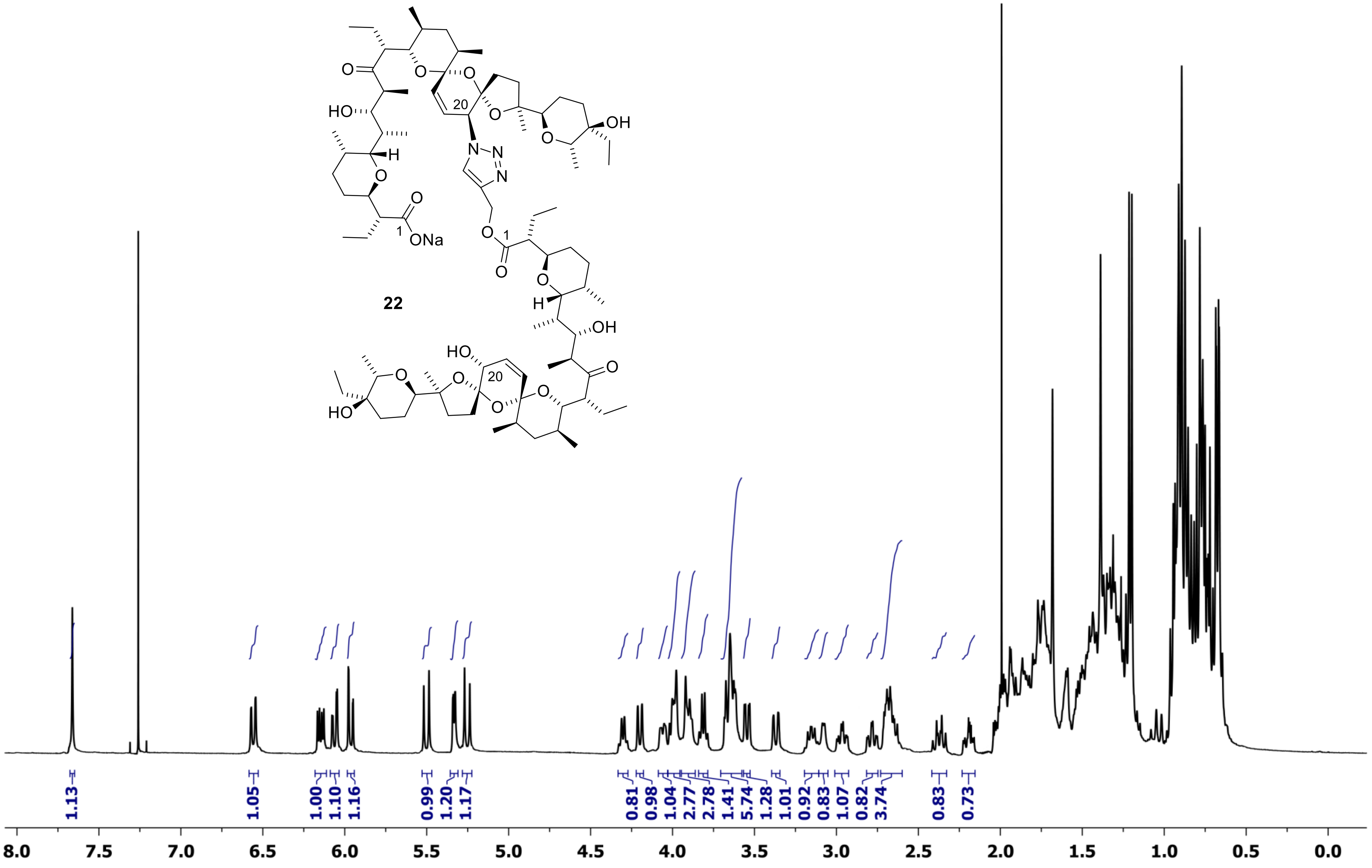

Figure S23. The ^{1}H NMR spectrum of **22** in chloroform-d.

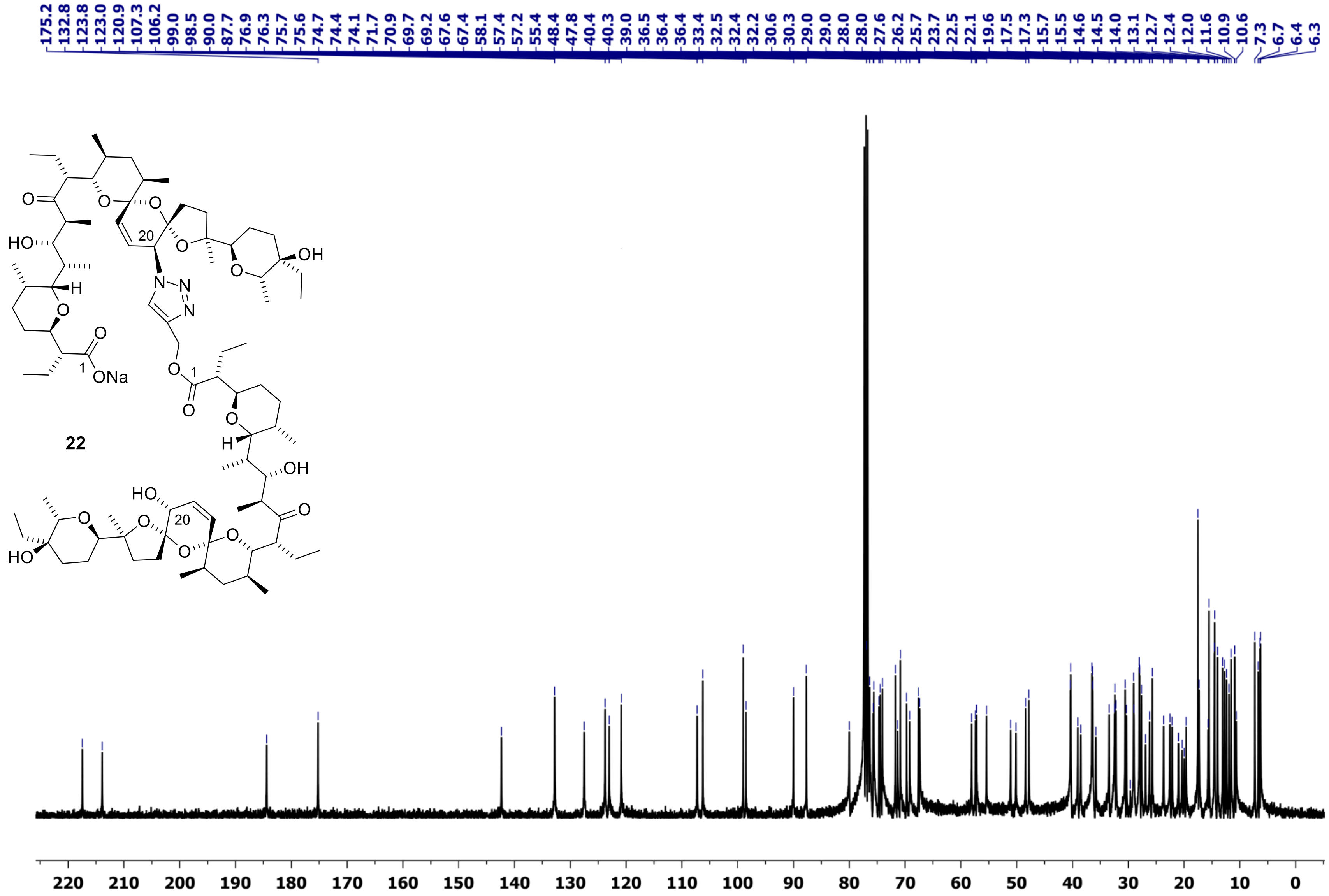

Figure S24. The ^{13}C NMR spectrum of **22** in chloroform-d.

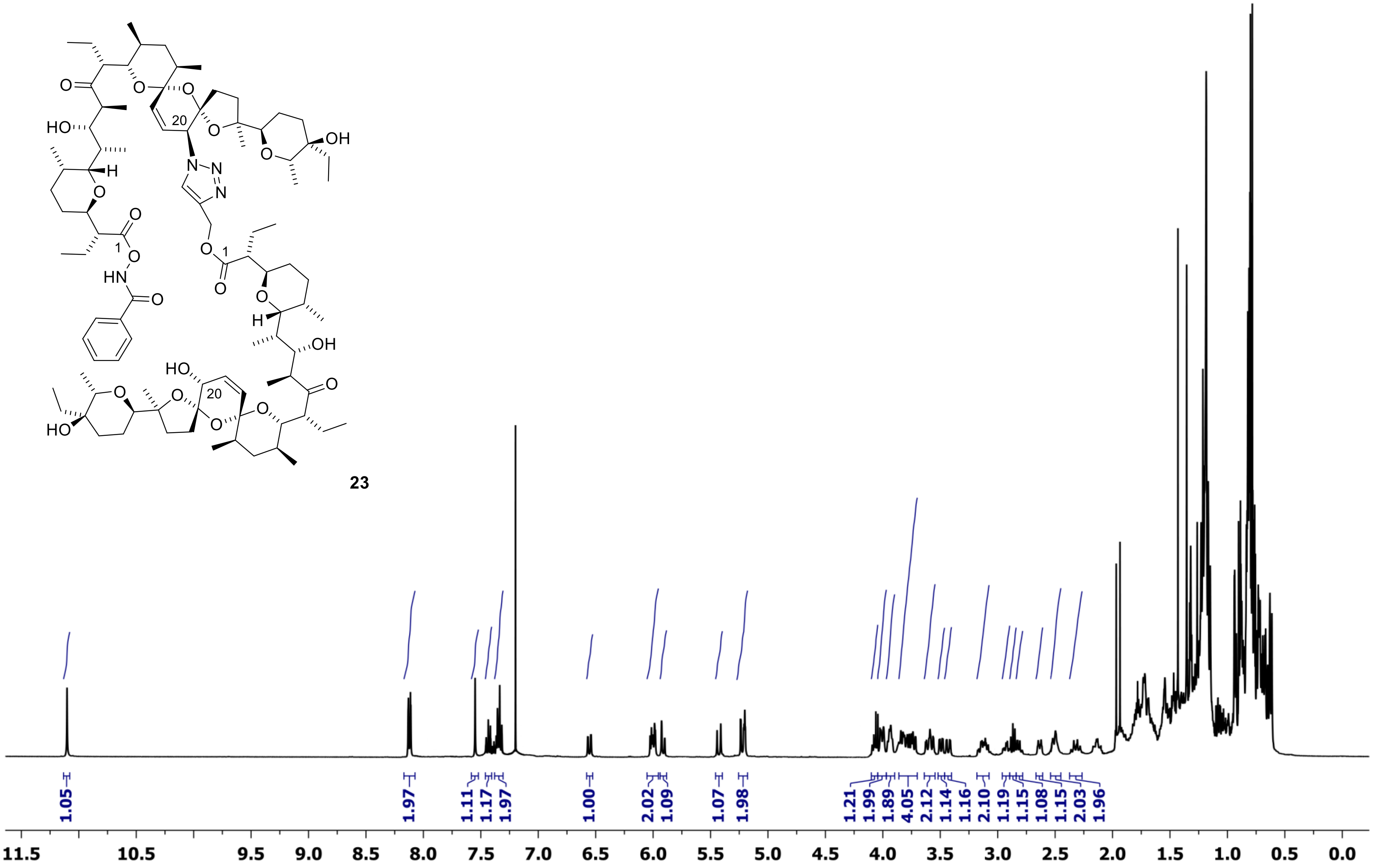

23

Figure S25. The ^{1}H NMR spectrum of **23** in chloroform-d.

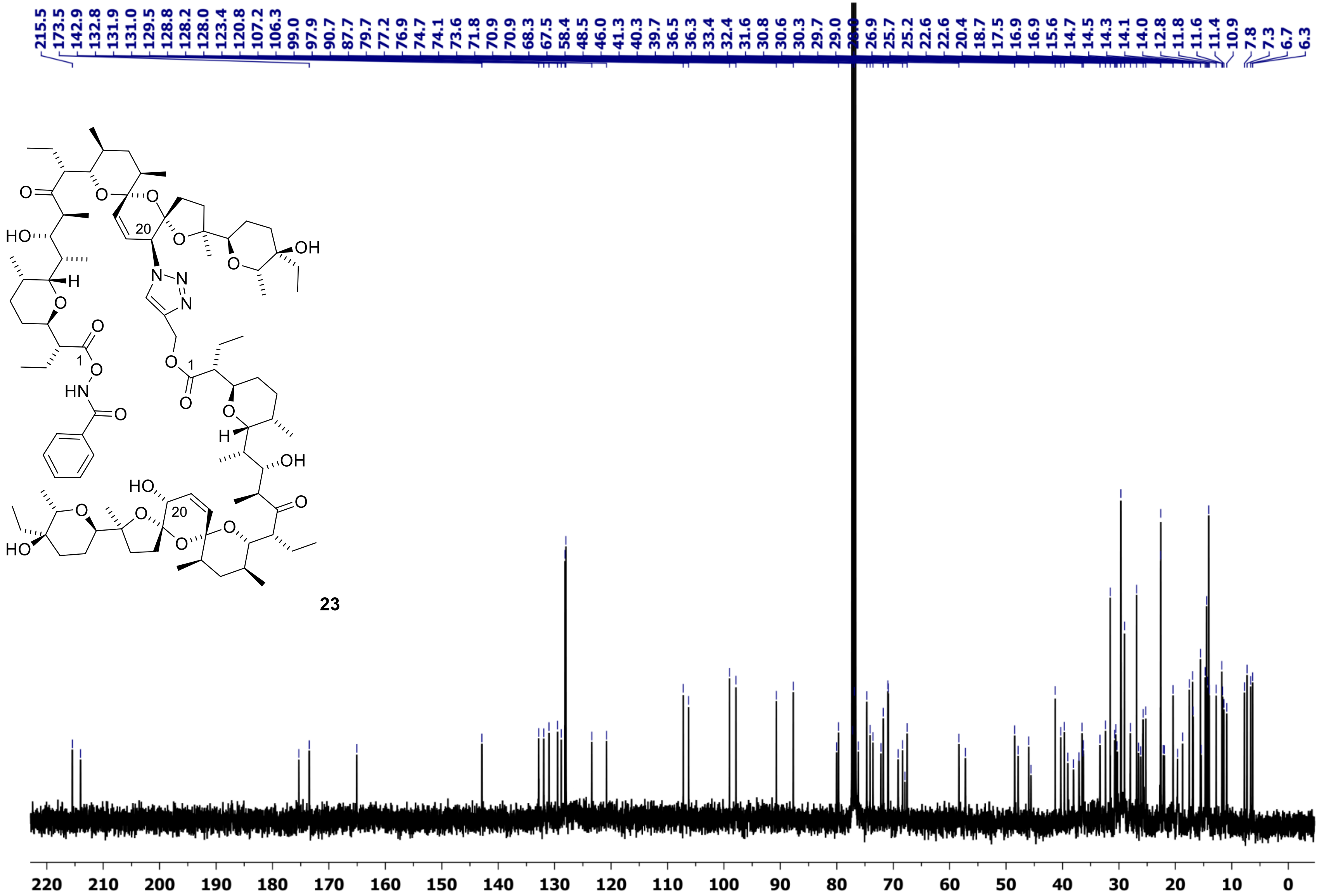

Figure S26. The ^{13}C NMR spectrum of **23** in chloroform-d.

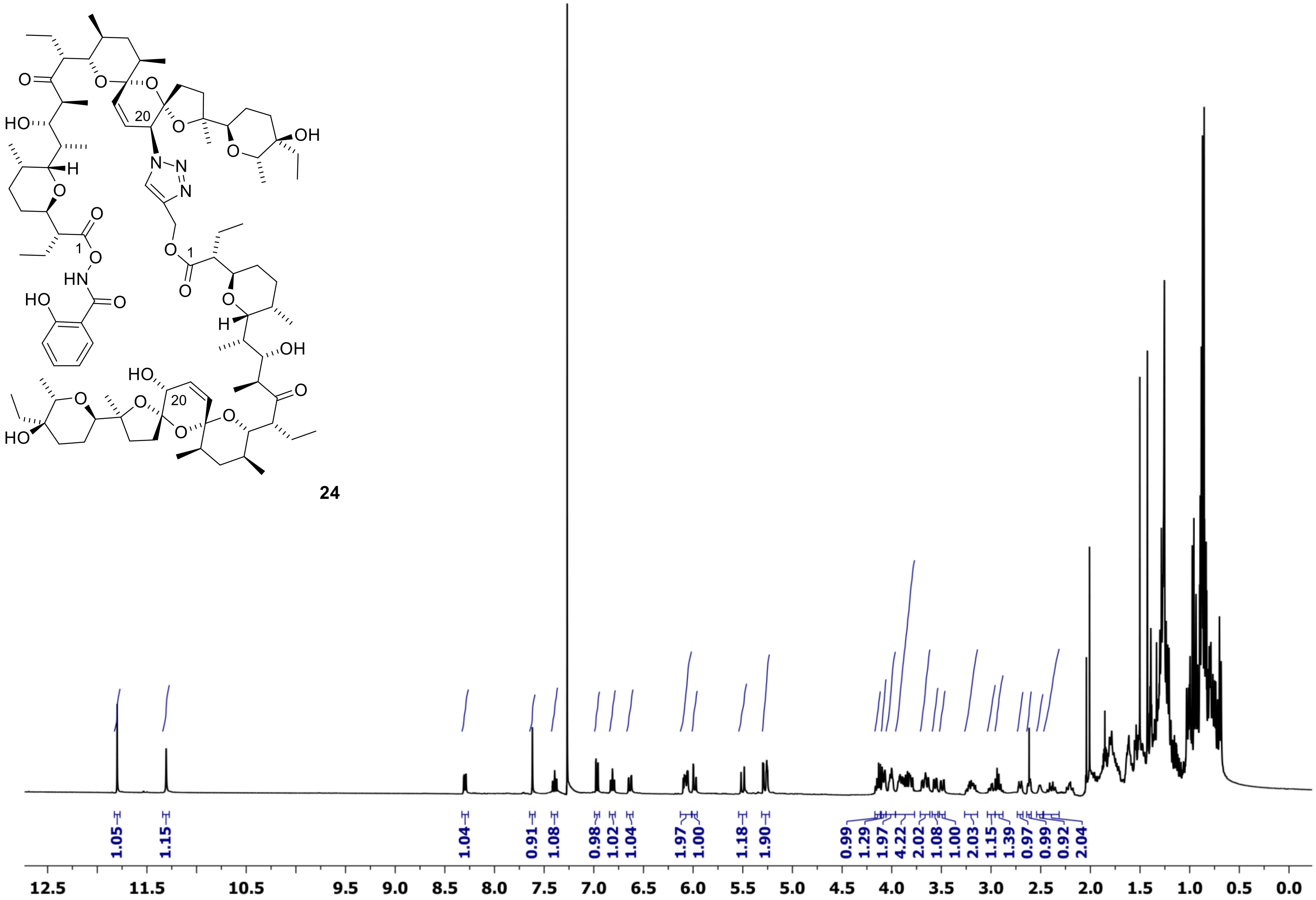

24

Figure S27. The ^{1}H NMR spectrum of **24** in chloroform-d.

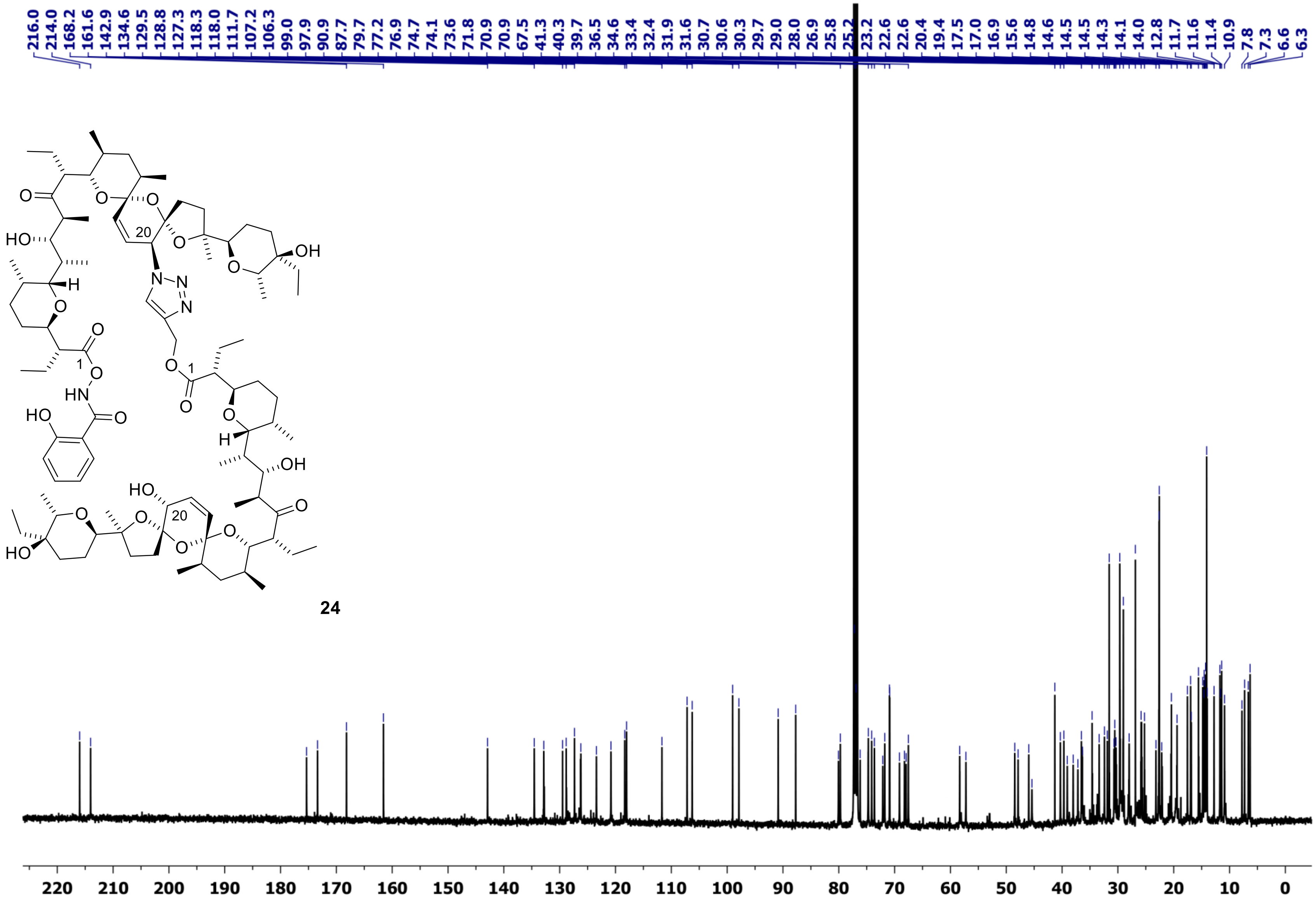

Figure S28. The ^{13}C NMR spectrum of **24** in chloroform-d.

ESI MS analysis of dimeric polyether ionophores

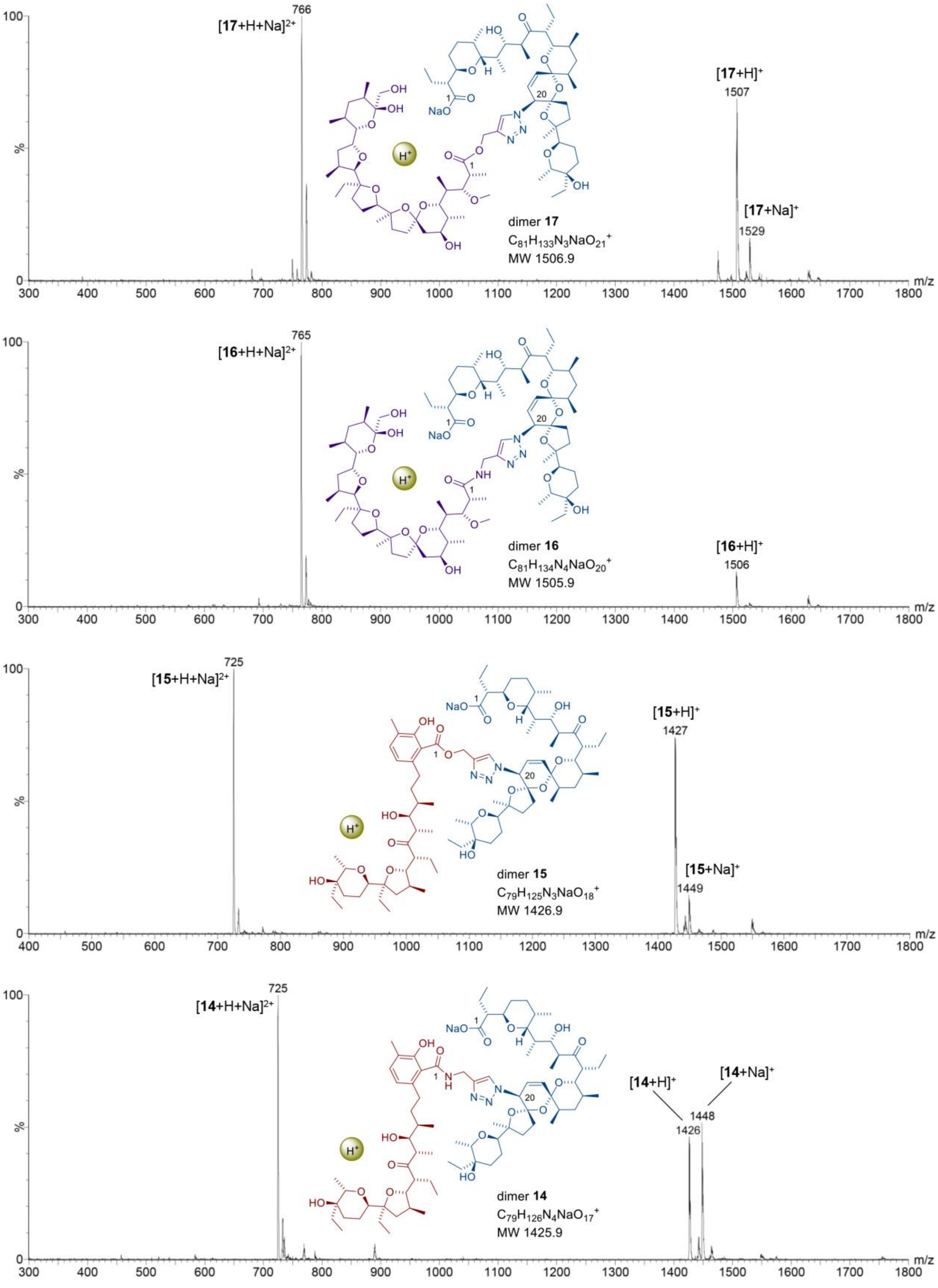

Figure S29. The ESI mass spectra of a mixture of **14**, **15**, **16** and **17** with $NaClO_4$ at cv = 30 V.

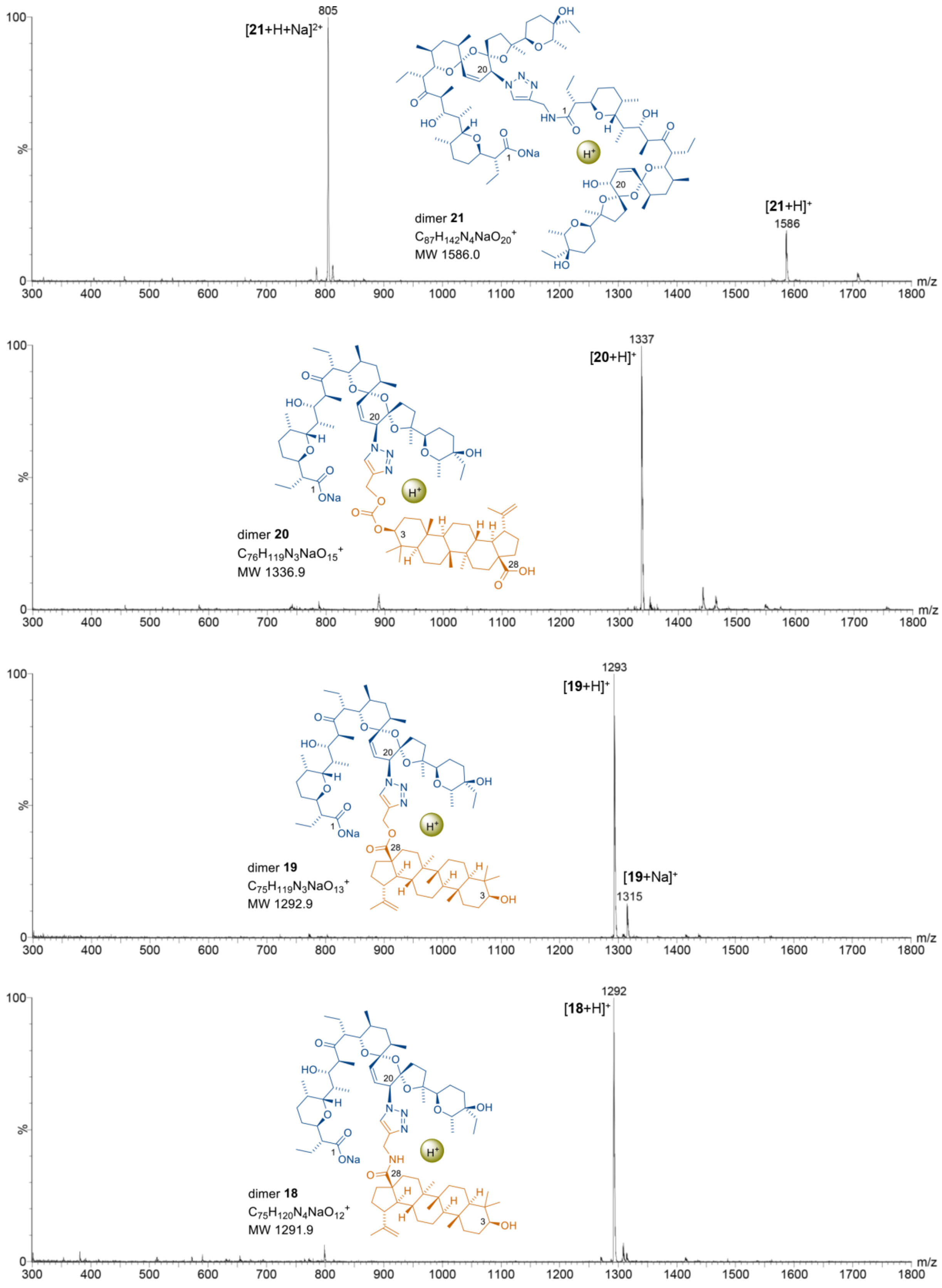

Figure S30. The ESI mass spectra of a mixture of **18**, **19**, **20** and **21** with $NaClO_4$ at cv = 30 V.

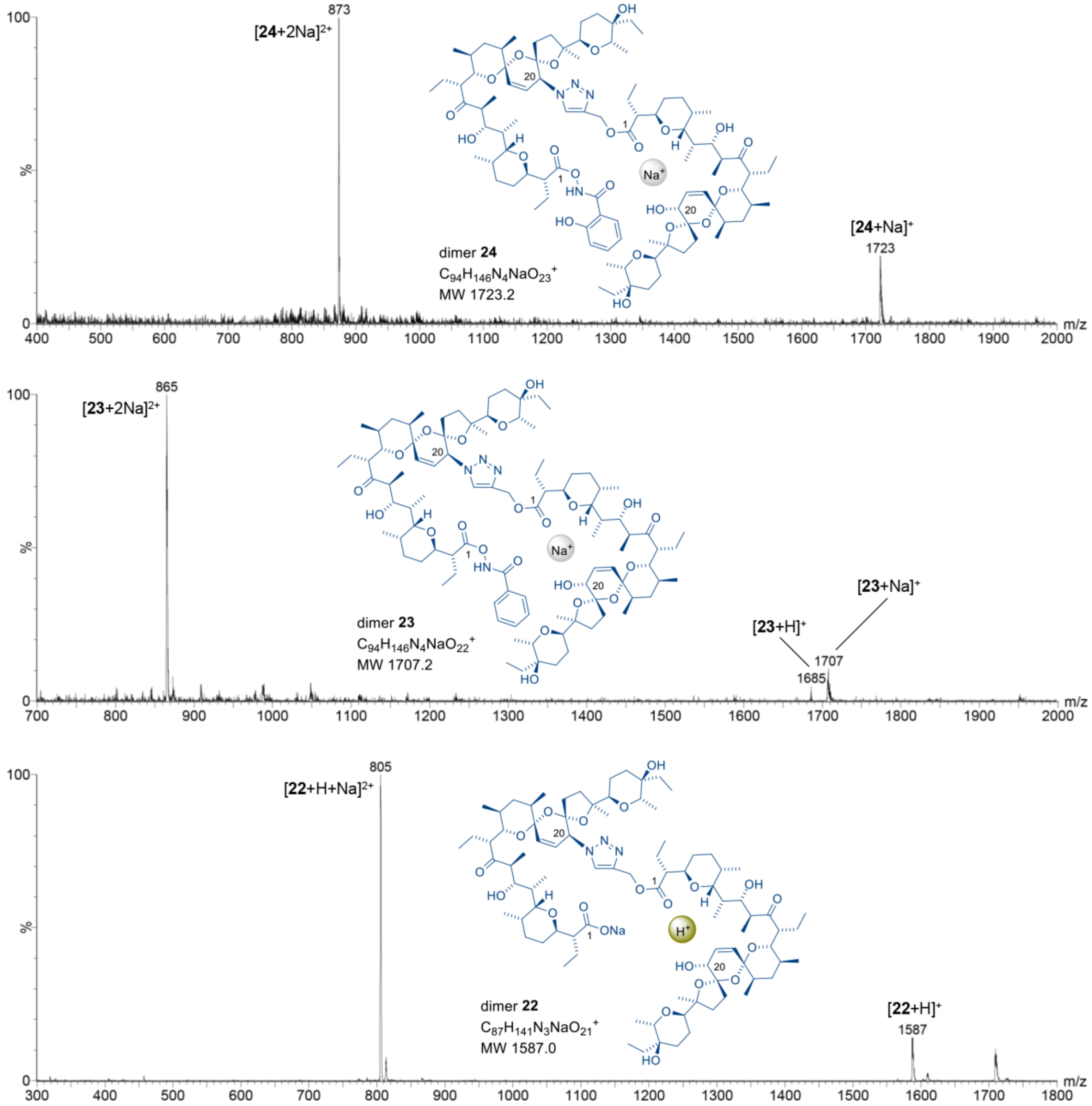

Figure S31. The ESI mass spectra of a mixture of **22**, **23**, and **24** with $NaClO_4$ at cv = 30 V.
